# Western and non-western gut microbiomes reveal new roles of *Prevotella* in carbohydrate metabolism and mouth–gut axis

**DOI:** 10.1038/s41522-021-00248-x

**Published:** 2021-10-07

**Authors:** Vishnu Prasoodanan P. K., Ashok K. Sharma, Shruti Mahajan, Darshan B. Dhakan, Abhijit Maji, Joy Scaria, Vineet K. Sharma

**Affiliations:** 1 MetaBioSys Group, Department of Biological Sciences, Indian Institute of Science Education and Research Bhopal, Bhopal, Madhya Pradesh 462066 India; 2 Department of Animal Science, Department of Food Science and Nutrition, University of Minnesota, Saint Paul, MN 55455 USA; 3 Behaviour and Metabolism Laboratory, Champalimaud Research, Champalimaud Centre for the Unknown, Lisbon, 1400-038 Lisboa Portugal; 4 Animal Disease Research & Diagnostic Laboratory, South Dakota State University, Brookings, SD 57007 USA

**Keywords:** Metagenomics, Microbiome

## Abstract

The abundance and diversity of host-associated *Prevotella* species have a profound impact on human health. To investigate the composition, diversity, and functional roles of *Prevotella* in the human gut, a population-wide analysis was carried out on 586 healthy samples from western and non-western populations including the largest Indian cohort comprising of 200 samples, and 189 Inflammatory Bowel Disease samples from western populations. A higher abundance and diversity of *Prevotella copri* species enriched in complex plant polysaccharides metabolizing enzymes, particularly pullulanase containing polysaccharide-utilization-loci (PUL), were found in Indian and non-western populations. A higher diversity of oral inflammations-associated *Prevotella* species and an enrichment of virulence factors and antibiotic resistance genes in the gut microbiome of western populations speculates an existence of a mouth-gut axis. The study revealed the landscape of *Prevotella* composition in the human gut microbiome and its impact on health in western and non-western populations.

## Introduction

*Prevotella* is a highly diverse genus that exhibits compositional variations in both inter-individual and inter-population comparisons of human gut microbiome^1^. The analysis from multiple western populations found *Prevotella* dominating the enterotype-2 among the three enterotypes identified by Arumugam et al., whereas *Bacteroides* and *Ruminococcus* dominated the other two enterotypes^1^. The meta-analysis of Indian samples also reaffirmed the association of *Prevotella* with enterotype-2^1,2^. The *Prevotella* species in rumen and hindgut are known to possess extensive repertoires of polysaccharide utilization loci (PULs) and carbohydrate-active enzymes for the metabolism of various plant polysaccharides^3^. Thus, it displays a positive association with diets rich in plant-derived fibers and carbohydrates and a negative association with fatty and amino acid-rich diets, and is also shown to decrease on the consumption of animal-based diet in vegetarian subjects^4–7^. These observations highlight the significance of the *Prevotella* genus as a key player in the human gut microbiome.

*Prevotella copri* is the most well-studied and abundant intestinal species in the *Prevotella* genus. One of the key reasons for its abundance in the human gut is the preferential metabolism of xylan, a plant polysaccharide found in plant-based diets, by this species^8^. The prevalence of carbohydrate metabolism genes in *P. copri* also confirmed its association with vegan dietary habits^9^. Recent investigations revealed the high prevalence of *P. copri* in the gut microbiomes of selected non-western populations^10,11^ including the local people of Betsimisaraka and Tsimihety ethnic origins from Madagascar cohort^12^, Matses and Tunapuco communities of Peru and hunter-gatherers of Tanzania^13,14^, and BaAka rainforest hunter-gatherers of Central African Republic^15^. A recent metagenomic study from India comprising of 110 individuals and other 16S rDNA amplicon-based studies also revealed a strong association between *P. copri* and plant-based diet in Indian population^2,16–18^. In contrast, the gut microbiome of western populations such as US, Spain, and migrant individuals to the US that consume a typical westernized diet was mainly enriched in *Bacteroides*, *Ruminococcus* and showed a very low abundance of *Prevotella*^19,20^. The Italian vegan and vegetarian samples consuming a diet rich in plant-based components showed a higher abundance of *P. copri* compared to the other western populations^9,21^, though they still clustered with the western populations^9^. Thus, the observed lower and higher abundance of *Prevotella* in western and non-western populations, respectively, may further emphasize the crucial role of diet in selecting and shaping the abundance of *Prevotella* in the human gut.

The human oral cavity also hosts an enormous diversity of *Prevotella* spp., which is prevalent in almost 85% of western and 100% of non-western populations with an average abundance of 7.4% and 11.5%, respectively^22^. Interestingly, the oral *Prevotella* spp. were also found in the stool microbiome and the oral and gut strains were mostly similar within a host suggesting an oral–gut route/axis^23^. Though most of the *Prevotella* species colonizing different human mucosal sites such as oral and gut tissues have been considered as commensals, some species show pathobiontic properties and have been found to be involved in opportunistic infections^24^. The initial human microbiome studies found an association between the higher abundance of several oral-associated *Prevotella* species such as *P. intermedia* and *P. nigrescens* with localized and systemic diseases including periodontitis, bacterial vaginosis, rheumatoid arthritis, metabolic disorders, and Inflammatory bowel disease^25–29^. Recently, the involvement of “mouth–gut axis” in the pathogenesis of gastrointestinal diseases such as IBD and colorectal cancer have emerged^22,23,30,31^. The ingested oral bacteria translocate to the lower digestive tract and induce gut inflammation that likely disrupts colonization resistance mediated by the commensal gut microbiota making it possible for oral pathobionts to ectopically colonize the gut which supports the mouth–gut axis hypothesis. By contrast, the recent metagenome-based studies testify that *P. copri* is a gut commensal and is not associated with inflammation in the human gut^11^.

Due to the abundance and important role of *P. copri* in the human gut, extensive genetic and population genomics studies have been carried out that suggested a classification of this species in four different clades^11,32^. Unlike *P. copri*, a similar depth of knowledge on the abundance and role of other gut commensal and pathobiont *Prevotella* species in human health is largely missing in different populations. Further, the gut microbiome of the Indian population, which is known to be the most enriched for *Prevotella* spp., has not been included in any previous *Prevotella*-focused study^2,17,18^. Thus, in this study, a comprehensive analysis of the composition, diversity, and functional role of *Prevotella* species in the gut microbiome was carried out in *Prevotella*-rich non-western populations (Madagascar, Tanzania, and Peru)^10,12–14,33^ including the largest gut metagenome of the Indian population that primarily consume plant-based diets, and western populations (US, Spain, Netherlands, and Italy)^9,19,34^ that primarily consume the animal-based diets. Classification of populations as “western” or “non-western” was made on the basis of traditional lifestyle, diet, and geographic and sociodemographic definitions^10,35^. To examine the association of *Prevotella* with inflammatory bowel disease (IBD)^25,36^, including ulcerative colitis and Crohn’s disease, the results were further compared with IBD cohorts from the US, Netherlands, and Spain^19,34^. This study provided new insights into the role of diversity, composition, and function of *Prevotella* in the gut microbiome and their impact on human health.

## Results

### Abundance of *Prevotella* in western and non-western populations

The gut microbiome samples from populations that have a higher abundance of the *Prevotella* genus in their gut including the largest available cohort of 200 healthy samples from different locations and age groups in India, and samples of the healthy individuals from Madagascar (*n* = 112)^10,12^, Tanzania (*n* = 67)^14,33^, and Peru (*n* = 36)^13^ were analyzed in this study. By contrast, samples from populations containing a much lower abundance of *Prevotella* in the gut microbiome of healthy individuals including Italy (*n* = 101)^9^, USA (*n* = 34)^34^, Netherlands (*n* = 22)^34^ and Spain (*n* = 14)^19^, were selected for the comparative analysis. To examine the association of *Prevotella* with gut inflammation, we also analyzed samples from patients with IBD from the USA (*n* = 121)^34^, the Netherlands (*n* = 43)^34^, and Spain (*n* = 25)^19^ (Supplementary Note 1). Human gut microbiome composition and abundance of *Prevotella* genus in each population were examined by taxonomic assignment of high-quality sequenced reads (see “Methods” section).

### *Prevotella* is the most abundant genus in the distinct Indian gut microbiome

**Fig. 1:**
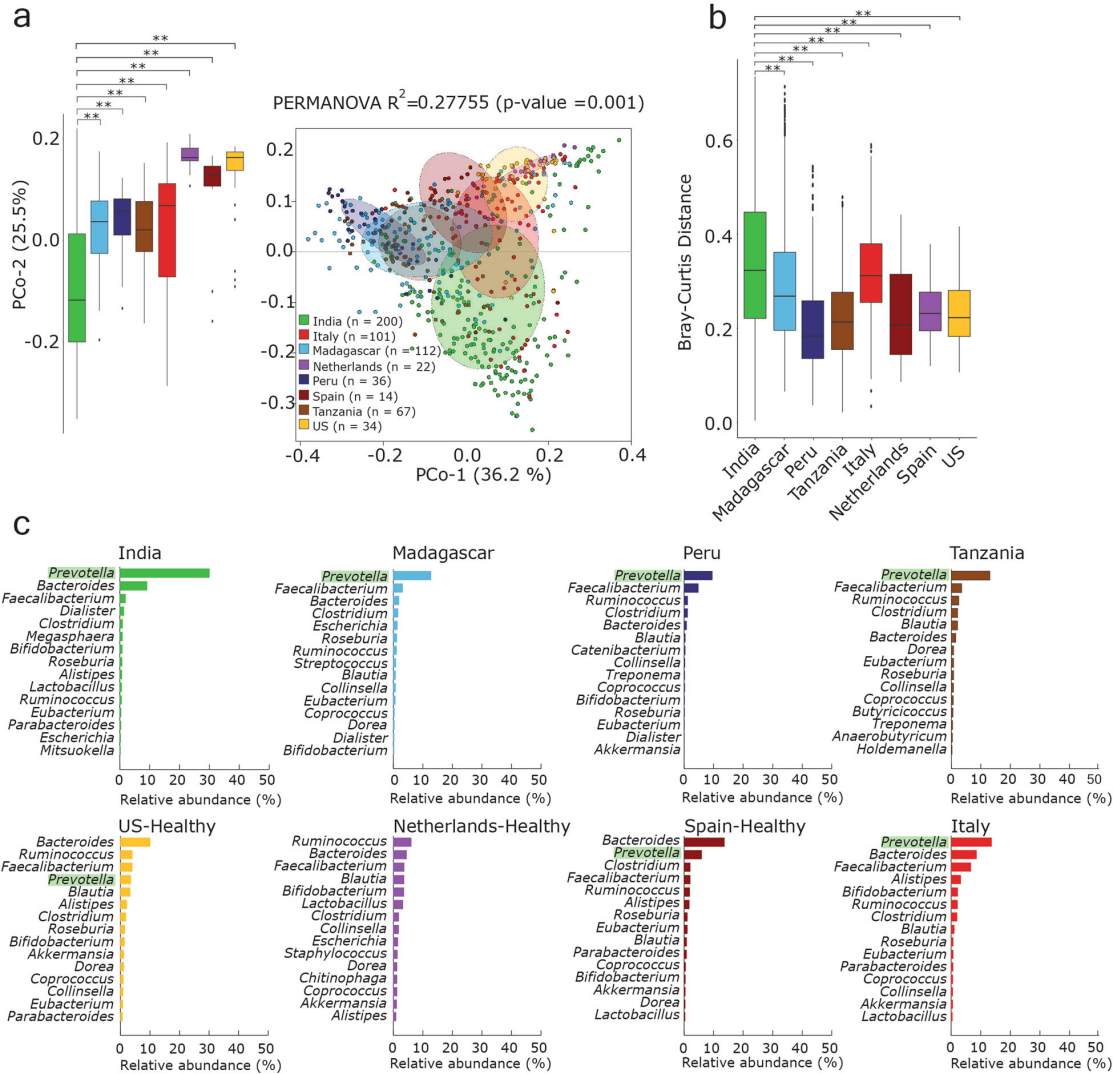
*Prevotella* is the most abundant genus in the distinct Indian gut microbiome. **a** Principal coordinates analysis considering inter-sample Bray–Curtis distance based on species abundance table (obtained from classification of reads using Kaiju). Total number of samples in each data set is given in the left bottom. **b** Box-plot showing inter-sample variation based on the abundance of different bacterial species in each sample (using pairwise Bray–Curtis distance) in each population. **c** Relative abundance of top 15 genera in each healthy population. Relative abundance was calculated after classifying reads at the genus level using Kaiju. *Prevotella* genus is highlighted in a lighter shade of green color. The whiskers, bound of the box, and the line in the middle of the box represent the min-to-max values, 25th–75th percentiles, and median, respectively. Kruskal–Wallis test was used to test the distributions of box plots. ns refers to “not significant”, and ** indicates *p*-value < 0.01.

To investigate the human gut microbiome composition of all populations based on taxonomic assignment of high-quality reads, Principal Coordinates Analysis (PCoA) using Bray–Curtis distances generated from relative abundances of bacterial species was performed. The analysis showed clear distinctions among western and non-western populations (Bray–Curtis, PCoA, PERMANOVA, *R*^2^ = 0.27, *p* = 0.001) (Fig. 1a). Samples from Italy and Spain showed a large overlap with the samples from the US and Netherlands among the western populations; although, a small overlap with Indian and non-western populations was also noticed. A separate and significantly distinct clustering of Indian samples was observed based on Principal coordinate-2. Significantly higher inter-sample variation was also observed in the Indian population when compared to all other populations (Kruskal–Wallis test, *p*-value = 0.01) (Fig. 1a, b).

The relative abundances of the top 15 genera in each population based on the taxonomic annotation of reads revealed *Prevotella* as the most abundant genus in all non-western populations, whereas the western populations were mainly enriched in *Bacteroides* (Fig. 1c and Supplementary Fig. 1a, Supplementary Data 1). Furthermore, the taxonomic classification of contigs (>1000 bp) showed a higher (17–30%) relative abundance of the *Prevotella* genus in non-western populations (India, Peru, Tanzania, and Madagascar) than in western populations (US, Netherlands, Italy, Spain) (Supplementary Fig. 1b).

### New insights from *Prevotella* landscape in western and non-western populations

One of the limitations of the publicly available genome databases is the lack of information on the recently cultured and reconstructed genomes/MAGs (from metagenomes) of *Prevotella*, and do not represent the comprehensive genetic diversity of the *Prevotella* genus/species. Since the estimated diversity of human-associated *Prevotella* spp. is much higher than the available catalog of *Prevotella* isolates, we constructed a *Prevotella* genome database consisting of 2204 genomes including 547 reference genomes of *Prevotella* species retrieved from NCBI, 1612 reconstructed *Prevotella* genomes/bins^10^, 15 *Prevotella* isolates from a previous study^11^, five Asian *Prevotella* isolates from an unpublished study, and 25 reconstructed *Prevotella* bins in this study (see “Methods” section and Supplementary Fig. 2, Supplementary Data 2–4). The abundance of each *Prevotella* genome in human gut samples was calculated by alignment of reads against this *Prevotella* genome database (Supplementary Note 2, Supplementary Figs. 3, 4a, and Supplementary Data 5).

**Fig. 2:**
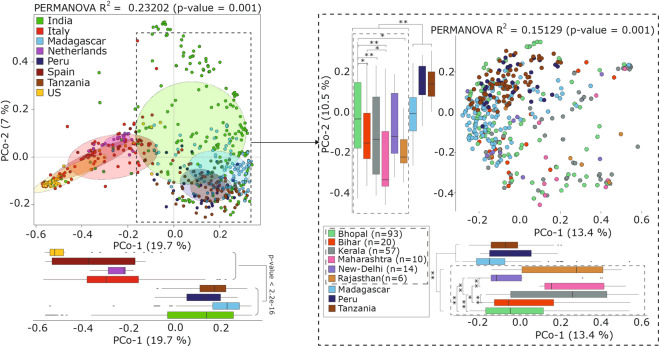
Inter-sample variation of human gut microbiome samples based on *Prevotella* composition. Principal coordinates analysis (PCoA) considering inter-sample Bray–Curtis distance based on the relative abundance of genomes/bins belong to *Prevotella* genus in each population. The PCoA plot represented in dashed rectangle further shows the distribution of samples from different geographical regions in India with samples from non-western populations. A nonparametric two-sided Wilcoxon rank-sum test was used for testing the box-plot distributions. ns refers to “not significant”, * indicates *p*-value < 0.05 and ** indicates *p*-value < 0.01. The whiskers, bound of the box, and the line in the middle of the box represent the min-to-max values, 25th–75th percentiles, and median, respectively.

Principal coordinates analysis performed using inter-sample Bray–Curtis distance based on the abundance of genomes/bins from *Prevotella* genus (from the *Prevotella* genome database) in each population showed that the first principal coordinate separates the western population from the non-western population. A similar analysis of the Indian population, likewise, found that the first principal coordinate significantly separated the Indian population from other non-western populations. The Indian samples also showed the highest inter-sample variation among the non-western populations, whereas little inter-sample variation was observed in western populations compared to non-western populations. (Fig. 2 and Supplementary Fig. 4b). Further analysis also confirmed that the higher inter-sample variation in the Indian cohort was not due to the larger number of samples (Supplementary Note 3).

To examine the association of dietary habits (vegetarian and non-vegetarian) with the composition of the gut microbiome in the Indian population, the principal coordinates analysis based on *Prevotella* Genome Database (PGD) and 1021 *P. copri* genomes indicated that diet significantly explains the variation in samples based on the relative abundance of *Prevotella* genus and *P. copri* species **(**Supplementary Fig. 5a, b). Further, the six different geographical regions represented in the Indian population also showed significantly higher inter-sample variations between them based on PCo-1 and PCo-2 (PERMANOVA, *R*^2^ = 0.07, *p*-value = 0.001) (Supplementary Fig. 6). Similarly, in the Italian cohort with distinct dietary habits (vegans, vegetarians, and omnivores), the analysis of relative abundance of genomes/bins in PGD and *P copri* genomes revealed that diet significantly affect the variation in samples in the case of *P. copri* composition (PERMANOVA, *R*^2^ = 0.04442, *p*-value = 0.002) (Supplementary Fig. 5c, d). The correlation analysis between *Prevotella* genomes in each population showed the highest co-occurrence of *Prevotella* genomes in non-western populations and in the Italian population. By contrast, a negligible number of significant positive correlations were observed in the US and the Netherlands populations, and no significant correlation was observed in the Spain population (Supplementary Data 6).

### Higher abundance and genome-relatedness of *P. copri* in Indian and non-western populations

We clustered the 2204 *Prevotella* genomes based on a distance cut-off of 0.00 (100% ANI) that resulted in 2204 clusters indicating that no two genomes/bins are 100% identical. Clustering based on the distance cut-off of 0.05 (95% ANI; species-level clustering) resulted in 228 clusters (Supplementary Note 4, Supplementary Figs. 7–10, and Supplementary Data 7). 102 *Prevotella* genomes/bins with ‘indval’ score >0.60 (*p*-value < 0.01) were differentially abundant in western and non-western populations and were considered for further analysis. Of the 102 *Prevotella* genomes/bins, 26 were differentially abundant in non-western populations including 18 bins reconstructed from metagenomic data sets, and the remaining eight genomes/bins included four *Prevotella* isolates (out of five from Asia), and four NCBI reference genomes including *P. copri* (NCBI Accession: GCA 002224675.1) (Supplementary Fig. 11 and Supplementary Note 4). By contrast, 76 *Prevotella* genomes/bins that were found differentially abundant in western populations were also known in the NCBI database and included *P. marseillensis*, *P. lascolaii*, *P. ihumii,* and various strains of *P. intermedia*.

**Fig. 3:**
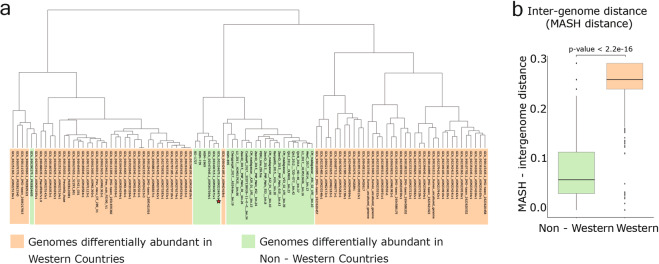
Significantly lower intergenomic distance of differentially abundant *Prevotella* genomes in non-western populations. **a** Cladogram constructed based on the intergenome distance (MASH-distance) between genomes/bins differentially abundant in non-western and western populations. Text highlighted in a lighter shade of green color are differentially abundant *Prevotella* genomes in non-western populations, and the text highlighted in orange color are differentially abundant *Prevotella* genomes in western population. The *Prevotella* genome highlighted using red-colored star is of *P. copri* (Assembly accession: GCA_002224675.1). **b** Box plots show the intergenome distance of differentially abundant *Prevotella* genomes in non-western and western populations. The pair of entries that showed intergenome distance = 1 were excluded from this plot. The whiskers, bound of the box, and the line in the middle of the box represent the min-to-max values, 25th–75th percentiles, and median, respectively. Significance levels were evaluated using Wilcoxon rank-sum test.

Most of the differentially abundant *Prevotella* genomes found in western populations belonged to unclassified *Prevotella* genomes in the NCBI microbial genome assembly database (Supplementary Fig. 12). The intergenome distances between the 102 differentially abundant genomes identified from the above analysis indicated that 25 of the 26 differentially abundant *Prevotella* genomes in non-western population were from species closely related to *P. copri* or were subspecies of *P. copri* as they were found on the same branch (Fig. 3a). Further, 29 differentially abundant *Prevotella* genomes were found in the Indian population compared to all other populations using labdsv (indval score >0.50, *p*-value = 0.01), of which 23 were identified as *P. copri* by taxonomic assignment using BAT (Supplementary Note 5). The average intergenome distance between differentially abundant genomes in western and non-western populations are 0.26 and 0.08, respectively (the genome pairs having MASH distance = 1 omitted). Taken together, these findings indicate that the differentially abundant *Prevotella* genomes in non-western populations are related to each other, whereas those in western populations are more diverse (Fig. 3b and Supplementary Data 8).

### Clade composition of *P. copri* strains in Indian and other non-western populations

Principal coordinates analysis based on the abundance of *P. copri* genomes/bins indicated higher inter-sample variation in the Indian population than in other non-western populations (Supplementary Note 6). Both PCo-1 and PCo-2 significantly explain the distinctness and inter-sample variation of the Indian population compared to all other populations. To further explore the diversity of *P. copri* strains in non-western populations, the strain level composition of *P. copri* in non-western populations were analyzed using 1021 reconstructed bins of *P. copri* with their clade classification information (clade A, B, C and D, see “Methods” section) (Supplementary Fig. 13a). The *P. copri* clades in the Indian population showed a similar distribution as those in non-western populations: >70% of genomes from clades C and D were present in >50% of samples in all non-western populations. By contrast, only 10% of genomes from clades C and D were present in western populations. The comparison of the average relative abundance of each of the four clades revealed a highest abundance of clade D followed by clade C in all non-western populations (Supplementary Fig. 13b).

The clade composition of *P. copri* strains in the Indian population was further examined by analyzing 42 reconstructed *P. copri* specific bins and five isolates (see “Methods” section, and Supplementary Fig. 14a, Supplementary Data 9). As a reference for the clade assignment, we used 72 high-quality *P. copri* genomes/bins reported previously^11^. The pairwise intergenomic distances of each genome/bin were calculated, and the MASH distance-based clustering resulted in 9 bins assigned to clade C, 7 bins assigned to clade B, and 5 bins assigned to clade A. The remaining 26 of the 47 bins formed a separate cluster with a higher intergenomic distance between each other. Further, this cluster contained none of the 72 high-quality *P. copri* bins used as a reference (Supplementary Fig. 14b), indicating that these 26 bins may be other subspecies or strains of *Prevotella*.

### Examining the association of *Prevotella* species/strains with IBD cohorts

**Fig. 4:**
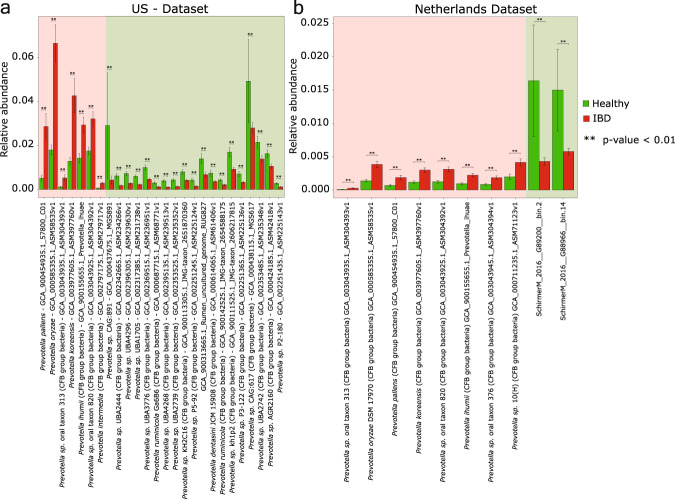
Abundance of oral inflammation-associated *Prevotella* species/strains in IBD cohorts. **a** Representation of differentially abundant *Prevotella* genomes in healthy and IBD cohorts of US population. Genomes highlighted in a lighter shade of red color are differentially abundant in the IBD cohort, and genomes highlighted in a lighter shade of green color are differentially abundant in the healthy cohort. **b** Representation of differentially abundant *Prevotella* genomes in healthy and IBD cohorts of Netherlands population. Genome highlighted in a lighter shade of red color are differentially abundant in IBD cohort, and genomes highlighted in a lighter shade of green color are differentially abundant in the healthy cohort. *Prevotella* genomes/bins with “indval” score >0.60 and *p*-value < 0.01 reported by labdsv are represented in the bar-plots. Error bars represent plus or minus one standard error of the mean.

To examine the association of *Prevotella* with IBD, we compared the *Prevotella* genomes of healthy individuals and patients with IBD in the US and Netherlands populations by using labdsv and found 30 genomes that differed in their abundance. Eight of these 30 genomes were significantly abundant in IBD, of which seven were present in the US, and six of these were also present in Netherlands data sets. These seven genomes were those of *P. pallens*, *P. oryzae*, *P. koreensis*, *P. ihumii*, *P. intermedia*, and two unclassified *Prevotella* strains (*Prevotella sp. oral taxon 820* and *Prevotella* sp. *oral taxon 313*) (Fig. 4), which have been associated with oral inflammatory conditions in previous studies (Supplementary Note 7)^37–41^. We also compared the relative abundance of these 30 *Prevotella* genomes in healthy western and non-western populations and found that they were significantly less abundant (*p*-value < 0.01) in non-western populations (Supplementary Fig. 15a). Principal coordinates analysis revealed a clear separation between western and non-western populations, and the variance explained by PCo-1 increased to 31.6% using relative abundance of these 30 genomes (Supplementary Fig. 15b). In addition, the classification of samples from western and non-western populations using randomForest based on the 30 differentially abundant genomes resulted in high accuracy (AOC = 0.92) (Supplementary Fig. 15c). The differentially abundant *Prevotella* genomes in western IBD patients also showed higher abundance in the western-healthy population in comparison with non-western-healthy populations (Supplementary Fig. 16).

### Functional composition of *Prevotella* genus in healthy and IBD cohorts

**Fig. 5:**
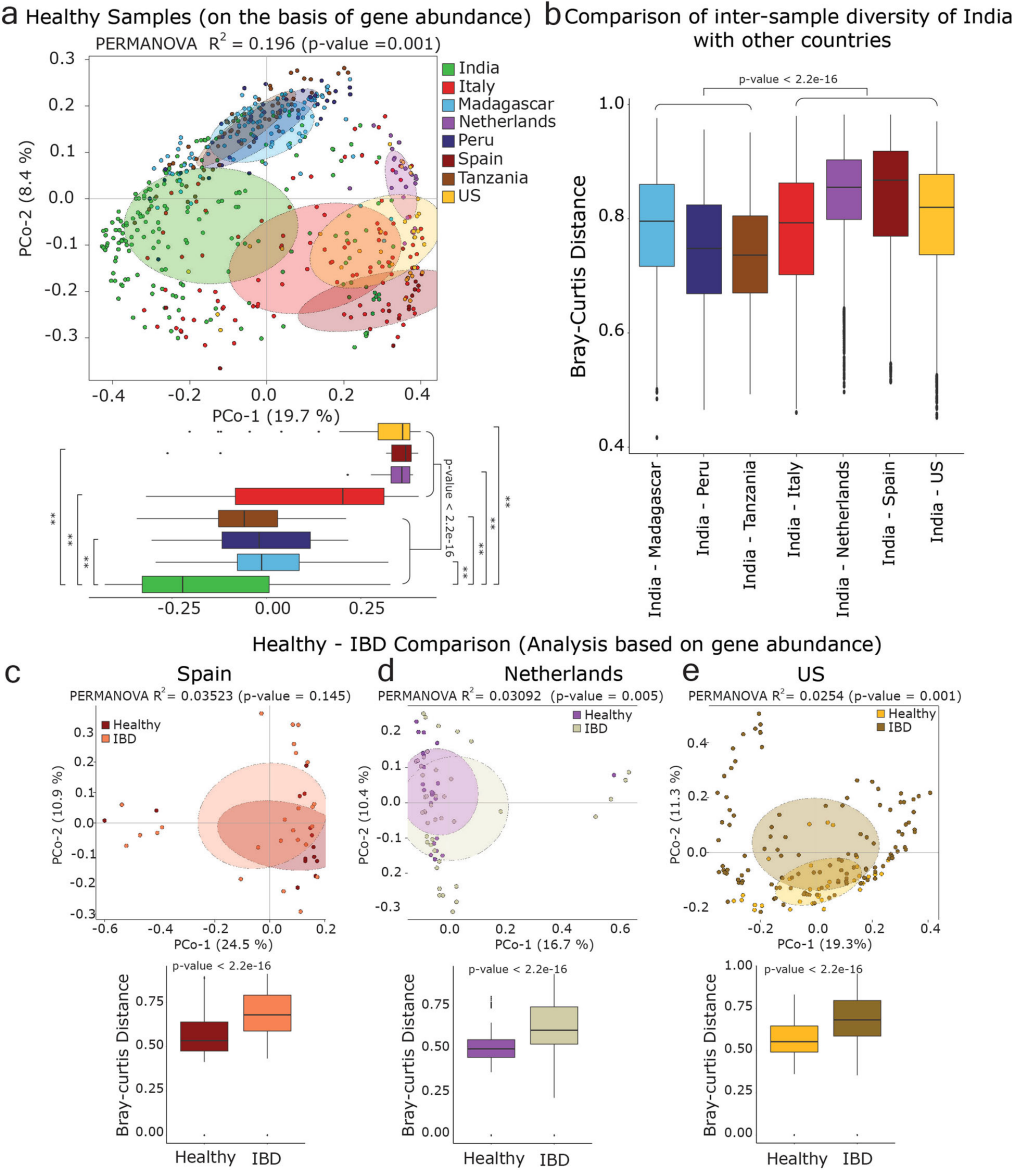
Functional composition of *Prevotella* genus in healthy and IBD cohorts. **a** Principal coordinates analysis considering inter-sample Bray–Curtis distance based on the relative abundance of genes from PGC (*Prevotella* Gene Catalog) in healthy populations. The figure shows western and non-western populations are significantly separated based on the first principal coordinate. The figure also shows that the Indian population has a significantly distinct *Prevotella* gene composition. **b** Box plot showing the inter-sample distance (Bray–Curtis) of Indian samples with other populations based on the relative abundance of genes of PGC. **c**–**e** Principal coordinates analysis considering inter-sample Bray–Curtis distance based on the relative abundance of genes of PGC in western-healthy and IBD samples (Spain, Netherlands and US, respectively). Box plots of inter-sample distance in healthy and IBD samples are shown at the bottom part of each PCoA plot. The whiskers, bound of the box, and the line in the middle of the box represent the min-to-max values, 25th–75th percentiles, and median, respectively. Nonparametric two-sided Wilcoxon rank-sum test was used to test the box-plot distributions.

We constructed a catalog of the 2,992,963 non-redundant *Prevotella* genes identified in this study (see “Methods” section, Supplementary Data 10). Abundance of *Prevotella* gene catalog (PGC) in each sample was quantified and analyzed further (see “Methods” section). The *Prevotella* genes in the Indian population formed a separate cluster, but showed a small overlap with the populations from Italy, Madagascar, Peru, and Tanzania perhaps due to high inter-sample variation (PERMANOVA *R*^2^ = 0.196, *p*-value = 0.001). The first principal coordinate showed significant separation of the Indian samples from the other populations, and the inter-sample variation in the Indian and Italian populations was the highest of all the populations (Fig. 5a). Also, we observed a lower average inter-sample distance between the populations from India and Tanzania, and India and Peru when compared to the other populations, which indicates functional relatedness of *Prevotella* in Indian and these non-western populations (Fig. 5b). Further, based on gene abundance analysis of PGC, the comparison of IBD and healthy samples in western populations i.e., Spain (Fig. 5c), Netherlands (Fig. 5d), and US (Fig. 5e) showed significantly higher inter-sample variation in IBD compared to healthy samples. The beta-diversity analysis of KEGG KO-based functional classes in healthy and IBD samples also showed a similar result as observed in the case of gene abundance analysis in western populations. The Indian population was relatively enriched in genes involved in branched-chain amino acid biosynthesis when compared to western populations. Likewise, it was relatively enriched in genes involved in proline, histidine, and lysine biosynthesis, as were the populations from Peru and Tanzania (Supplementary Fig. 17 and Supplementary Data 11).

### Abundance of plant carbohydrate metabolizing enzymes in Indian and other non-western populations

Carbohydrate metabolism is a key function of several dominant *Prevotella* species in the gut, thus we compared the abundance of genes involved in carbohydrate metabolism in the *Prevotella* genomes from the various populations using CAZy (carbohydrate-active enzymes) database^42^ (details in Supplementary Note 8). Principal coordinates analysis based on the abundance of CAZy genes resulted in clustering of the Indian and Tanzanian populations (Supplementary Fig. 18a, b).

The correlation between the relative abundance of *Prevotella* genomes/bins and CAZy families in each population was analyzed using the ccrepe package. All non-western populations and the Italian population showed a higher number (54–1292) of significant positive correlations (*r*-value > 0.5, *p*-value < 0.01), whereas no significant positive correlations were found in the Netherlands and the US populations. This suggests that the genes encoding carbohydrate metabolizing enzyme families are associated with the abundance of *Prevotella* genomes in non-western populations (Supplementary Fig. 19).

**Fig. 6:**
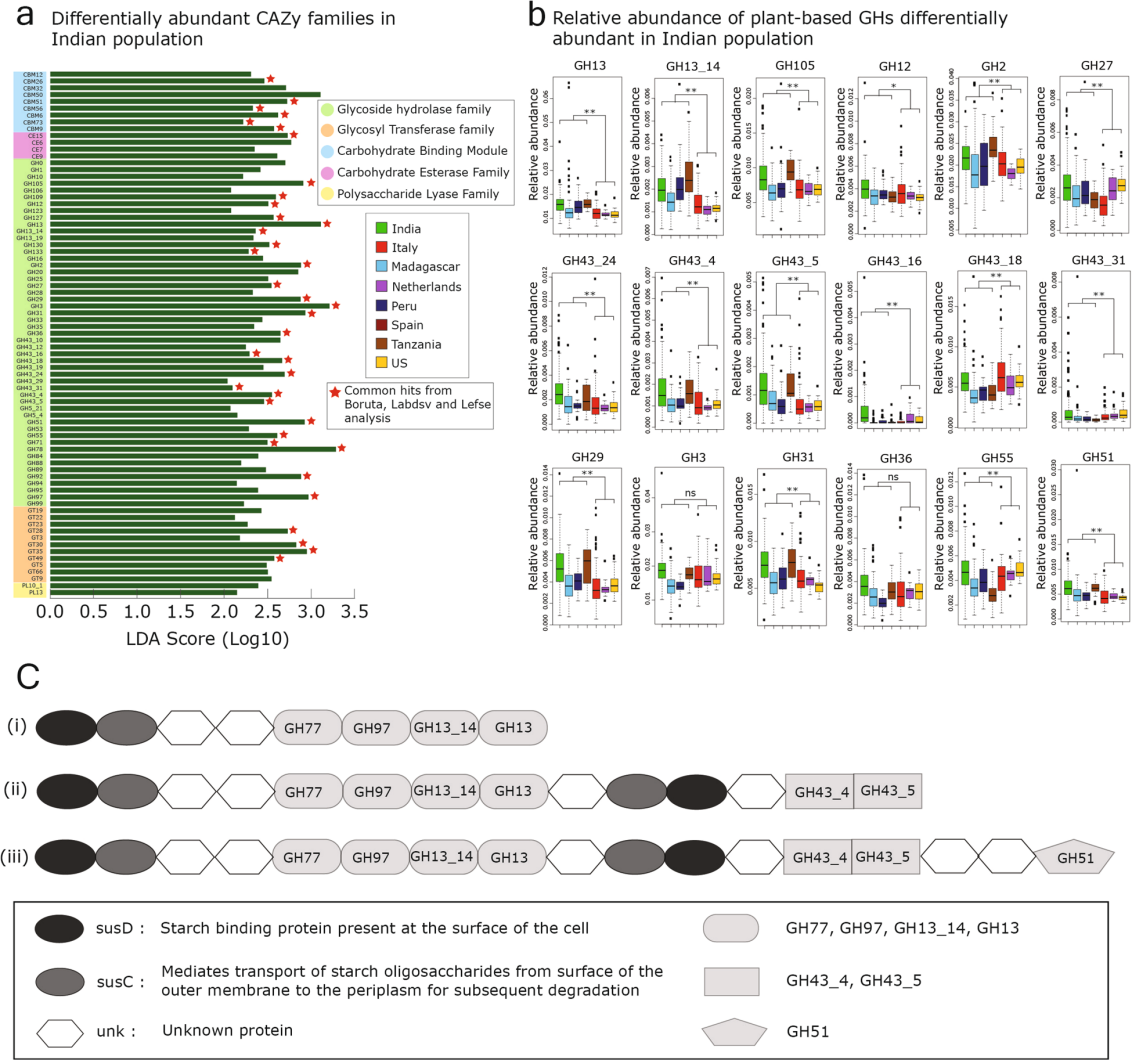
Abundance of plant carbohydrate metabolizing enzymes of *Prevotella* genus in Indian and other non-western populations. **a** Discriminating carbohydrate metabolizing gene families (CAZy families) of *Prevotella* in the Indian cohort compared to all other populations together (Italy, Netherlands, US, Madagascar, Peru, and Tanzania) identified based on LDA score (using LEfSe). The legend shown on the right side depicts the broad classification of CAZy families. The CAZy families highlighted with a red-colored star are the ones that were detected in all three statistical methods for finding differentially abundant CAZy families (LEfSe, labdsv, and boruta). **b** Relative abundance of all differentially abundant plant-based Glycosyl Hydrolases (based on studies mentioned in Supplementary Data12, sheet 2) in all populations. The whiskers, bound of the box, and the line in the middle of the box represent the min-to-max values, 25th–75th percentiles, and median, respectively. A nonparametric two-sided Wilcoxon rank-sum test was used to test the box-plot distributions. ns refers to “not significant”, * indicates *p*-value < 0.05 and ** indicates *p*-value < 0.01. **c** Pullulanase-containing-PULs were detected in 782 out of 1023 *P. copri* genomes. The most frequent PUL among them is susD-susC-unk-unk-GH77-GH97-GH13_14-GH13 shown as (i) is present in 113 genomes (with the same arrangement of CAZy families). The second most frequent pullulanase-containing-PUL has GH43_4 and GH43_5 shown as (ii) with the aforementioned CAZy gene families and is present in 93 genomes (with the same arrangement of CAZy families). Other pullulanase-containing-PULs have GH51 with GH77, GH97, GH13_14, GH13, GH43_4, and GH43_5 at the same genomic loci shown in (iii) and are present in 21 genomes (with the same arrangement of CAZy families). The majority of the remaining pullulanase-containing-PULs have the same gene families with different arrangements (see Supplementary Notes-part 10 and 11).

To compare the carbohydrate metabolism genes of *Prevotella* in healthy samples from different populations, we calculated the relative abundance of carbohydrate metabolizing enzyme (CAZy) families categorized as glycosyl hydrolases (GHs), glycosyltransferases (GTs), carbohydrate-binding modules (CBMs), carbohydrate esterases (CEs) and polysaccharide lyases (PLs). We identified the core CAZy families (present in >80% of samples) in *Prevotella* genomes, and found 78 GHs, 26 GTs, 29 CBMs, 12 CEs, and 8 PLs. A total of 37 CAZy families and subfamilies were identified as differentially abundant in *Prevotella* species in the Indian population compared to other healthy populations (Methods and Supplementary Note 8). Interestingly, out of 37 CAZy families 26 are GH family/subfamily of enzymes that are involved in hydrolysis or rearrangement of glycosidic bonds and are major contributors to carbohydrate degradation. The genes encoding differentially abundant GHs were classified into three groups based on their utilization of carbohydrate substrates of plant, animal, and mucin origin^33,43^ (Supplementary Data 12). 73% (19 out of 26) of the differentially abundant GH family/subfamily of enzymes in the Indian population belonged to the group that uses plant-based carbohydrates (Fig. 6a). Among these 19 CAZy families, ten were also significantly abundant in all non-western populations (Wilcoxon rank-sum test, *p*-value < 0.01), and displayed the highest abundance in the *Prevotella* genomes from the Indian and Tanzanian population suggesting a relatedness between these two populations (Fig. 6b).

Further, the LEfSe and labdsv analysis revealed that 15.8% and 11.5% of CAZy families, respectively, were common in the non-western population and Indian population (Supplementary Fig. 20). In contrast, 8.9% (identified by LEfSe) and 6.6% (identified by labdsv) CAZy families were common in the western and Indian population. These observations indicate that a relatively higher number of CAZy families were commonly present in the *Prevotella* genomes of Indian and non-western populations as compared to Indian and western populations, and further supports the relatedness of carbohydrate metabolizing activity in Indian and non-western populations (Supplementary Fig. 20, Supplementary Note 8 and Section 9).

### Abundance of pullulanase-containing PULs in *P. copri* genomes and Indian population

We further examined the abundance of Polysaccharide Utilization Loci (PULs) in *Prevotella* genomes since these loci encode the necessary machinery for carbohydrate metabolism, and usually occur as groups in close proximity to one another in bacterial genomes. We predicted a total of 37,389 PULs in 2204 genomes in the *Prevotella* Genome database (PGD) by using PULpy based on susC/susD-like pairs^44^. Of these 2204 genomes, 2197 were predicted to have at least one PUL. The greatest number of PULs per genome was 60 for an unclassified *Prevotella* genome (GCA-003638705.1-ASM363870v1). The number of PULs per genome was significantly higher (Wilcoxon rank-sum test, *p*-value = 0.015) in the differentially abundant *Prevotella* genomes in non-western populations compared to western populations. (Supplementary Fig. 21 and Supplementary Data 13).

One of the key findings of the study emerged from the analysis of PULs in *P. copri* genomes, which revealed that 77.6% (794 out of 1023) of the known *P. copri* genomes contained pullulanase gene located in PULs. The pullulanase enzyme (GH13_14) acts on α-1,6-linkages within starch (a common plant polysaccharide) and pullanan (a fungal polysaccharide). Operon prediction analysis of contigs having pullulanase-containing-PULs revealed that neopullulanase-susA (Pullulan hydrolase type I) and pullulanase genes are present in the same operon (>95% probability) and are involved in the metabolism of α-1,4 and α-1,6-linkages, respectively, present in starch-derived glucans.

Interestingly, a majority (98.49%) of the PULs containing pullulanase gene also had other CAZy families including GH77, GH97, GH13_14, and GH13 in the same loci, and a small fraction (71.66%) of these PULs also contained GH43_4 and GH43_5 along with the aforementioned CAZy families. 21.16% PULs contain GH51 with all six above-mentioned CAZy families (Fig. 6c). The enzymes in GH77, GH97 GH13_14, and GH13 CAZy families are mainly involved in metabolizing α-1,4 and α-1,6-linkages in starch, whereas the GH43_4, GH43_5, and GH51 subfamilies comprise a range of debranching enzymes that aid in the degradation of arabinoxylans and pectin that are the major non-starch plant polysaccharides. The genomic loci containing pullulanase-containing-PULs extracted from the 782 *P. copri* genomes were analyzed, and it revealed a cluster of genes involved in starch and non-starch metabolism, and also had several hypothetical genes. Further, multiple copies of “TonB-dependent receptor (SusC)” were also noted in these loci (Supplementary Note 10 and Section 11).

Notably, 23 out of the 29 differentially abundant *Prevotella* genomes in the Indian population were *P. copri*, of which 16 (76.2%) also had PULs containing pullulanase enzyme (GH13_14). Similarly, 24 out of 26 differentially abundant *Prevotella* genomes in non-western population have pullulanase-containing-PULs, of which 18 genomes also contained GH43_4, GH43_5, and GH51 families in the pullulanase-containing-PULs (Supplementary Note 12). By contrast, in the western population that had a poor abundance of *P. copri*, only 29% (22 out of 76) differentially abundant *Prevotella* genomes had pullulanase (GH13_14). Taken together, these findings reveal the key role of pullulanase-containing-PULs associated with *P. copri* genomes in the metabolism of starch and non-starch components of dietary cereal grains in Indian and other non-western populations.

### Abundance of virulence factors and antibiotic resistance genes in *Prevotella* in western populations

**Fig. 7:**
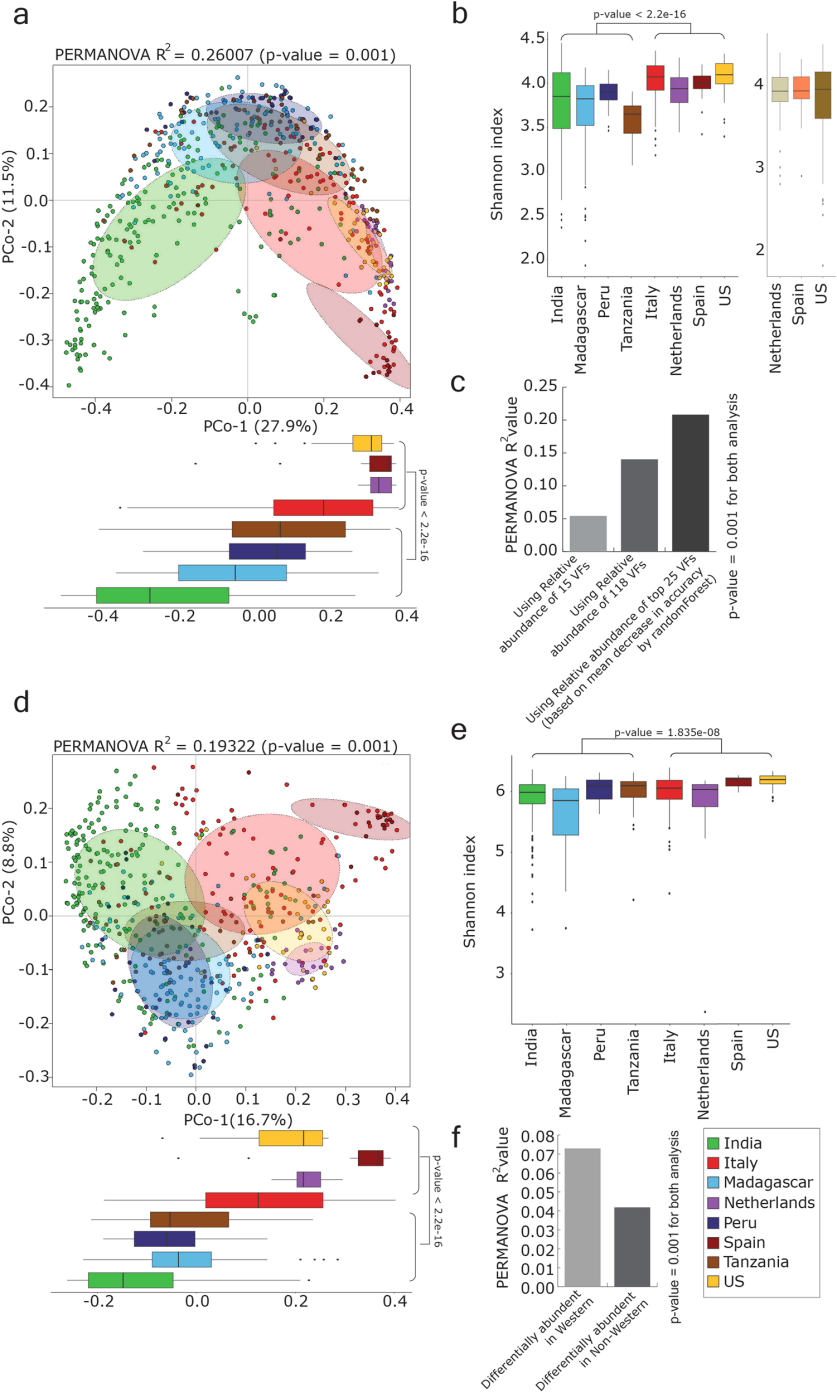
Distribution of virulence factors and antibiotic resistance genes of *Prevotella* genus in different populations. **a** Principal coordinates analysis considering inter-sample Bray–Curtis distance based on the relative abundance of VFs present in *Prevotella* genomes. **b** Alpha-diversity measure (using Shannon index) of VFs present in *Prevotella* genomes in all populations (healthy and IBD). Shannon index was calculated based on the abundance of genes that showed best hits after homology search against full VFDB proteins. **c** Increase in PERMANOVA *R*^2^ values using the relative abundance of 15 VFs showed higher abundance in western populations, 118 VFs showed higher abundance in non-western populations, and 25 differentially abundant VFs in western populations. **d** Principal coordinates analysis considering inter-sample Bray–Curtis distance based on the relative abundance of Antibiotic Resistance Genes (ARGs) present in *Prevotella* genomes (using loose parameter in RGI). **e** Alpha-diversity measure (using Shannon index) of *Prevotella* ARGs in all healthy populations. Shannon index was calculated using the abundance of genes predicted using RGI. **f** Increase in PERMANOVA *R*^2^ values using the relative abundance of differentially abundant *Prevotella* ARGs in the western populations. The whiskers, bound of the box, and the line in the middle of the box represent the min-to-max values, 25th–75th percentiles, and median, respectively. Nonparametric two-sided Wilcoxon rank-sum test was used for testing the box-plot distributions. ns refers to “not significant”, * indicates *p*-value < 0.05 and ** indicates *p*-value < 0.01.

In the *Prevotella* genome abundance analyses described above, we noted that *P. pallens*, *P. oryzae*, *P. koreensis*, *P. ihumii*, *P. intermedia,* and two unclassified strains (*Prevotella* sp. oral taxon 820 and *Prevotella* sp. oral taxon 313) were more abundant in samples from patients with IBD than in samples from healthy individuals in western populations. Of these, *P. intermedia*, *P. marseillensis*, and *P. lascolaii*, are key pathogens that cause anaerobic infections in humans^45–47^, and were significantly abundant in western populations. Moreover, virulence-related genes of *P. intermedia* and *P. nigrescens* are more abundant in the oral microbiomes of patients with oral inflammation than in healthy individuals^48^. To investigate whether virulence-related genes are prevalent in gut *Prevotella* species, we searched our *Prevotella* gene catalog for homologies to genes in the bacterial virulence factor databases^49,50^. Principal coordinates analysis based on virulence factor abundance in each population revealed clear segregation of western and non-western populations (Fig. 7a). The presence and abundance of the virulence protein genes (full-data set) in each population showed that western populations contained a significantly higher number of these genes and Shannon diversity index compared to non-western populations (Fig. 7b and Supplementary Fig. 22a). The above observations were confirmed by analyzing the core virulence factor database (Supplementary Fig. 22b). Of the 133 virulence protein genes identified in the core data set (see “Methods” section), 118 had a higher average relative abundance in western populations compared to non-western populations, and the remaining 15 showed higher abundance in non-western populations (Supplementary Fig. 22c). By using labdsv, LEfSe, and boruta, we identified 37 virulence factor genes that discriminate between western and non-western populations, all of which were more abundant in western populations. This finding was supported by the PERMANOVA test based on Bray–Curtis distance using the abundance of discriminating virulence factor genes, which indicated an increment of *R*-squared value (Fig. 7c). Also, classification of western and non-western samples by using randomForest based on the relative abundance of the 37 discriminating virulence factor genes resulted in high classification accuracy (area under ROC = 0.99) (Supplementary Fig. 22d). The randomForest analysis carried out using 15 (out of 133) virulence factor genes that were highly abundant in non-western populations showed lower accuracy of classification (area under ROC = 0.88) (Supplementary Fig. 22e).

Previous studies have shown the co-occurrence of antibiotic resistance genes and virulence determinants in human gut microbiomes^51–53^. Therefore, we examined the presence of antibiotic resistance genes in *Prevotella* genomes in the gut microbiome of all the populations analyzed in this study. Antibiotic resistance genes encoding proteins involved in the inactivation of antibiotics were the most abundant in *Prevotella* genomes, followed by those involved in antibiotic efflux, antibiotic target alteration, and target protection (Supplementary Fig. 23a, b). Inter-sample distance based on the abundance of antibiotic resistance genes predicted by the resistance gene identifier tool^54^ (in “loose” mode) showed the separation of western and non-western samples (Fig. 7d). *Prevotella* genomes from the Spanish population contained the most antibiotic resistance genes, followed by US and Italian populations, when both “strict” and “loose” criteria were applied with the resistance gene identifier. Fewer antibiotic resistance genes were identified in the *Prevotella* genomes from the Indian and Madagascan populations (Supplementary Fig. 23c, d, e). A significant difference between western and non-western populations was observed based on the number of antibiotic resistance genes identified and the Shannon diversity index (Fig. 7e and Supplementary Fig. 23f). PERMANOVA using differentially abundant antibiotic resistance genes showed higher abundance in the western population compared to non-western populations. Higher *R*-squared value obtained using differentially abundant antibiotic resistance genes showed higher abundance in the western population, and these genes also discriminated between western and non-western populations (Fig. 7f and Supplementary Fig. 23g, h).

## Discussion

**Fig. 8:**
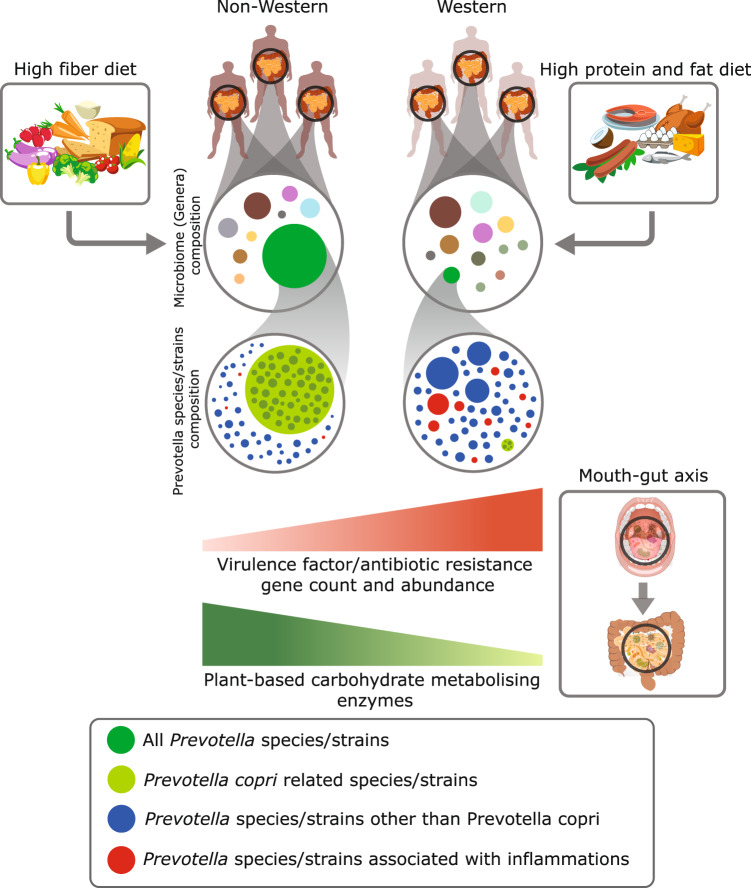
Impact of Prevotella composition on human health. Schematic representation of taxonomic and functional composition of Prevotella species in western and non-western populations is shown.

*Prevotella copri* is the most abundant species from its genus in the human gut microbiome that has attracted most of the global attention^9,11^, whereas, the role of other *Prevotella* species in the human gut and their impact on human health has remained largely unstudied. Moreover, the population-wide *Prevotella*-focused gut microbiome studies have not yet included the Indian population, which has the highest abundance of *Prevotella* genus in healthy individuals. Therefore, we carried out this comprehensive gut microbiome study on a large cohort of healthy samples from various parts of India to gain new insights on the roles of different species of *Prevotella* genus in human health. Secondly, due to the association of *Prevotella* with a high-fiber diet, a comparison was carried out between the *Prevotella*-rich population consuming a high-fiber diet with the populations consuming diets rich in protein and fat and poor in plant-based fibers. Lastly, it was needed to re-examine the association of some species from this genus with gut inflammatory disorders that had been found in some early studies in western populations^24,25^. Therefore, we also carried out a comparative analysis of healthy individuals including both western and non-western populations with IBD data sets (Fig. 8).

The population-wide analysis of the taxonomic composition of the gut microbiome showed clear differences between the western and non-western populations. It also reemphasized the uniqueness of the Indian gut microbiome^2,17,18,55^, with *Prevotella* being the most abundant genus in the Indian population among all analyzed populations. In contrast, the western populations were primarily dominated by *Bacteroides*. The high consumption of a plant-based high-fiber diet is plausibly the primary reason for the high abundance of *Prevotella* in Indian and other non-western populations, in contrast to the consumption of a “typical western diet” in western populations^9^. These results underscore the impact of diet in shaping the gut microbiome of different populations^9,21^.

The construction of a comprehensive *Prevotella* genome database containing 2204 genomes/bins and a *Prevotella* gene catalog containing 2.9 million genes that include the latest information on the recently cultured and metagenomically reconstructed genomes of the *Prevotella* genus were crucial in gaining deeper insights into the functional roles of *Prevotella*. The metagenomic composition of *Prevotella* genomes in PGD revealed the highest inter-sample variation among Indians reasonably attributed to the inclusion of samples from diverse geographical regions of India that prominently differ in their diets and cooking styles. Despite these differences, the Indian population was significantly different from all other populations, yet it was comparatively more related to non-western populations (mainly Tanzania and Peru) than to the western populations (US, Netherlands, Spain, and Italy).

Clues on the existence of several novel strains of *P. copri* in Indian and non-western populations emerged from the analysis of differentially abundant metagenomically reconstructed *Prevotella* genomes that showed lower genetic diversity and close genomic relatedness to *P. copri*. Further, all four major clades of *P. copri*^11^ were highly prevalent in non-western populations, particularly the clades C and D that have a high prevalence of genes encoding enzymes involved in the metabolism of cellulose, hemicellulose, and pectin.

In contrast, the western populations displayed a higher genetic diversity in *Prevotella* species including *P. intermedia*, *P. oris*, *P. oralis*, *P. dentalis* (infection related), *P. bergensis* (infection related), and *P. brevis* (infection-related), which are also reported to be a part of the oral microbiome in western populations and have been associated with oral inflammatory conditions^46,47,56–58^. Similarly, another discriminatory species *P. lascolaii* in western populations was isolated from bacterial vaginosis patients^59^. Notably, the IBD cohort also displayed an abundance of inflammation-associated species such as *P. intermedia*, *P. pallens*, *P. oryzae*, *P. koreensis*, *P. ihumii*, and two unclassified oral strains, which also displayed a significant abundance in western-healthy cohort compared to the healthy non-western populations. In fact, the differences in the abundance of these inflammatory species in western and non-western-healthy populations were sufficient enough to segregate them with high accuracy using randomForest, and may act as *Prevotella* markers to classify these two population groups.

Strong evidence about the involvement of “mouth–gut axis” in gastrointestinal diseases such as IBD and colorectal cancer have recently emerged^23,60^. In the case of newly diagnosed colorectal cancer patients, a higher enrichment of oral species, *P. intermedia* and *P. nigrescens*, has been observed in the gut indicating that these species could be the biomarkers of the oncological condition^61^. In IBD patients, ingested oral bacteria are believed to play a central role in disease pathogenesis by translocating to the lower digestive tract, where the pathobionts can evoke pathogenic immune responses by producing bacteria-reactive CD4^+^ T-cells^62^. Gut inflammation likely disrupts colonization resistance mediated by the resident healthy gut microbiota, making it possible for oral pathobionts to ectopically colonize the gut. Thus, the inflammatory *Prevotella* species of oral microbiome origin could elicit inflammatory conditions in the gut, supporting the mouth–gut axis hypothesis^22,60^. Some recent studies that associated lifestyle factors, particularly the western diet with the abundance of oral pathobiont species indicate the western-association of this hypothesis^63,64^. Another study in western obese subjects reported the decrease in levels of salivary *P. intermedia* upon the nutritional intervention of Mediterranean diet, indicating that a western diet-associated oral *P. intermedia* species decreased upon changing the diet^65^. However, one of the apparent limitations of this hypothesis is that most human gut and oral microbiome research have been performed on westernized populations, and similar knowledge is not available from non-western populations. Thus, it may remain worth examining if the mouth–gut axis observed in the western population can be extrapolated to non-western populations such as the Indian and African populations.

It was also noted that the richness, diversity, and distinct composition of virulence factors (VFs) in *Prevotella* genomes in western populations compared to non-western populations were sufficient to classify western and non-western populations with high accuracy. The number of antibiotic resistance genes (ARGs) were also significantly higher in western populations, and showed a similar segregation of western and non-western populations based on ARGs abundance. Studies have found the prevalence of ARGs in common isolates of *Prevotella* from the head and neck infection including *P. intermedia*, *P. melaninogenica*, *P. oris*, and *P. oralis* group, and these species were also among the differentially abundant *Prevotella* species in gut microbiome of western population^66,67^. Among the ARGs, those involved in the inactivation of antibiotics were the most prominent in *Prevotella* genomes followed by antibiotic efflux, antibiotic target alteration, and target protection. Notably, the ARGs belonging to the drug class of tetracycline antibiotics were the most abundant in *Prevotella* genomes, perhaps due to their frequent usage in treating periodontal diseases, and *P. intermedia* isolates resistant to tetracycline and its doxycycline and minocycline derivatives have also been reported^29,66,68^.

Among the different species in the *Prevotella* genus, *P. copri* has been gaining the status of a beneficial gut commensal due to its positive association with glucose homeostasis and cardiometabolic markers, and negative association with visceral fat, fasting VLDL-D, and fasting GlycA. Further, the individuals with *P. copri* showed lower C-peptide, insulin, and TG levels compared to *P. copri*-negative individuals^69,70^. Roles of *P. copri* in glucose homeostasis^71,72^ and in the metabolism of high carbohydrate and fiber-rich diet explain the intriguingly high abundance of this species in the gut microbiome of healthy Indian and non-western population that consumes a plant-associated carbohydrate and fiber-rich ingredients as the major component in the diet.

The CAZy analysis provided unique insights on the contributions of *P. copri* in the metabolism of complex polysaccharides in non-western populations. Plant-based diet includes dietary polysaccharides containing alpha (α)- and beta (β)-glycosidic bonds, and dietary fibers comprising of insoluble and soluble carbohydrates, including cellulose, lignin, and non-starch polysaccharides such as hemicelluloses, pectin, and arabinoxylan^32,73,74^. The human genome encodes enzymes that readily hydrolyze α-1,4-bonds but depends upon the ability of intestinal bacteria such as *P. copri* to break down complex plant polysaccharides with β-linkages^32,75–77^. Further, the presence of multiple strains of *P. copri* can catabolize a greater diversity of polysaccharides than any individual strain^11,32^. We also identified multiple *P. copri* clades with differential representation and extensive repertoire of carbohydrate-active enzymes (CAZy) families with a higher number of significant correlations in non-western populations, which highlights the importance of the presence of multiple *P. copri* strains and their role in carbohydrate metabolism in non-western populations. Majority of the differentially abundant glycosyl hydrolases (GHs) in the Indian population were falling under the plant carbohydrate source utilizing group described by Kaoutari et al.^43^ and Smits et al.^33^, indicating their role in Pectin/Hemicellulose and starch metabolism. Interestingly, the samples from Indian and Tanzanian populations showed high relatedness in *Prevotella* genome and gene composition, and also in carbohydrate metabolism potential (CAZy familes). These observations intrigued us to examine the similarities in the diets in the two populations, which were found to be similar and included cereals, pulses, vegetables, and fruits as the major ingredients^33^.

One of the key findings of the study is the identification of PULs containing pullulanase (GH13_14) and other CAZy families including GH77, GH97, GH13, GH43_4, GH43_5, and GH51, which provide evidence for the presence of complex starch and non-starch plant-polysaccharide metabolizing enzymes including some hypothetical genes in same genomic loci in *P. copri* genomes. GH13_14 subfamily comprises pullulanases, a very potent enzyme that catalyzes the hydrolysis of α-1,6-linked branches in glycogen, amylopectin, and other starch-derived glucans, as well as pullulan^78^. The presence of several hypothetical genes in this locus also hints towards the role of these genes in starch and non-starch metabolism^22^. These findings corroborate with the role of *P. copri* species in the comprehensive metabolism of complex plant-based polysaccharides in the Indian and other non-western populations.

Utilization of xylan found in cereal grains has been repeatedly established in *Prevotella* species and specifically for *P. copri*^3,8,79^. The numerous xylan-degrading enzymes identified among PULs in *P. copri* isolates suggested that it might have an expanded xylan-degrading enzyme repertoire and possibly possess a superior ability to target xylan in comparison to the other intestinal bacteria. Here, an interesting speculation could be the association of abundance of *Prevotella* with the consumption of cereals particularly whole-grain wheat, which is a major constituent of the Indian diet. Several studies have also reported the increase in abundance of *Prevotella* with the supplementation of wheat bran arabinoxylan oligosaccharides (AXOS)^80–82^. Thus, it appears that the presence of novel species/strains of *P. copri* in non-western populations provides it with an enhanced capacity to metabolize complex carbohydrates and dietary fibers, which plays a key role in its selection and dominance in the gut microbiome, and its function in host metabolism and health.

In summary, the gut microbiome analysis of the largest cohort of healthy samples of a previously unexplored Indian population and its comparisons with non-western and western populations have provided new insights into the yet understudied *Prevotella* genus. The study revealed the highest abundance of *Prevotella* in the Indian population, its relatedness with non-western populations, and also revealed that the majority of *Prevotella* species are constituted by *P. copri* in non-western populations. The identification of pullulanase-containing PULs and clusters of complex plant-polysaccharide metabolism genes in *P. copri* clades also suggests the role of this species in complex polysaccharide metabolism in the gut microbiome of non-western populations. While the *Prevotella* species in non-western populations were majorly constituted by *P. copri*, the *Prevotella* species in western-healthy and IBD populations were more diverse and enriched in known inflammatory *Prevotella* species of oral origin, which makes it tempting to speculate that perhaps the mouth–gut axis is behind the notorious association of *Prevotella* with inflammations in these populations.

## Methods

### Indian data description

The study cohort consisted of 200 healthy samples belonging to different locations and age groups. Samples were collected from six different locations to capture the maximum diversity in the gut metagenome of the Indian sub-population, including Madhya Pradesh (central), Delhi-NCR (north), Rajasthan and Maharashtra (west), Bihar (east), and Kerala (south). The samples include 104 male and 96 female, age between 0.5 and 85 years, BMI of 21.12 ± 5.32 (mean ± SD). Among the 200 samples, 93 samples were collected from the central region (44 male and 49 female, age between 0.5 and 71 years, BMI of 20.16 ± 4.25 (mean ± SD)), 20 samples from the eastern region (11 male and 9 female, age between 13 and 66 years, BMI of 23.41 ± 3.99 (mean ± SD)), 57 samples from the southern region (29 male and 28 female, age between 3.5 and 60 years, BMI of 20.14 ± 6.13 (mean ± SD)), 16 samples were from the western region (10 male and 6 female, age between 3 and 85 years, BMI of 24.82 ± 6.66 (mean ± SD)), and 14 samples from northern region (10 male and 4 females, age between 19 and 76 years, BMI of 23.82 ± 4.00). A fraction of samples (phase-1)^2,18^ were used for the initial study that provided clues on the role of dietary habits, and higher prevalence and abundance of *Prevotella copri* in Indian subjects in shaping the Indian gut microbiome. For this study to examine the larger question on the role and impact of such intriguingly high abundance of *P. copri* in the Indian population, we have used all the sequence data from the collected 200 samples from both phase-1 (116 samples)^2,18^ and phase-2 (84 samples) to gain comprehensive into the Indian gut microbiome.

The fecal samples were collected, and their detailed information is provided in Supplementary Data 14 (metadata section). This study was approved by the Institute Ethics Committee (IEC) of the Indian Institute of Science Education and Research (IISER), Bhopal, India, and the recruitment of individuals and sample collection were carried out in accordance with IEC approved study. All samples were frozen within 30 min of collection and transported to lab within 48 h at 4 °C. After receipt, the samples were immediately stored at −80 °C refrigerator until further processing. Each participant filled out a consent form prior to sample collection, mentioning their age, location, gender, and dietary habits. The recruited participants did not undergo antibiotic treatment for at least 1 month prior to sample collection. The collected samples were taken forward for whole metagenome sequencing.

### Fecal metagenomic DNA extraction and sequencing

From all the fecal samples, the metagenomic DNA was extracted using QIAamp Stool Mini Kit (Qiagen, United States) and following the manufacturer’s instructions except the final elution which was done in 50 µl of Elution buffer (Qiagen, United States)^83^. The extracted metagenomic DNA was quantified on Qubit 2.0 Fluorometer using Qubit dsDNA HS assay kit (Invitrogen, Life Technologies, United States). Until sequencing, all the DNA samples were stored at −80 °C.

The metagenomic DNA libraries were prepared by using the Illumina Nextera XT DNA library preparation kit (Illumina Inc., USA) and following the manufacturer’s reference guide. The size of libraries was evaluated on Agilent 2100 Bioanalyzer using a High Sensitivity DNA kit (Agilent Technologies, Santa Clara, CA). The libraries were quantified on Qubit 2.0 fluorometer using Qubit dsDNA HS assay kit (Invitrogen, Life Technologies, CA). Further quantification was done by qPCR following the Illumina suggested protocol which recommends the use of KAPA SYBR FAST qPCR Master mix and Illumina standards and primer premix (KAPA Biosystems, Wilmington, MA). The quantified libraries were normalized, pooled, and taken forward for 150 bp paired-end sequencing using NextSeq 500/550 v2 sequencing kit on Illumina NextSeq 500 platform (Illumina Inc., USA) at Next-Generation Sequencing (NGS) Facility, IISER Bhopal, India.

### *Prevotella copri* isolates

For the isolation of *P. copri* strains, fecal samples were collected from healthy human donors who did not have a history of any gastrointestinal disorders, enteric infections or exposure to antibiotics in the previous 6 months. Donor recruitment and fecal sample collection were performed after obtaining approval from the South Dakota State University Institutional Review Board. All donors signed informed consent. Fecal samples were processed, and strains were cultured^84^. *Prevotella*-positive isolates were grown on BHI agar plates, and mature colonies were collected for genomic DNA isolation with the PowerSoil DNA isolation kit (Qiagen). Libraries were prepared for sequencing on the MiSeq platform with the Nexetera XT DNA PCR-free Library Prep Kit (Illumina). In total five *P. copri* isolates were sequenced (Genome data is publicly available under Bioproject IDs PRJNA561792 (BioSamples: SAMN12628462, SAMN12628461, SAMN12628460, SAMN12628459) and PRJNA714938).

### Collection of publicly available metagenomic data sets from other population studies

The widely used and cited representative data sets from various western populations were selected using the below-mentioned inclusion/exclusion criteria for comprehensive analysis. We included subsets of widely known gut-microbiome cohorts of western populations like lifelineDeep (Netherlands)^34,85^, NLIBD (Netherlands)^34,85^, PRISM (US)^34^, MetaHIT (the US and European)^19^, etc. The considered inclusion criteria include the availability of metadata, comparable proportion of both genders and spanning a wide range of age groups to exclude the effect of these covariates in the analysis, sequencing of samples using the Illumina sequencing platform, cross-section studies of cohorts to incorporate maximum diversity, and IBD cohorts with representation from both ulcerative colitis and Crohn’s disease. The geographical classification of regions in western and non-western is discussed in Supplementary Note 1^35,86–88^. Healthy samples include both western and non-western data sets. Among non-western data sets, 112 samples (58 male and 54 females, age between 16 and 72 years, BMI: 21.37 ± 2.18) from Madagascar (Study accession: PRJNA485056)^10,12^, 67 samples from Tanzania (Study accession: PRJNA392180 (single-end reads), PRJNA278393 (paired-end reads) (33 males and 21 females, age between 4 and 70 years))^14,33^ and 36 samples from Peru (13 males and 22 females, age between 1 and 52 years, BMI: 20.55 ± 4.55)^13^ were included for analysis. Among western-healthy samples, 101 samples from Italy (50 male and 51 females, age between 21 and 64, BMI: 22.51 ± 3.34)^9^, 34 samples US (age between 22- and 82-years), 22 samples from Netherlands (age between 22- and 82-years LLDeep data set)^34^ and 14 samples from Spain (age between 18 and 68 years)^19^.

IBD data sets include 121 samples from the US (68 CD samples with age between 21 and 77 years and 53 UC samples with age between 20 and 76 years), 43 samples from the Netherlands (20 CD samples with age between 21 and 71 years and 23 UC samples with age between 19 and 80 years)^34^ and 25 samples from Spain (4 CD with age between 21 and 41 years and 21 UC samples with age between 25 and 68 years)^19^.

### Pre-processing of the metagenomic reads

A total of 379.36 Gbp of metagenomic sequence data (mean 1.9 Gbp ± 2.03) was generated from 200 fecal samples from the Indian population. The metagenomic reads were filtered using the Trimmomatic (version: 0.39)^89^ with criteria of removing NexteraPE-PE.fa adapters and seed mismatch value of 2 and maximum quality value 30 for paired-end reads and 10 for single-end reads. Removed leading and trailing sequences having less than or equal to the quality value of 25. The high-quality reads were further filtered to remove the host-origin reads using bmtagger v.3.101 (human contamination)^90^, which resulted in the removal of an average of 0.3% of reads (Supplementary Data 14).

### Assembly and binning of metagenomic data

Each of the 775 samples were processed with the standard quality control and then independently subjected to de-novo metagenomic assembly through metaSPAdes (version 3.13.0; default parameters)^10,91^. Samples that failed to be processed due to memory requirements (>1Tb of RAM), and samples with only unpaired reads, were assembled through MEGAHIT^92^ (version 1.2.8; default parameters). Reads that are not represented among the contigs from paired-end read assembly were extracted using FR-HIT^93^ (v.0.7.1), concatenated with single-end reads, and assembled using MEGAHIT. A total of 10,455,670 contigs were generated after assembly and exclusion of contigs shorter than 1000 bps (Supplementary Data 15).

We performed single-sample assembly and binning (rather than co-assembly) to preserve strain variation between human hosts, and because co-assembly was not computationally feasible for our large data set. For identifying which binning method works better for our data sets, we have binned all 1,481,535 contigs from 200 Indian samples using metaWRAP^94^ (v1.2.3) pipeline using the coverage information of each contig in the 44 samples from India that sequenced recently. Binning with Metabat2^95^ produced the highest number of high-quality bins (10 bins with completeness >90 and contamination <10) and this method was selected for binning other samples. CheckM^96^ (1.1.2) was used to quantify the quality of bins produced.

A total of 10,455,670 contigs from 8 healthy and 3 IBD data sets were considered for binning. Reads were mapped to contigs using Bowtie2^97^ (v2.3.5.1; option ‘--very-sensitive-local’), and the mapping output was used for contig binning through MetaBAT2^95^ (version 2.12.1; option ‘-m 1500’), and initial bins were subjected to quality control to generate the final set of reconstructed draft genomes (Supplementary Fig. 24). The ‘merge’ bin option provided CheckM^96^ was used to identify pairs of bins where the completeness increased up to ≥90% and the contamination ≤10% when merged into a single bin. The ‘taxon_set’ option in CheckM was used to produce marker sets for the *Prevotella* genus and passed it to the analyze option in order to identify marker genes within each genome/bin and estimate completeness and contamination. Now we have all the bins with the aforementioned criteria of completeness and contamination based on *Prevotella*-specific marker gene sets and were named as Selected Bins (SB) in the further text (Supplementary Data 3).

### Bin refinement strategies

Bin refinement has been carried out using three strategies; alignment-based, genomic properties based, and taxonomic annotation-based. To be more inclusive, refinement of Contaminated Bins (CBs: the bins that are ≥ 90% complete and >10% contamination) was carried out by flagging contamination on the basis of alignment of contigs between conspecific genomes^98^ (see Supplementary Note 13). Further 20,000 contigs distributed into 164 bins (112 SBs + 52 Refined CBs) were subjected to identification of potential contamination based on the genomic properties (GC, tetranucleotide signatures, coverage) of contigs using RefineM (v0.0.25) (https://github.com/dparks1134/RefineM). Next level of bin refinement was carried out on the basis of taxonomic annotation of all contigs in each bin using CAT^99^. Contigs classified till *Prevotellaceae* family from each bin were retained and the other contigs were removed. Bins having completeness ≥90 and contamination <10 were selected for further analysis (see Supplementary Note 13).

### *Prevotella* genome database construction and calculation of genome abundance

1,612 reconstructed genomes assigned to *Prevotella* genus (out of 154,723) having ≥90% completeness and <5% contamination were retrieved from  https://opendata.lifebit.ai/table/SGB^10^. Taxonomic assignment of 1612 HQ bins was carried out using CAT/BAT for further confirmation. All genomes/bins were assigned till *Prevotellaceae* family with support value per rank >0.70 and 1610 genomes were assigned till *Prevotella* genus with support value per rank >0.70 (Supplementary Data 4). In addition to these 1612 reconstructed *Prevotella* genomes, the *Prevotella* genome database includes 547 reference genomes downloaded from NCBI, 15 *Prevotella* isolates from the previous study, 5 isolates from our study and 25 final HQ bins. A total of 2204 genome/bins were contained within the *Prevotella* genome database. We calculated pairwise distances for 2204 genomes/bins using Mash v2.1 (default sketch size)^100^. The relative abundance of each genomes/bins in each sample were quantified using the quant_bins option in Meta-WRAP and the genomes having relative abundance per sample ≥0.001% were considered for genome/bin composition analysis. ‘Labdsv’ package^101^ was deployed to detect significantly discriminating *Prevotella* genomes in western and non-western populations. Discriminating *Prevotella* genomes with indval score >0.60 (*p*-value < 0.01) were considered for further analysis. A dendrogram of all 102 differentially abundant genomes/bins was also constructed using ‘aheatmap’ function (distfun = “spearman”) of NMF package^102^ in R. Prediction of Polysaccharide Utilization Loci (PULs) from 2204 genomes/bins in the *Prevotella* genome database was carried out using PULpy^44^ (https://github.com/WatsonLab/PULpy). The presence of operon genes among PULs was identified using Operon-mapper^103^.

### Taxonomic annotation of reads and contigs

Taxonomic assignment of reads was carried out using Kaiju^104^, a program in which reads are directly assigned to taxa using the NCBI taxonomy and a reference database of protein sequences from microbial and viral genomes. The database used for the analysis is a subset of NCBI BLAST nr database containing all proteins belonging to Archaea, Bacteria, and Viruses. Percentage of reads assigned to each genus was calculated (Supplementary Data 1). Contigs >1000 bp from each data set were classified into taxonomic clades using CAT^99^ with aforementioned database and parameters. Percentage of contigs assigned to *Prevotella* genus as well as *Prevotella copri* was extracted (Supplementary Data 1).

### *Prevotella copri* clade composition analysis

1021 reconstructed bins of *P. copri* were also retrieved from Tett et al.^11^ and estimated the bin abundance across samples using the quant_bins option in Meta-WRAP^94^. Representations of each clade in each population were evaluated by calculating the number of genomes present in each population out of the total number of genomes in each clade. To check the distribution of genomes from each clade, we calculated the number of genomes from each clade in each population based on different criteria that the genomes should present in at least one sample, more than 10% of the samples, more than 50% of the samples and more than 70% of the samples of each population.

Contigs >1000 bp (1,481,535 contigs) from Indian population were classified using CAT/BAT and 429,703 contigs were assigned to *Prevotella* genus. Out of 200 Indian samples, 116 samples had >10% abundance of *P. copri*, as estimated by Kaiju analysis, and were selected for optimal mapping of reads as per the strategy suggested by Pasolli et al.^10^. The reads from 116 samples were aligned against the *Prevotella* contigs to estimate the coverage of each contig. *Prevotella* bins were constructed using contig coverage and tetranucleotide frequency, and a total of 42 bins with completeness >50 and contamination <10 were identified (Supplementary Fig. 13a and Supplementary Data 9). 72 high-quality metagenomes assembled manually curated *P. copri* genomes were retrieved from Tett et. al.^11^, and a *P. copri* genome/bin set was constructed, including 72 high-quality *P. copri* genomes/bins, 42 bins constructed in this study, and 5 Indian *P.copri* isolates. For clade level assignment of 47 (42 bins+ 5 isolates) *P copri* genomes/bins, pairwise intergenomic distances of each genome/bin were calculated using MASH^100^.

### Construction of *Prevotella* gene catalog

All possible genes from genomes belonging to the *Prevotella* genus have been considered for this analysis. It includes (i) 547 reference genomes downloaded from NCBI, (ii) 1612 reconstructed genomes assigned to *Prevotella* genus (out of 154,723) having ≥90% completeness and <5% contamination retrieved from https://opendata.lifebit.ai/table/SGB, (iii) 15 *Prevotella* isolates from the previous study, (iv) 5 isolates from this study, (v) 25 final HQ bins, and (vi) Genes predicted from initial contigs >1000 bp that are assigned to *Prevotella* genus by CAT^99^. All available gene files of 547 genomes were downloaded from NCBI and for remaining genomes, gene prediction has been carried out using Prodigal v2.6.3^105^ (with -p option for gene prediction from initial *Prevotella* contigs), and total (Supplementary Data 10) 31,758,457 genes were used for the analysis. Redundant genes were removed using CD-HIT v4.8.143^106^ with (sequence identity threshold of 0.99). This resulted in a total of 2,992,963 genes (>100 bp) in the final PGC.

### Gene abundance calculations

High-quality reads were aligned to the PGC using BWA (v0.7.17)^107^, and the filtered read pairs were mapped to the same gene using the read_count_bam.pl script^19^, and the mapped read pairs with a mapping quality better than 30 (-q30 below) were considered. Gene counts from samples having paired-end, as well as single-end reads, were added to construct the final gene count table. Rarefaction has been carried out using GUniFrac R package^108^ using a value of depth as 0.1 million and 0.5 million. Principal coordinates analysis showed that rarefaction depth is not affecting the microbial gene composition analysis. A rarefied gene proportion table with depth = 0.1 million was considered for further analysis. 7030 genes having cumulative proportion ≥0.01 were used for beta-diversity analysis. Functional annotation was performed for these 2.9 million genes present by protein alignment using DIAMOND^109^ against KEGG^110^ and CAZy^42^ databases. At the functional level, 2305 KEGG orthologues and 9332 CAZy orthologous groups were identified in the PGC.

### Identification of virulence factors

The virulence factor database (VFDB) is an integrated and comprehensive online resource for curating information about virulence factors of bacterial pathogens. Since its inception in 2004, VFDB has been dedicated to providing up-to-date knowledge of VFs from various medically significant bacterial pathogens^50^. Both protein sequences of core data set (http://www.mgc.ac.cn/VFs/Down/VFDB_setA_pro.fas.gz), as well as Protein sequences of full data set (http://www.mgc.ac.cn/VFs/Down/VFDB_setB_pro.fas.gz) were downloaded for this analysis. The core data set includes genes associated with experimentally verified VFs only, whereas the full data set covers all genes related to known and predicted VFs in the database. Protein homology search of genes in PGC against both core and full data set using DIAMOND and best hits having score ≥60 and *e*-value < 10^−^^6^ were considered for calculating VF gene abundance. Analysis using the core VF database identified 137 VF gene ids and they were mapped to 133 UniProt-ids. Analysis using a full VF database identified 254 VF gene ids and they were mapped into their corresponding 205 UniProt-ids. The total number of VFs identified in each population (present at least one sample) were calculated. Differentially abundant VFs in western and non-western were also identified.

### Identification of antibiotic resistance genes

Command line version of the Resistance Gene Identifier (RGI) was used to predict resistomes from protein or nucleotide data based on homology and SNP models^54^. Three different criteria (perfect, strict, and loose) based on different types of hits in homology search were involved in prediction. A “perfect” match is 100% identical to the reference sequence along its entire length. A “strict” prediction is a match above the bit-score of the curated BLASTP bit-score cut-off. “Loose” matches are other sequences with a match bit-score less than the curated BLASTP bit-score that helps in the detection of new, emergent threats and more distant homologs of Antimicrobial Resistance (AMR) genes, and in cataloging homologous sequences and partial hits that may not have a role in AMR. Both “strict” and “loose” criteria were used for the detection of ARGs in this population data sets. Analysis using loose criteria detected 1357 ARGs, whereas analysis using strict criteria detected 18 ARGs. The total number of ARGs identified in each population (present at least one sample) were calculated, and the average abundance of AMR genes were calculated from normalized gene abundance data.

### Statistical analysis

Rarefied Gene proportion (0.1 million depth), Genome proportion, KO proportion, carbohydrate metabolizing gene proportion, ARG proportion, and VF gene proportion were used for statistical analysis. Alpha-beta diversity and PERMANOVA (with permutations = 999) analyses was carried out using vegan^111^ and ape^112^ R-packages. Plots were generated using ggplot2^113^. indval function in the labdsv R-package was used for identification of genomes/bins, pathways carbohydrate metabolizing gene families differentially abundant in different groups of data under consideration. WEKA^114^ was used for randomforest analysis^115^. Boruta^116^, LEfSe^117^, and labdsv^101^ packages were utilized for finding differentially abundant taxa in populations. “CCREPE” package in R was used for correlation analysis and cytoscape^118^ was used for plotting co-occurrence plots using significant correlation values.

### Reporting summary

Further information on research design is available in the Nature Research Reporting Summary linked to this article.

## Supplementary information

Supplementary Information

Reporting Summary

## Data Availability

The metagenomic reads from sequencing of fecal samples are available under BioProject ID PRJNA397112 (study accession: SRP114847), PRJNA331073 (study accession: SRP079687), and PRJNA715908 (study accession: SRP311507). The five isolate *P. copri* genomes are deposited in the NCBI BioProject database under project numbers PRJNA561792 (BioSamples: SAMN12628462, SAMN12628461, SAMN12628460, SAMN12628459) and PRJNA714938. Supplementary Data files are uploaded in figshare (https://figshare.com/), and the DOI link to access the data is 10.6084/m9.figshare.16586951.
