# Supplementary material for: Western and non-western gut microbiomes reveal new roles of Prevotella in carbohydrate metabolism and mouth–gut axis
Source: NPJ Biofilms Microbiomes. 2021 Oct 7;7:77. doi: 10.1038/s41522-021-00248-x (PMC8497558; doi:10.1038/s41522-021-00248-x)
Supplement: Supplementary file 1 — Supplementary Information [file 41522_2021_248_MOESM1_ESM.pdf]

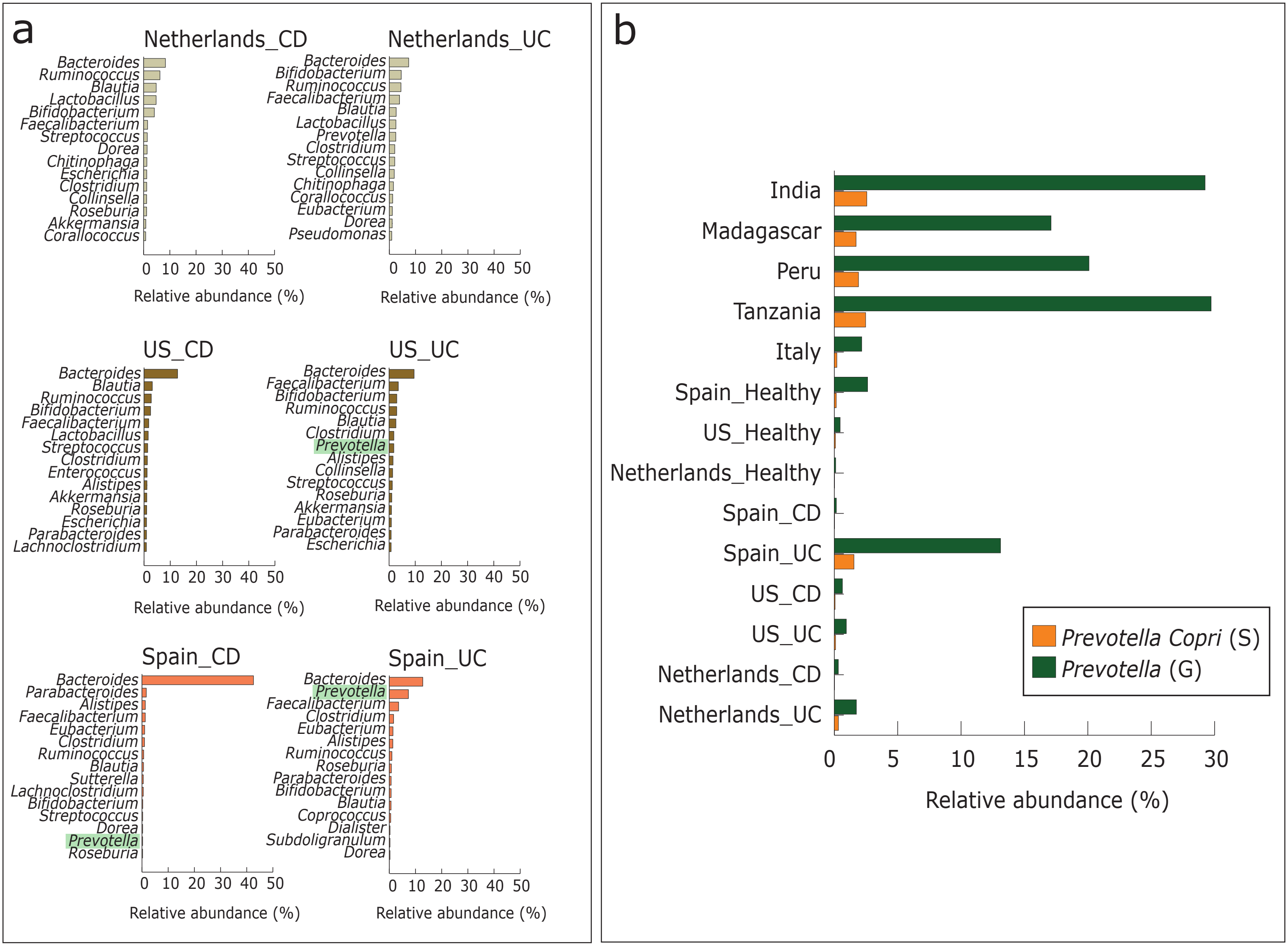

Supplementary Figure 1: Relative abundance of *Prevotella* genus in healthy and IBD populations
a) Relative abundance of top 15 genera in IBD populations based on taxonomic assignment of high-quality reads (ulcerative colitis (UC) and crohn's disease (CD) samples were represented separately).
b) Relative abundance of *Prevotella* genus and *P. copri* species in IBD populations based on taxonomic assignment of contigs (ulcerative colitis (UC) and crohn's disease (CD) samples were represented separately.

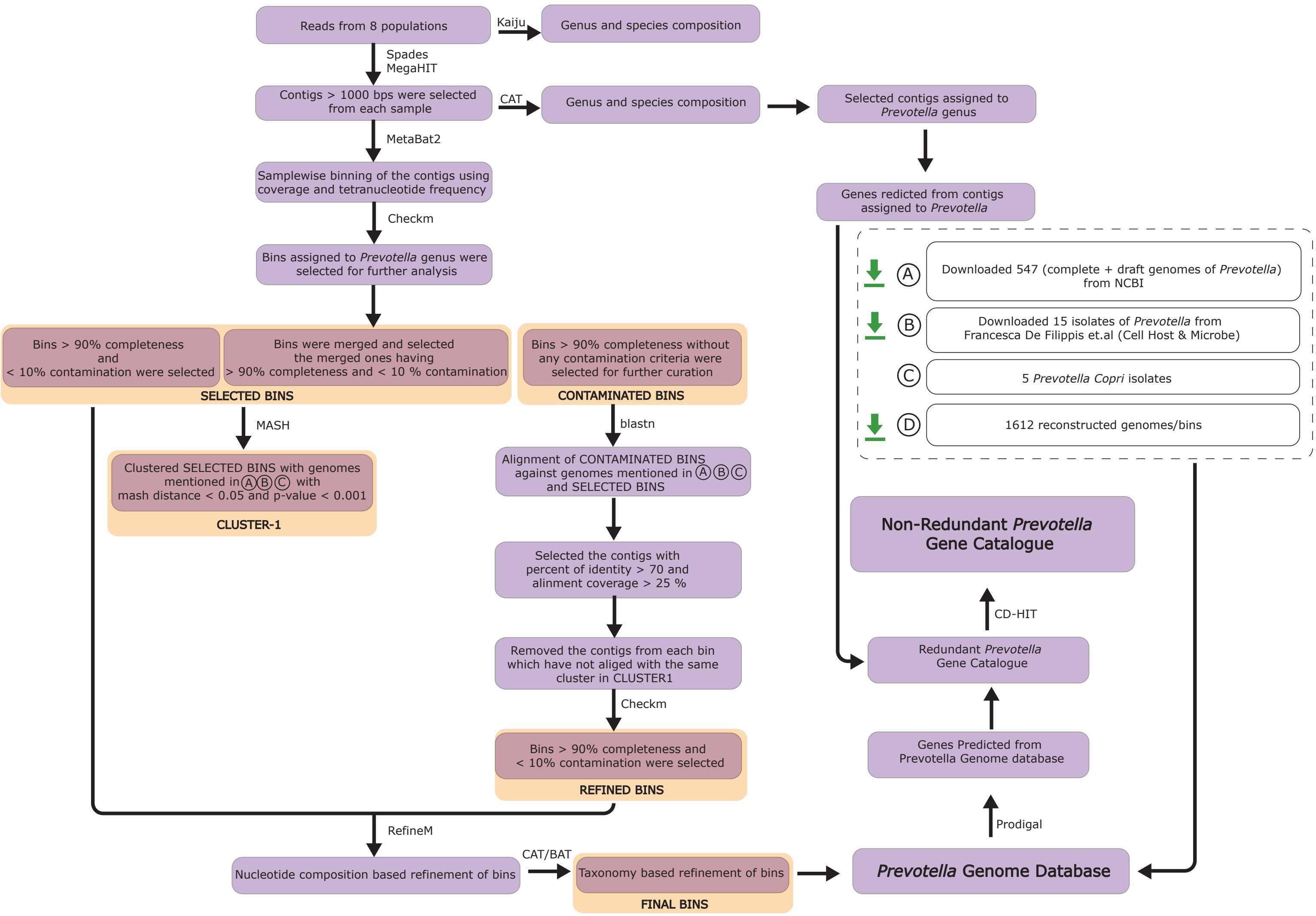

Supplementary Figure 2: Analysis pipeline of the study
Flowchart summarizes the analyses carried out in this study. It includes Metagenome assembled genomes (MAGs) reconstruction, *Prevotella* Genome Database (PGD) and *Prevotella* Gene Catalogue (PGC) construction and further analysis of the taxonomic and functional composition of *Prevotella*.

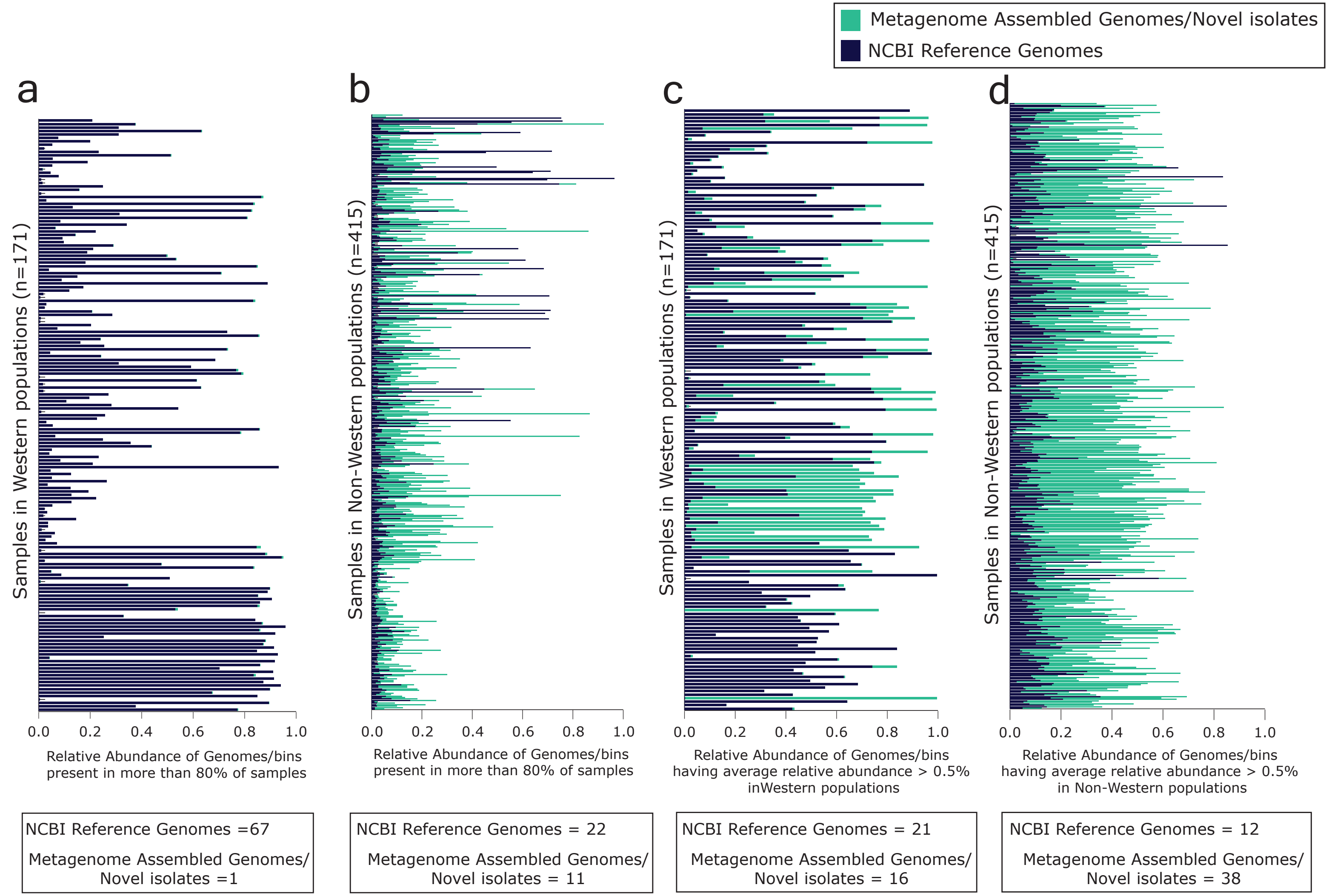

Supplementary Figure 3: Relative abundance of *Prevotella* genomes in western and non-western populations
a) Relative abundance of *Prevotella* genomes retrieved from NCBI database and MAGs present in more than 80% of samples in western populations.
b) Relative abundance of *Prevotella* genomes retrieved from NCBI database and MAGs present in more than 80% of samples in non-western populations.
c) Relative abundance of *Prevotella* genomes retrieved from NCBI database and MAGs having average relative abundance > 0.5 % in western populations.
d) Relative abundance of *Prevotella* genomes retrieved from NCBI database and MAGs having average relative abundance > 0.5 % in non-western populations.

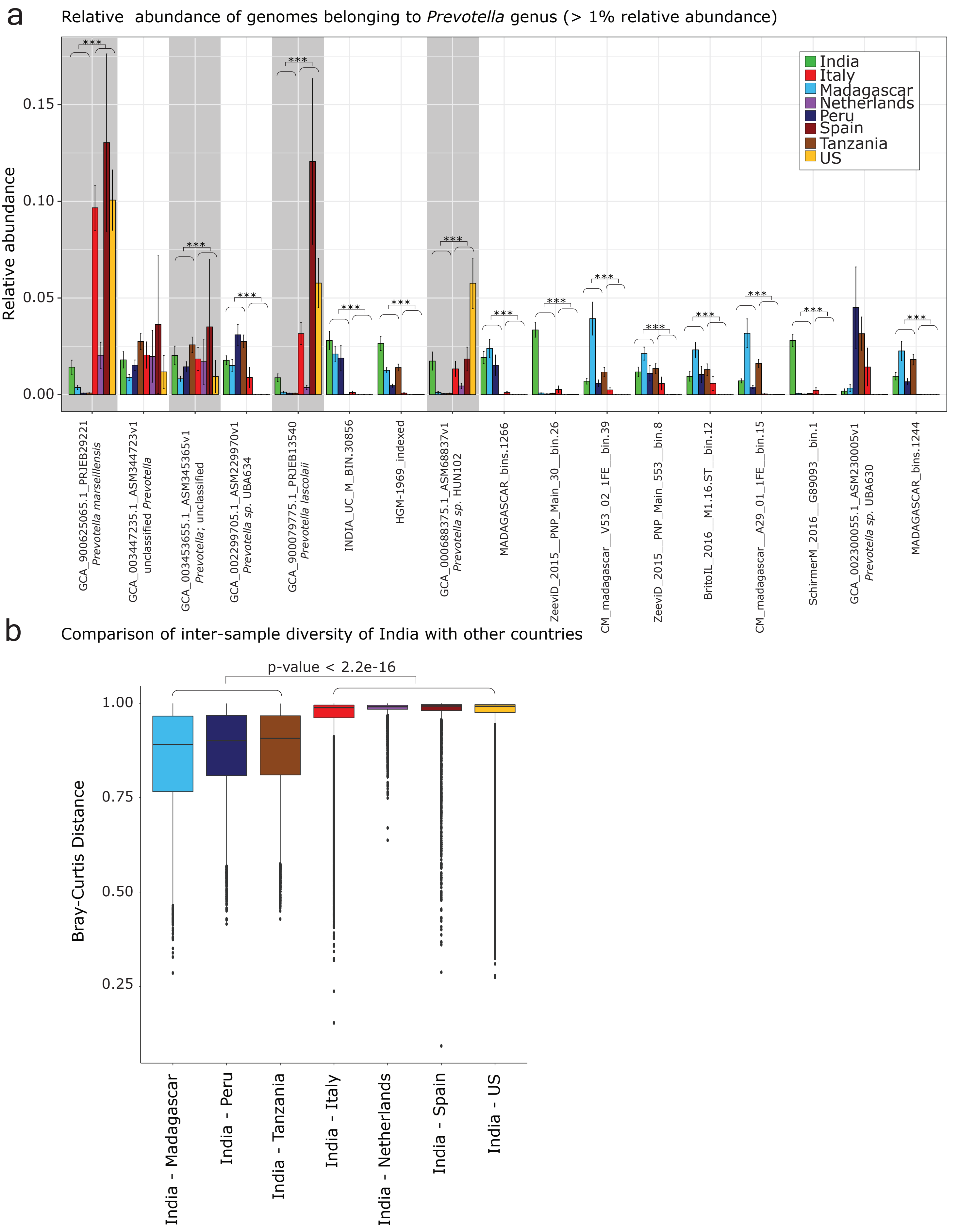

Supplementary Figure 4: Variation in *Prevotella* composition in healthy populations
a) Relative abundance of *Prevotella* genomes (with > 1% relative abundance cut-off) in healthy populations
b) Average inter-sample distance of Indian population based on *Prevotella* genome abundance with all other healthy populations
The line in the middle of the box, bound of the box and whiskers represent the median, 25th–75th percentiles, and min-to-max values, respectively. Nonparametric two-sided Wilcoxon rank sum test was used for testing the box plot distributions. ns, not significant; ***, p-value < 0.01.

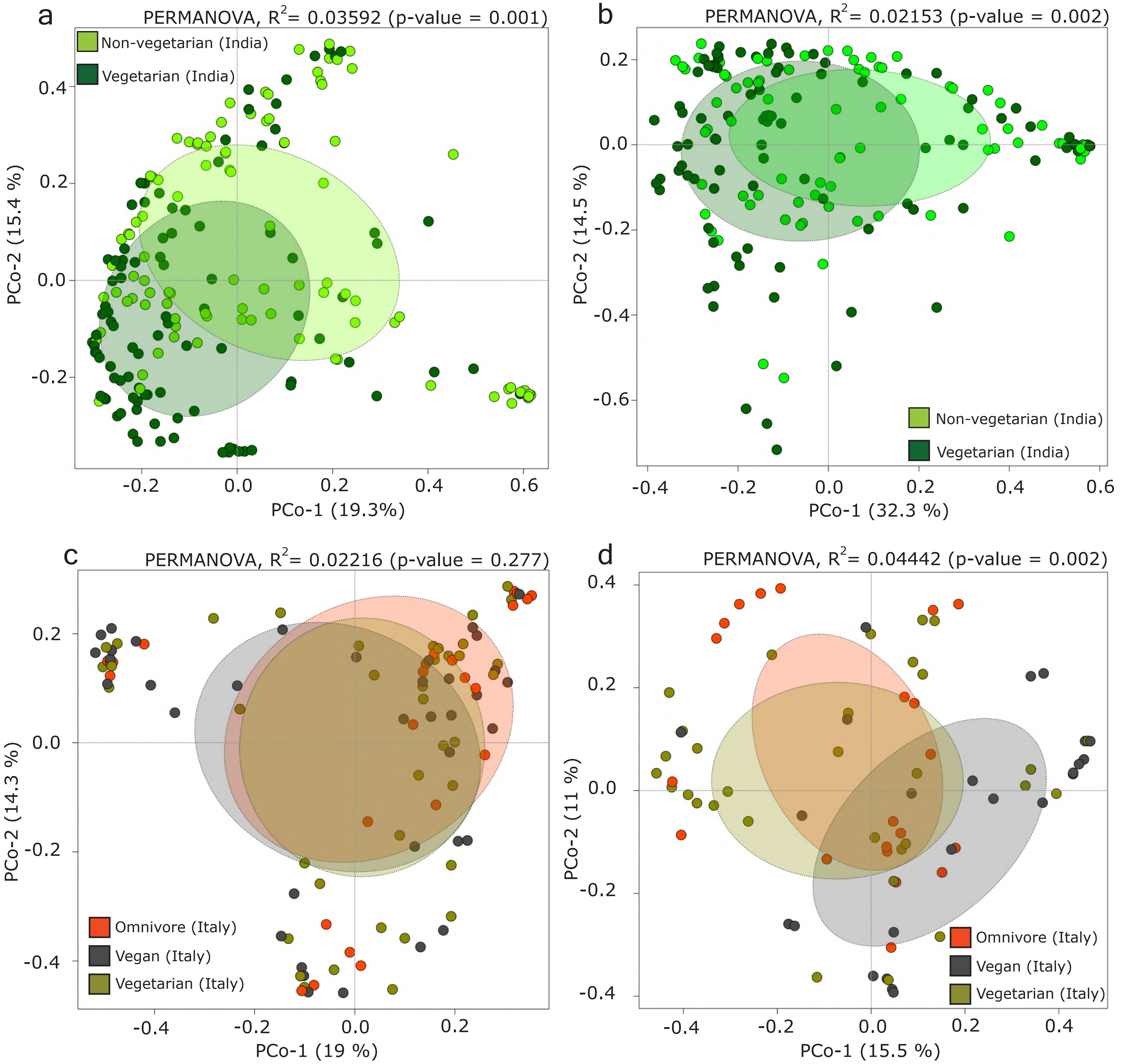

Supplementary Figure5: Effects of dietary habits and geographical locations in human gut *Prevotella* composition
a) Principal coordinates analysis considering inter-sample Bray-Curtis distance based on relative abundance of *Prevotella* genomes (in PGD) in samples of the Indian populations having vegetarian and non-vegetarian dietary habits.
b) Principal coordinates analysis considering inter-sample Bray-Curtis distance based on relative abundance of 1,021 *Prevotella copri* genomes in samples of the Indian populations having vegetarian and non-vegetarian dietary habits.
c) Principal coordinates analysis considering inter-sample Bray-Curtis distance based on relative abundance of *Prevotella* genomes (in PGD) in samples of the Italian populations having vegan, vegetarian and omnivorous dietary habits.
d) Principal coordinates analysis considering inter-sample Bray-Curtis distance based on relative abundance of 1,021 *Prevotella copri* genomes in samples of the Italian populations having vegan, vegetarian and omnivorous dietary habits.

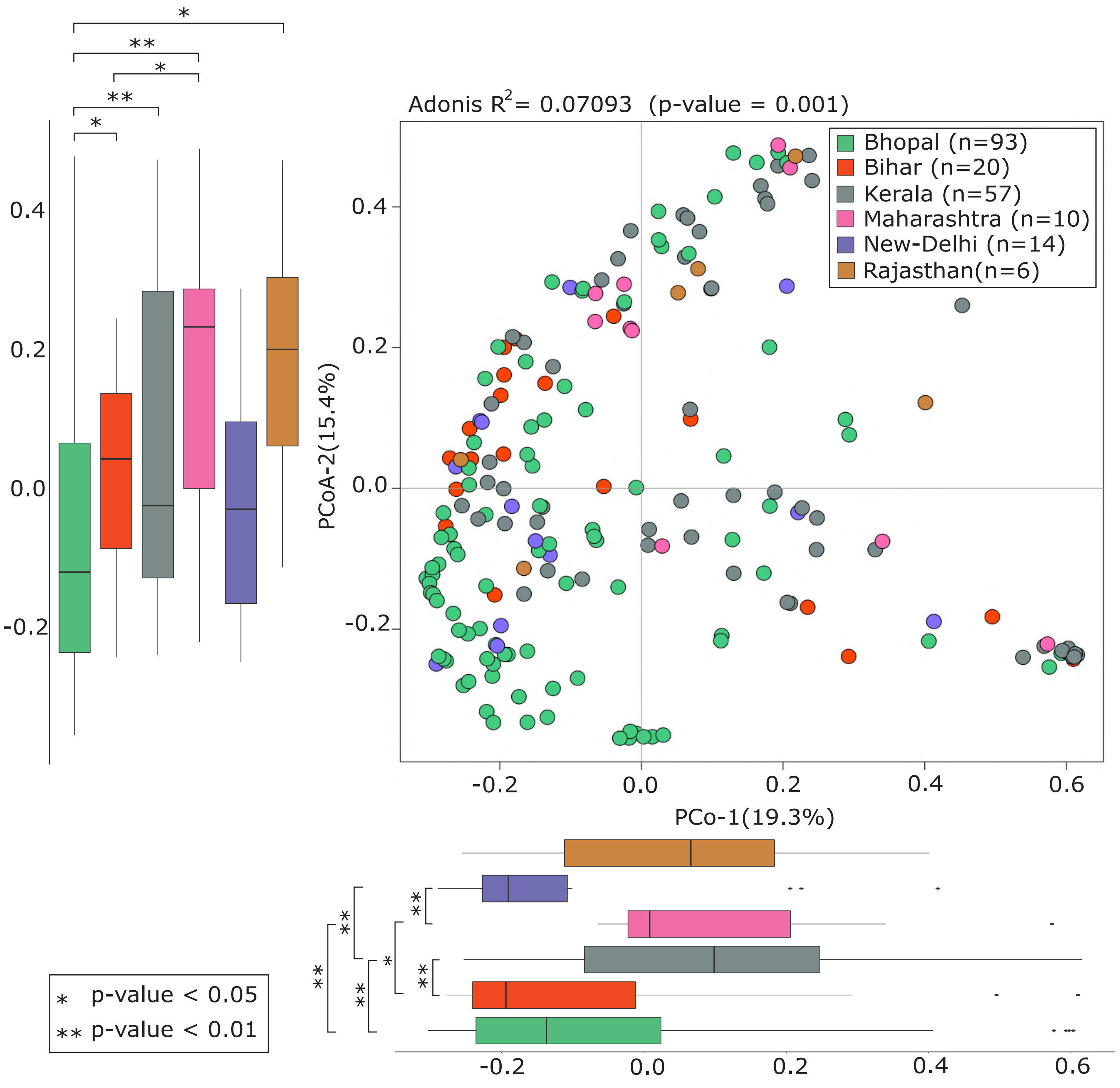

Supplementary Figure6: Principal coordinates analysis of Indian gut microbiome based on *Prevotella* genome abundance the samples (Bray-Curtis distance) from six different geographical regions. The line in the middle of the box, bound of the box and whiskers represent the median, 25th–75th percentiles, and min-to-max values, respectively. A nonparametric two-sided Wilcoxon rank sum test was used for testing the box plot distributions. ns, not significant; *, p-value < 0.05; **, p-value < 0.01.

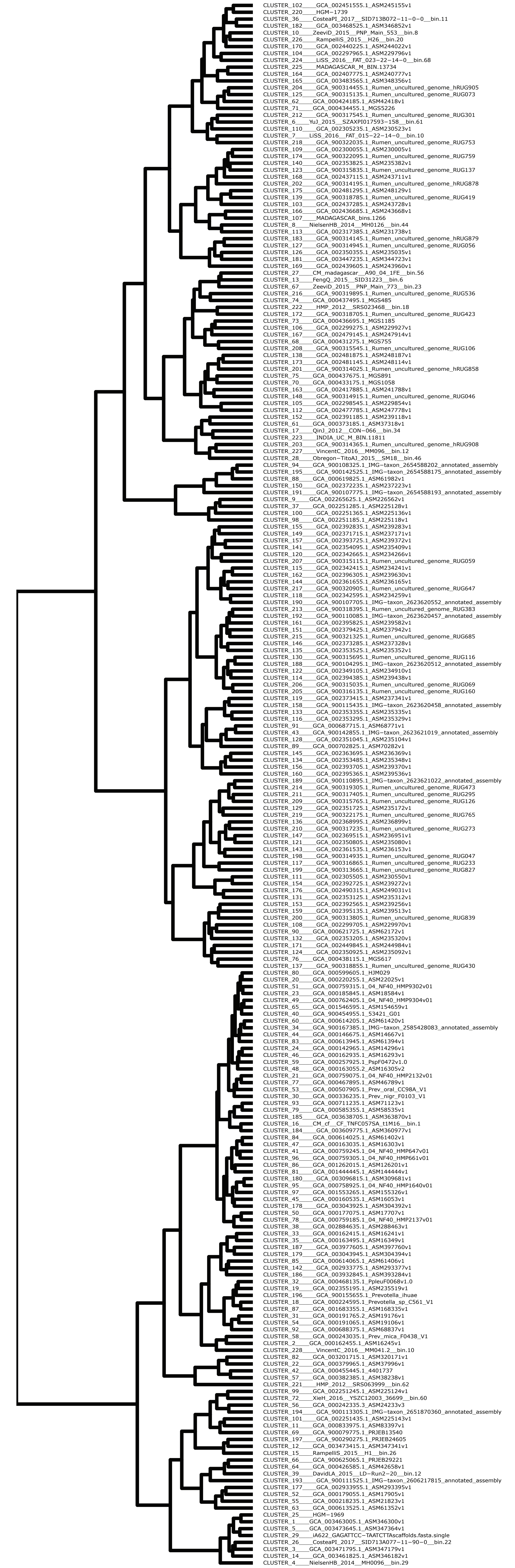

Supplementary Figure7: Cladogram constructed based on the inter-genome distance (MASH-distance, see Method section) between representative genomes/bins from 228 species-level clusters (MASH distance = 0.05 ~ 95% ANI)

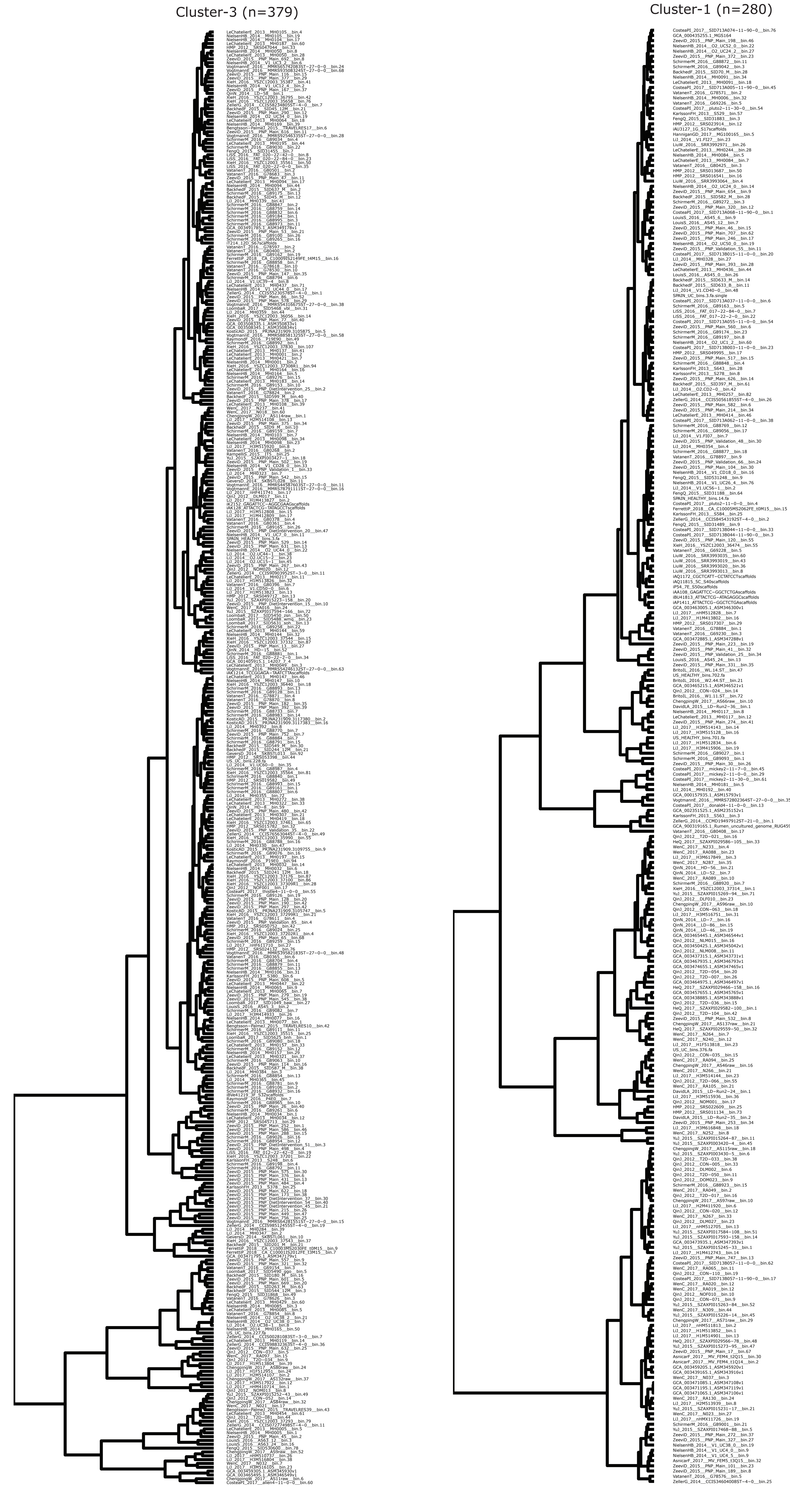

Supplementary Figure8: Cladogram of genomes/bins from two species-level clusters (>100 genomes/bins per cluster) constructed based on the inter-genome distance (MASH-distance, see Method section).

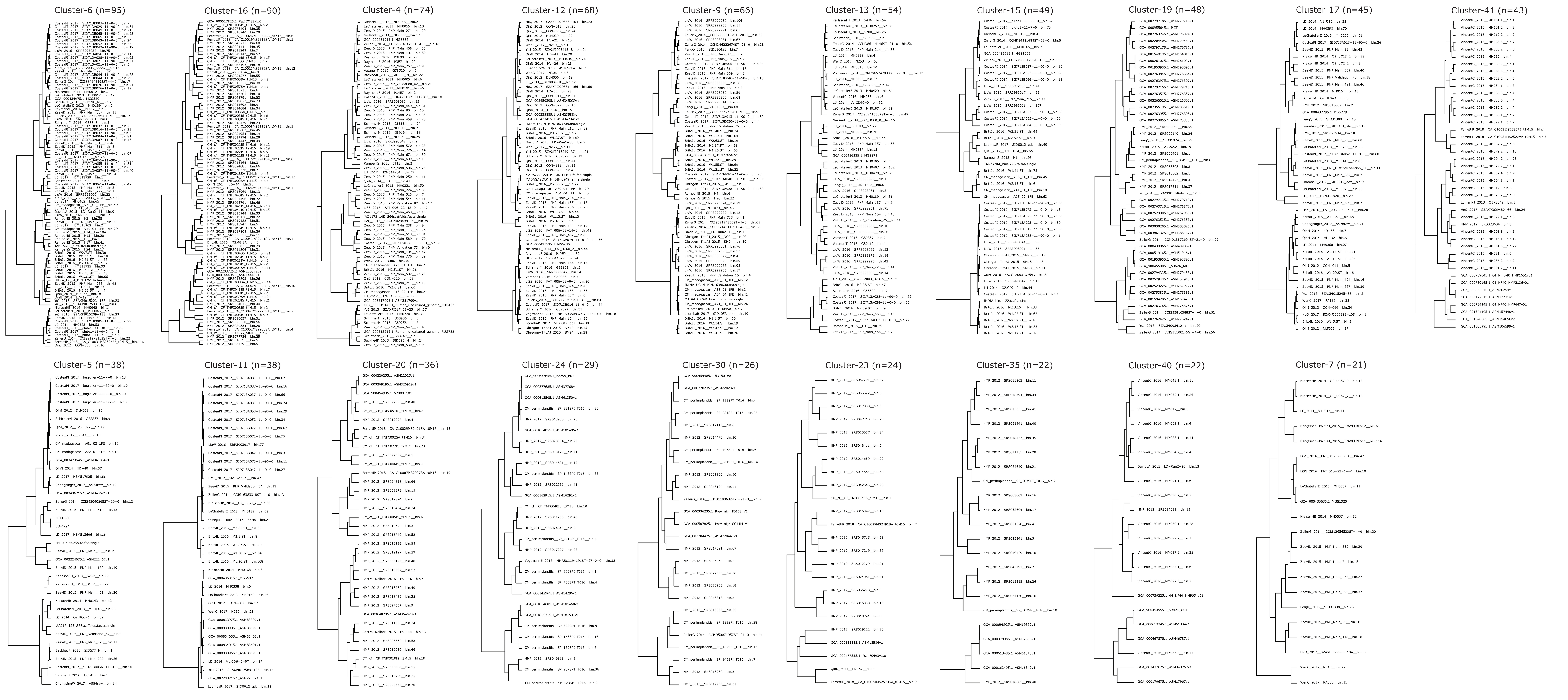

Supplementary Figure9: Cladogram of genomes/bins from two species-level clusters (<100 and >20 genomes/bins per cluster) constructed based on the inter-genome distance (MASH-distance, see Method section).

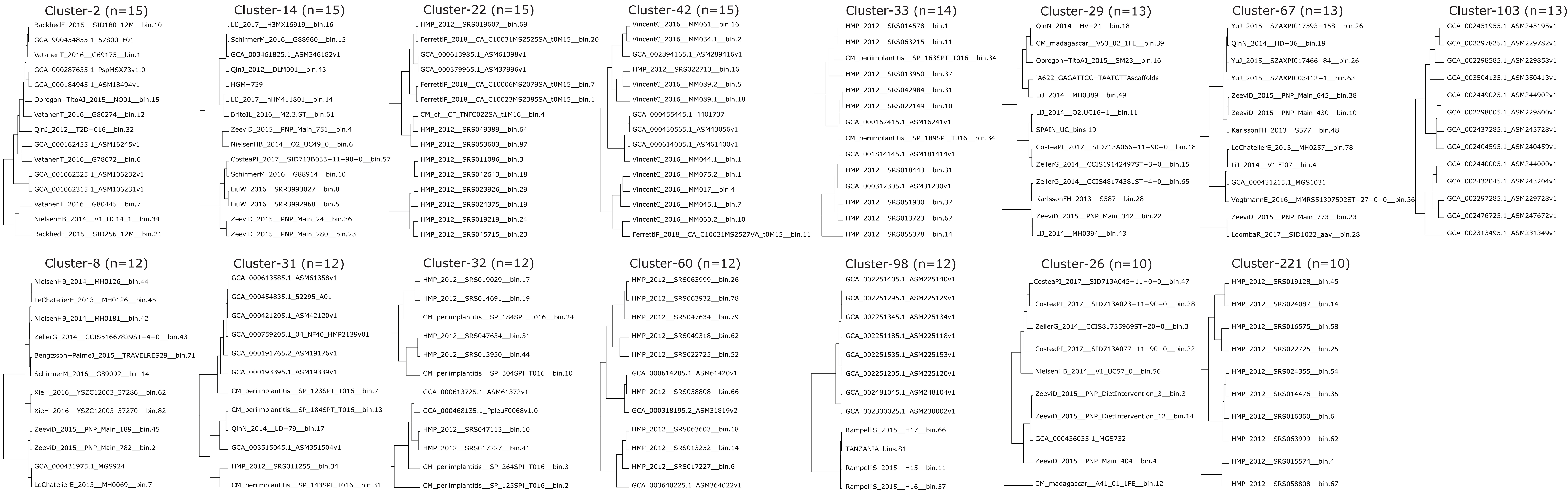

Supplementary Figure10: Cladogram of genomes/bins from two species-level clusters (<20 and >10 genomes/bins per cluster) constructed based on the inter-genome distance (MASH-distance, see Method section).

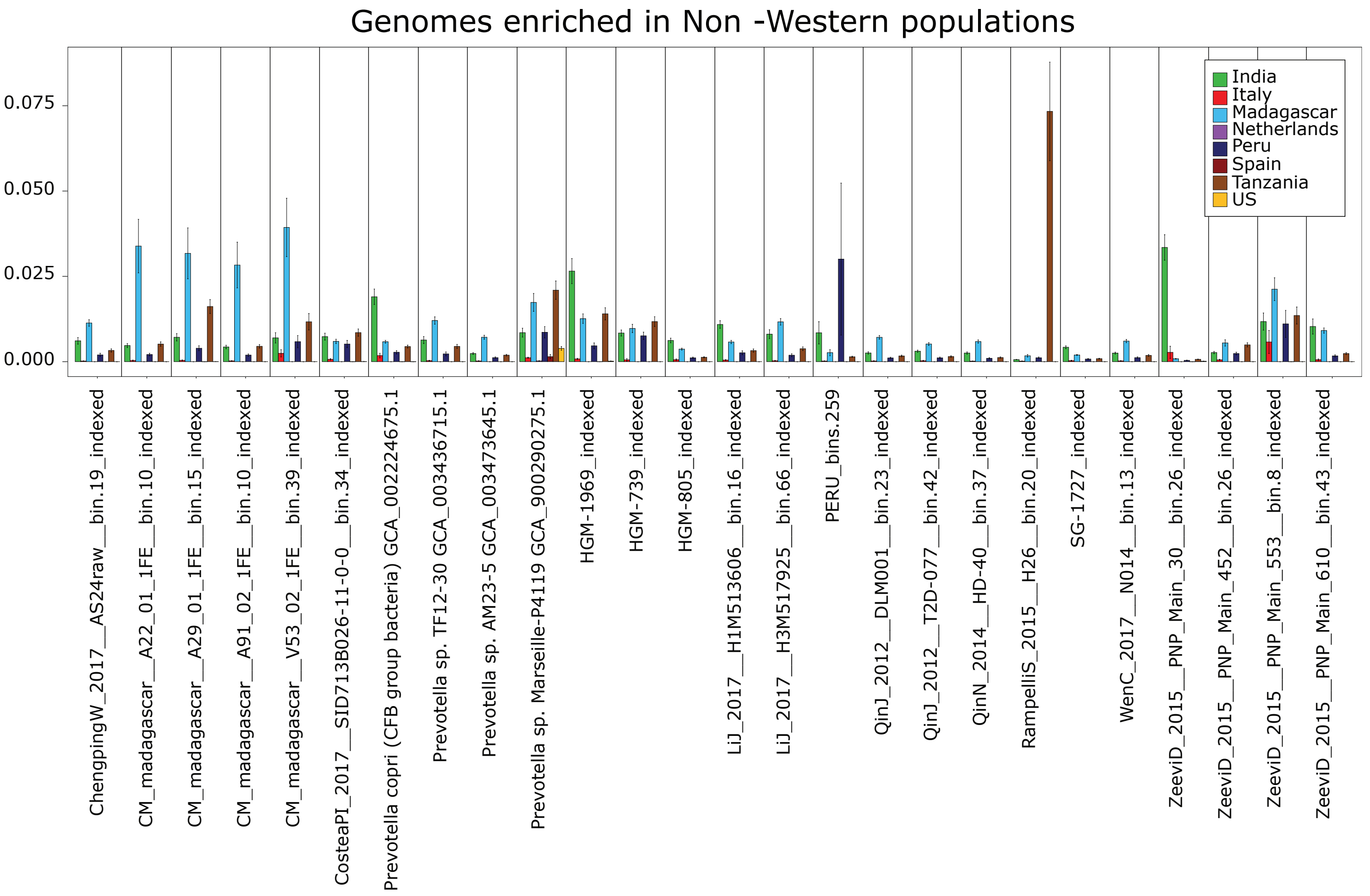

Supplementary Figure11: Relative abundance of 26 differentially abundant *Prevotella* genomes (calculated using labdsv package) in non-western populations.

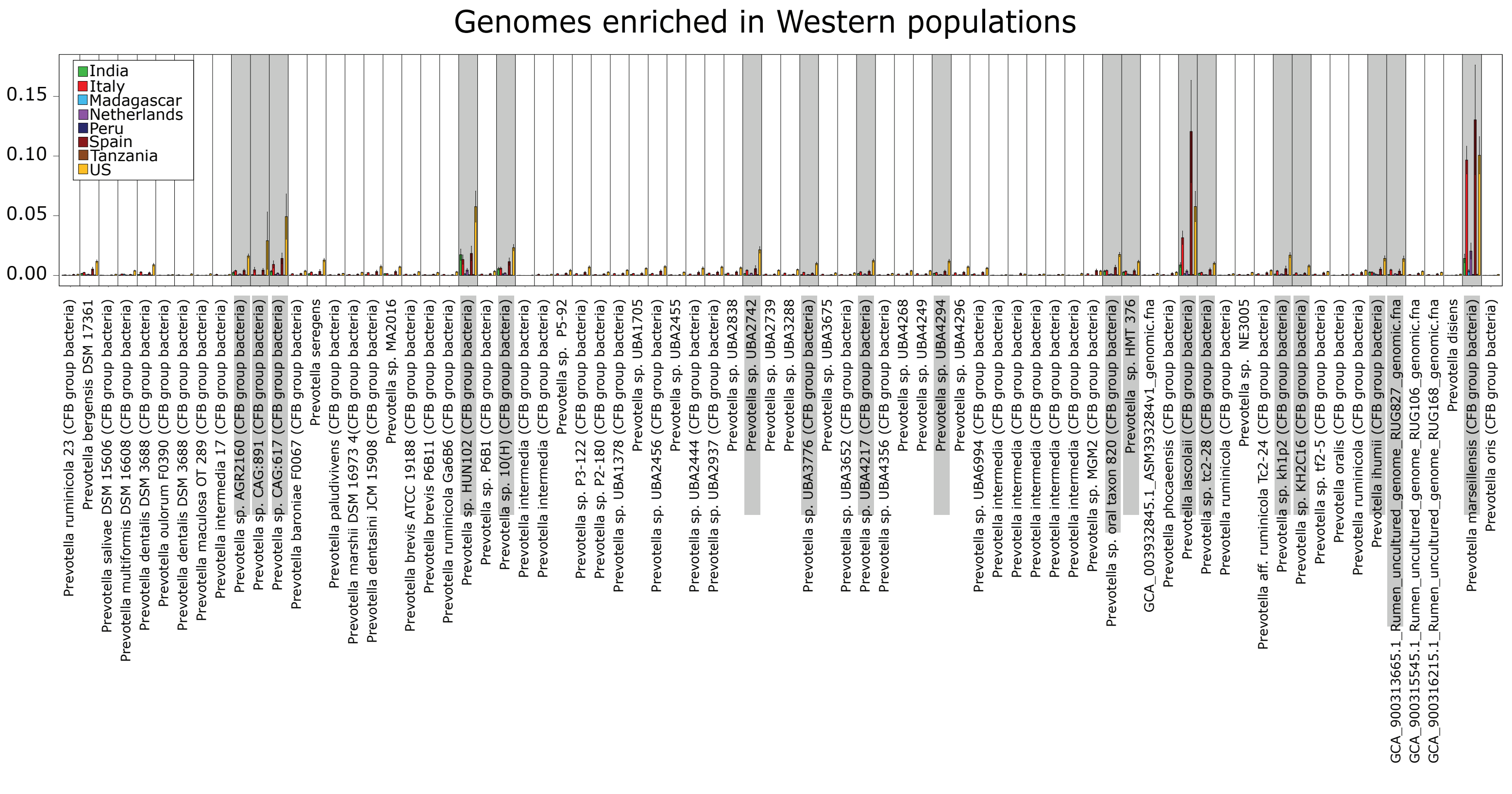

Supplementary Figure12: Relative abundance of 76 differentially abundant *Prevotella* genomes (calculated using labdsv package) in western populations.

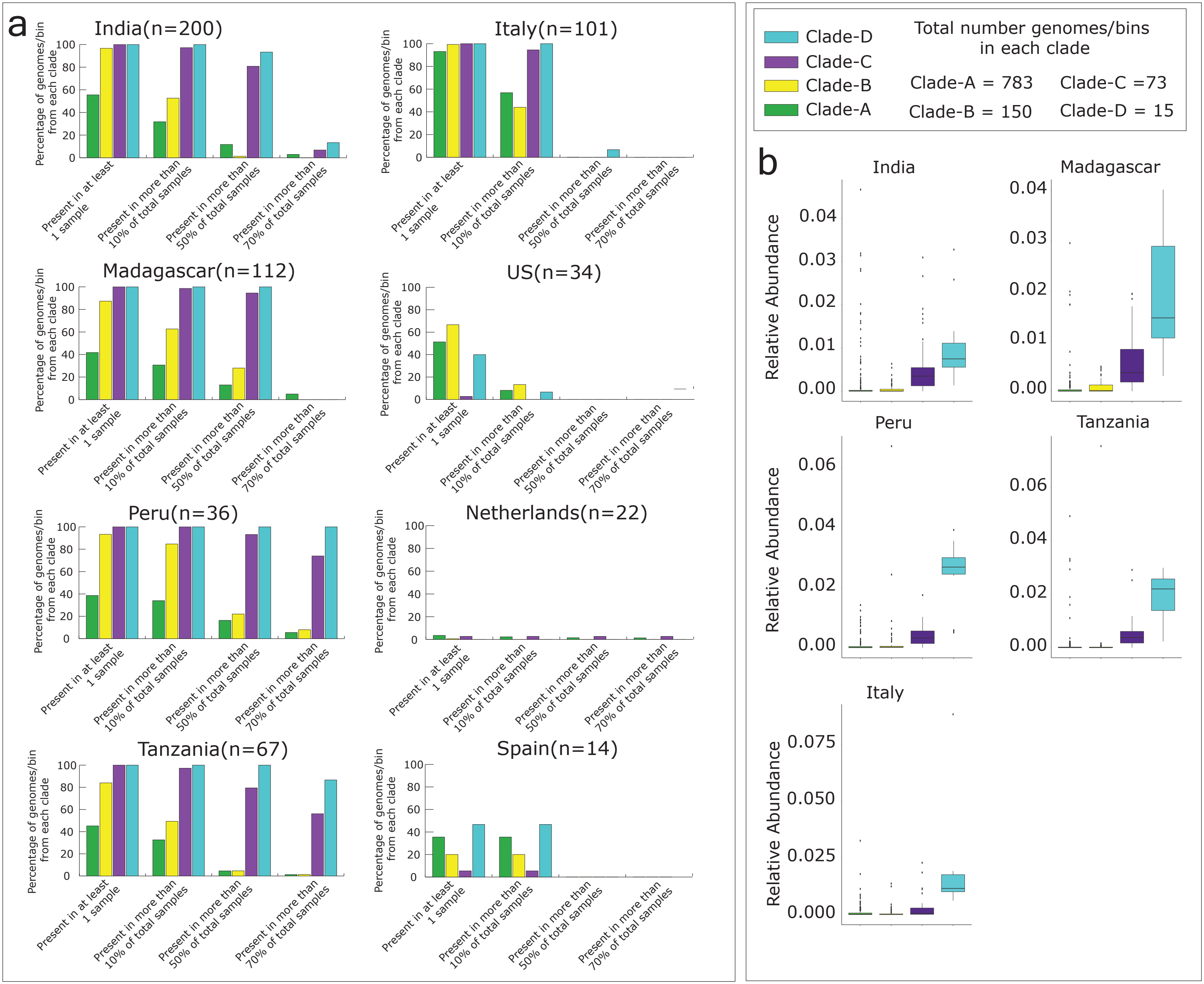

Supplementary Figure13: Composition of *P. copri* clades in different healthy populations

a) Representation of genomes/bins from each clade in different healthy populations. It is represented using four different criteria i.e., for each population, percentage of genomes/bins from each clade was calculated by applying the criteria that they should present in at least one sample, more than 10% of the samples, more than 50% of the samples and more than 70% of the samples.

b) Relative abundance of four *P. copri* clades in each population

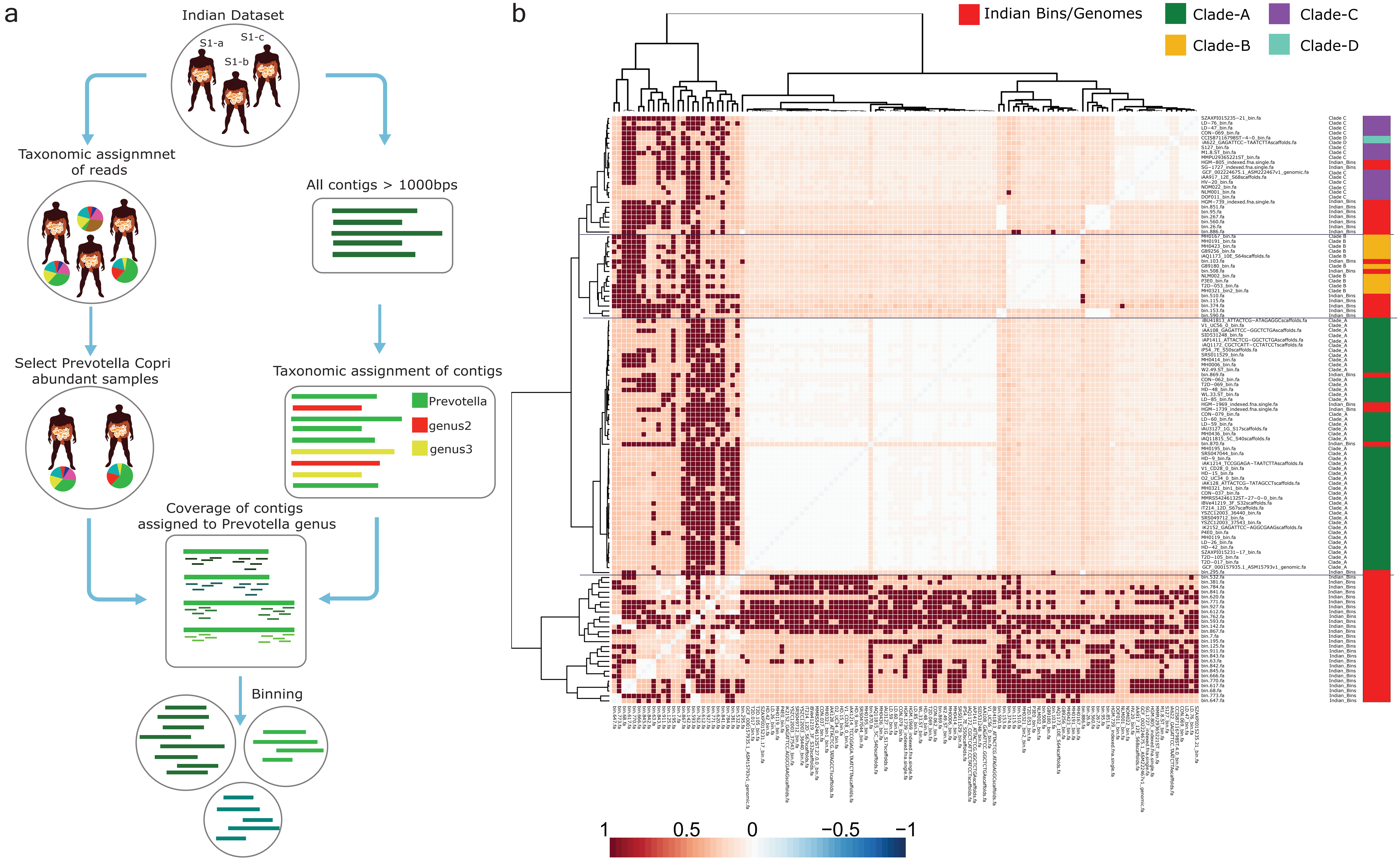

Supplementary Figure14: *Prevotella copri* bins in Indian population

a) Schematic diagram showing the method of *P. copri* specific bin reconstruction from Indian gut microbiome samples. Contigs >1,000bp from Indian population were classified using CAT/BAT and the contigs assigned to *Prevotella* genus were selected. The reads from 116 samples (Out of 200 Indian samples, 116 samples had >10% abundance of *P. copri*, as estimated by Kaiju analysis) were aligned against the *Prevotella* contigs to estimate the coverage of each contig. *Prevotella* bins were constructed using contig coverage and tetranucleotide frequency.

b) Pairwise intergenomic distances of each genome/bin were calculated using MASH For clade level assignment of 47 (42 bins+ 5 isolates) *P. copri* genomes/bins. Heatmap represent the MASH distance between each bin and blue horizontal lines represent the demarcation between each clade.

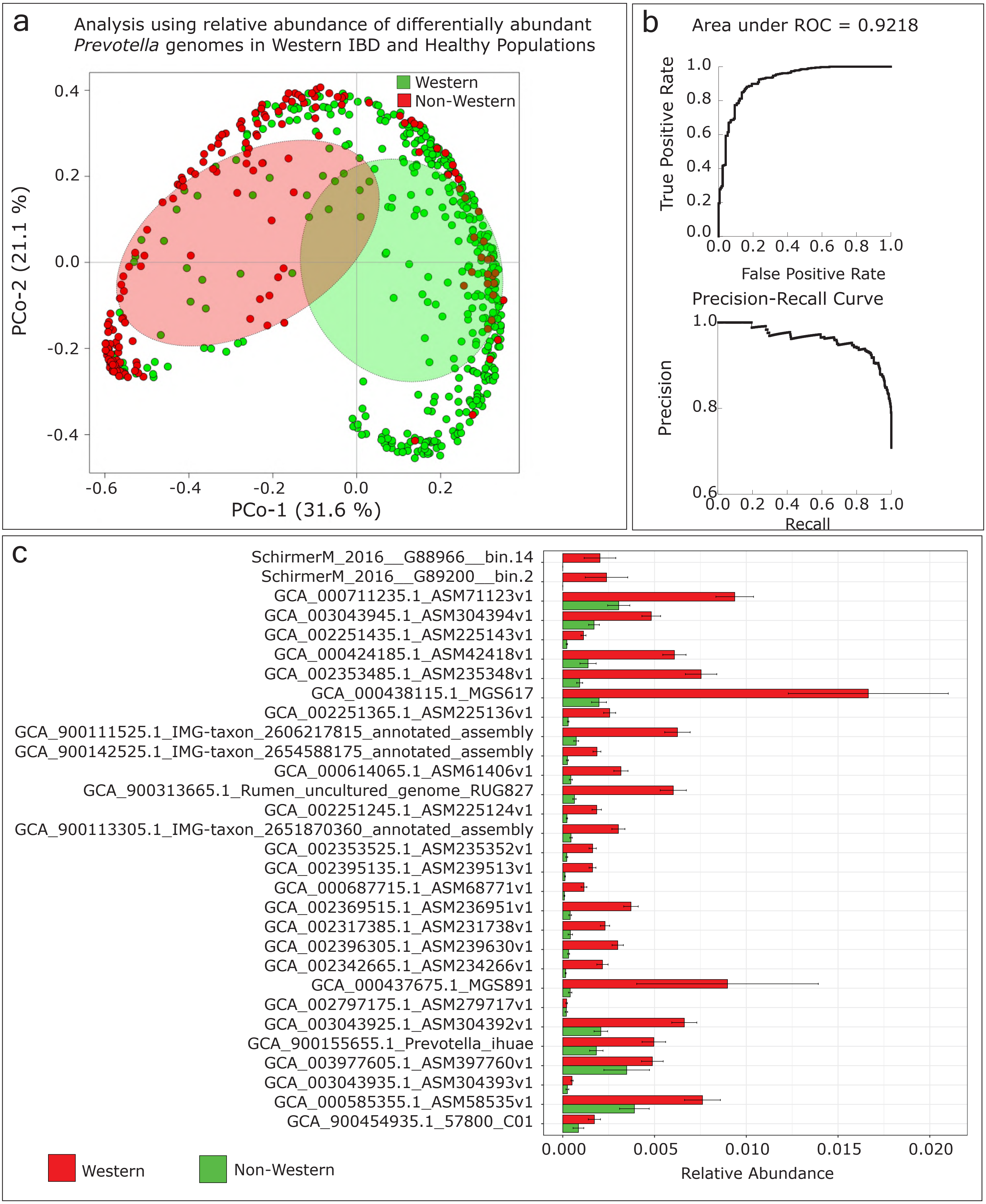

Supplementary Figure 15: Composition of 30 differentially abundant *Prevotella* genomes/bins (IBD and Healthy cohorts) in western and non-western healthy populations a) Principal coordinates analysis based on the composition of 30 differentially abundant *Prevotella* genomes in western and non-western populations.
b) ROC curve and Precision-Recall curve of randomForest classification of samples (into western and non-western populations) based on the relative abundance of 30 differently abundant genomes.
c) 30 differentially abundant *Prevotella* genomes in healthy and IBD samples were identified using labdsv package. Figure shows the relative abundance of these 30 genomes in western and non-western populations.
Error bars of bar-plots represent plus or minus one standard error of the mean

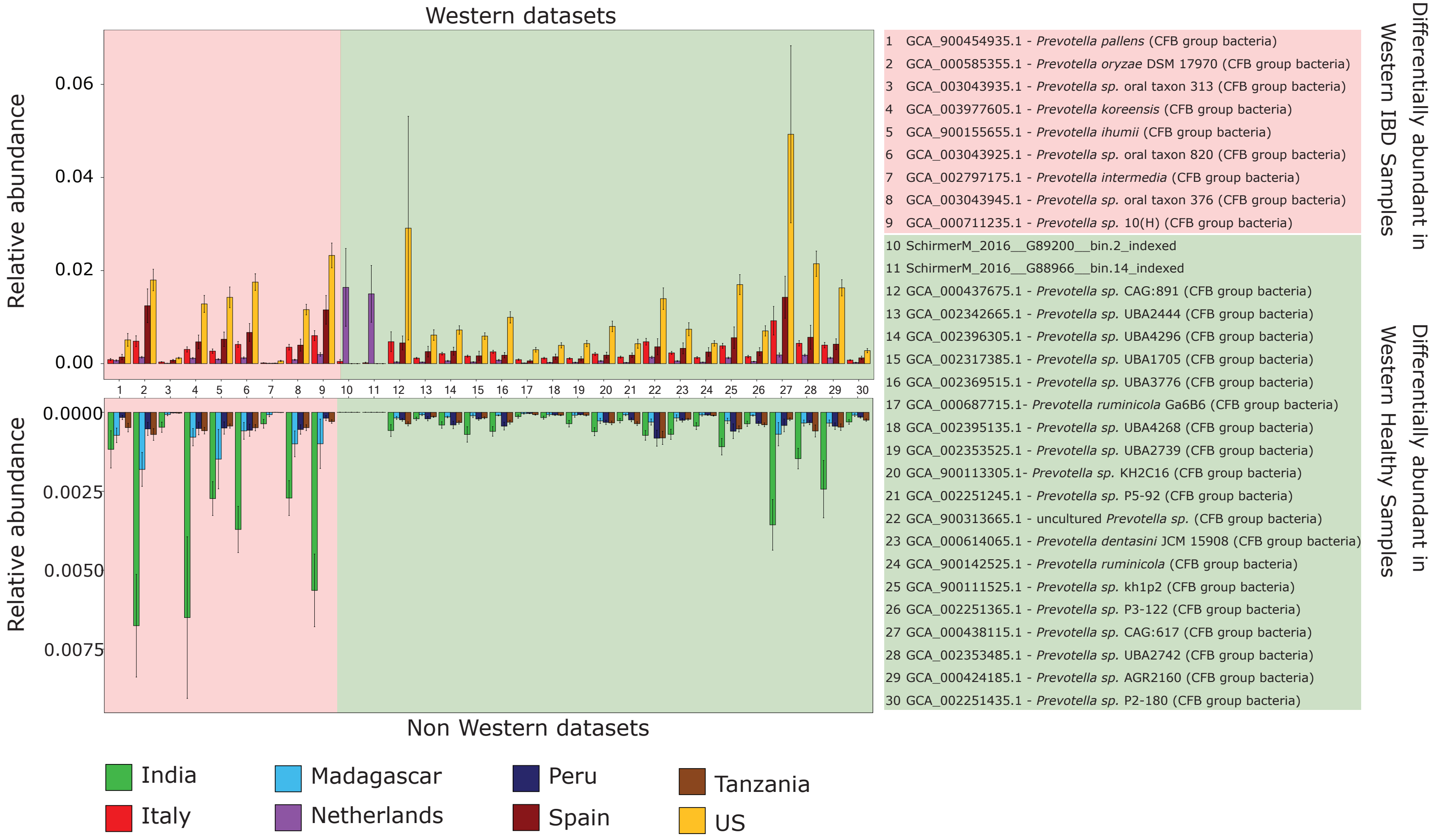

Supplementary Figure 16: Relative abundance of genomes that are differentially abundant in healthy and IBD cohorts in western and non-western populations. Bar-plots highlighted with lighter shade of Red color are differentially abundant in IBD samples and bar-plots highlighted with lighter shade of Green color are differentially abundant in healthy samples. Error bars represent plus or minus one standard error of the mean

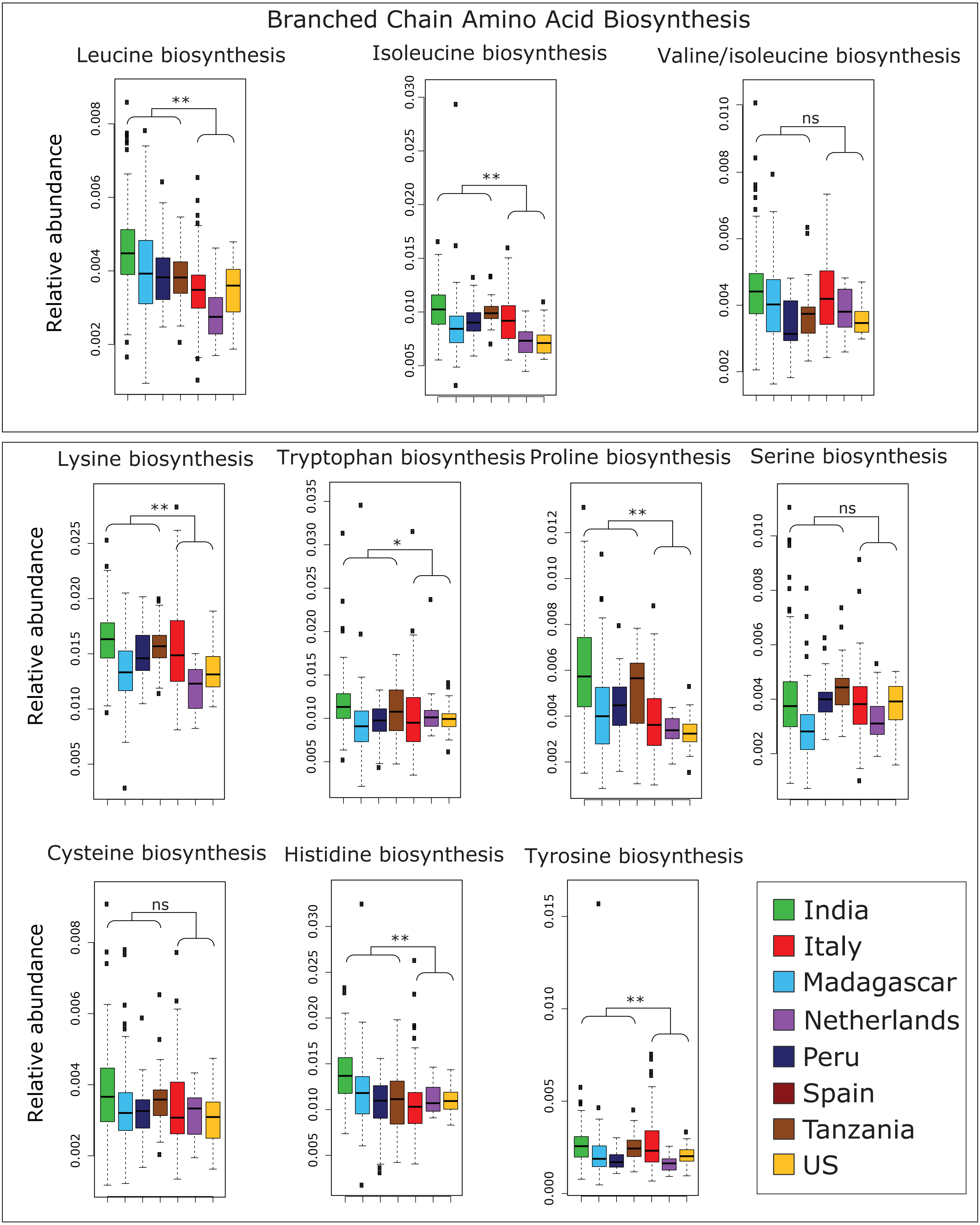

Supplementary Figure 17: Relative abundance of amino acid biosynthesis related pathways in *Prevotella* genomes in all eight healthy populations. The line in the middle of the box, bound of the box and whiskers represent the median, 25th–75th percentiles, and min-to-max values, respectively. A nonparametric two-sided Wilcoxon rank sum test was used for testing the box-plot distributions. ns, not significant; *, p-value < 0.05; **, P < 0.01.

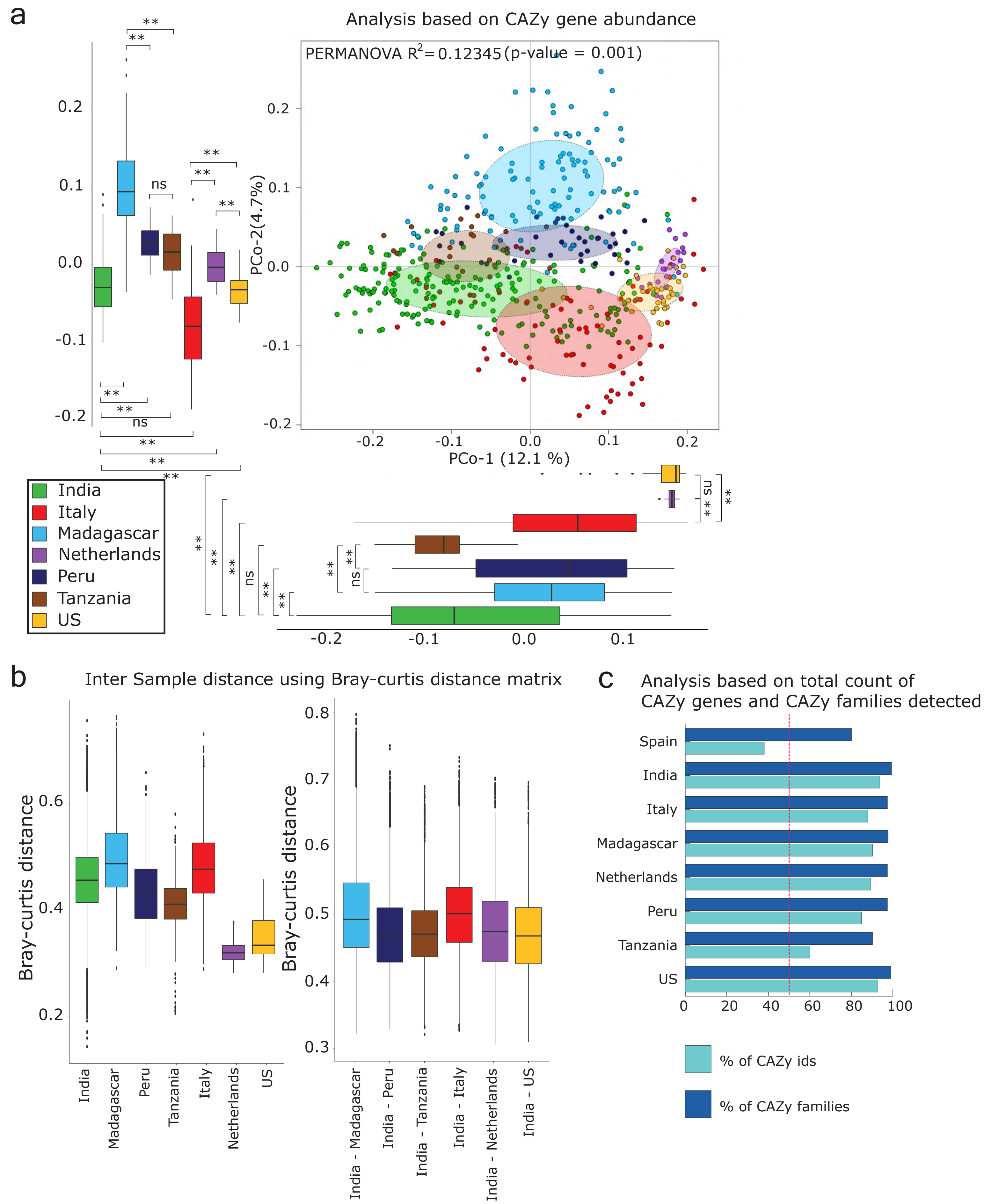

Supplementary Figure 18: Composition of CAZy genes from *Prevotella* genomes/bins in different populations
a) Principal coordinates analysis considering inter-sample Bray-Curtis distance based on relative abundance of CAZy genes of PGC (using blastp) in healthy populations.
b) Box plot showing the average inter-sample distance (Bray-Curtis) of each population and the average inter-sample distance (Bray-Curtis) between Indian samples with other populations based on relative abundance of CAZy genes.
c) Percentage of CAZy genes and CAZy gene families detected in each population. Spain was excluded from the analysis because of lower representation (< 50%) of CAZy genes.
The line in the middle of the box, bound of the box and whiskers represent the median, 25th–75th percentiles, and min-to-max values, respectively. Kruskal-Wallis test was used for testing the box plot distributions.
ns, not significant; *, p-value < 0.05; **, p-value < 0.01.

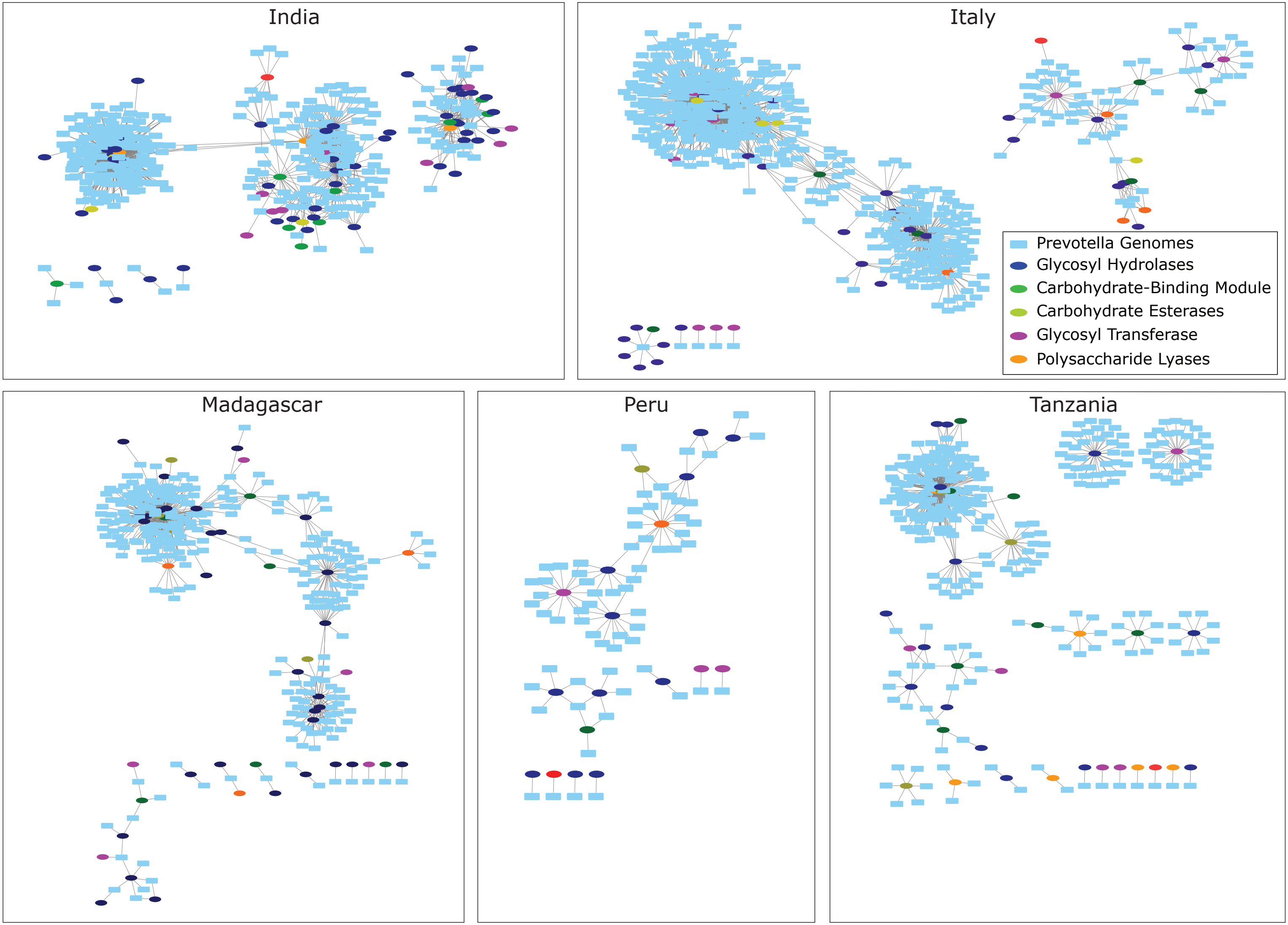

Supplementary Figure 19: Co-occurrence of *Prevotella* genomes and CAZy gene families using correlation values from ccrepe analysis. Analysis has been conducted separately for each population

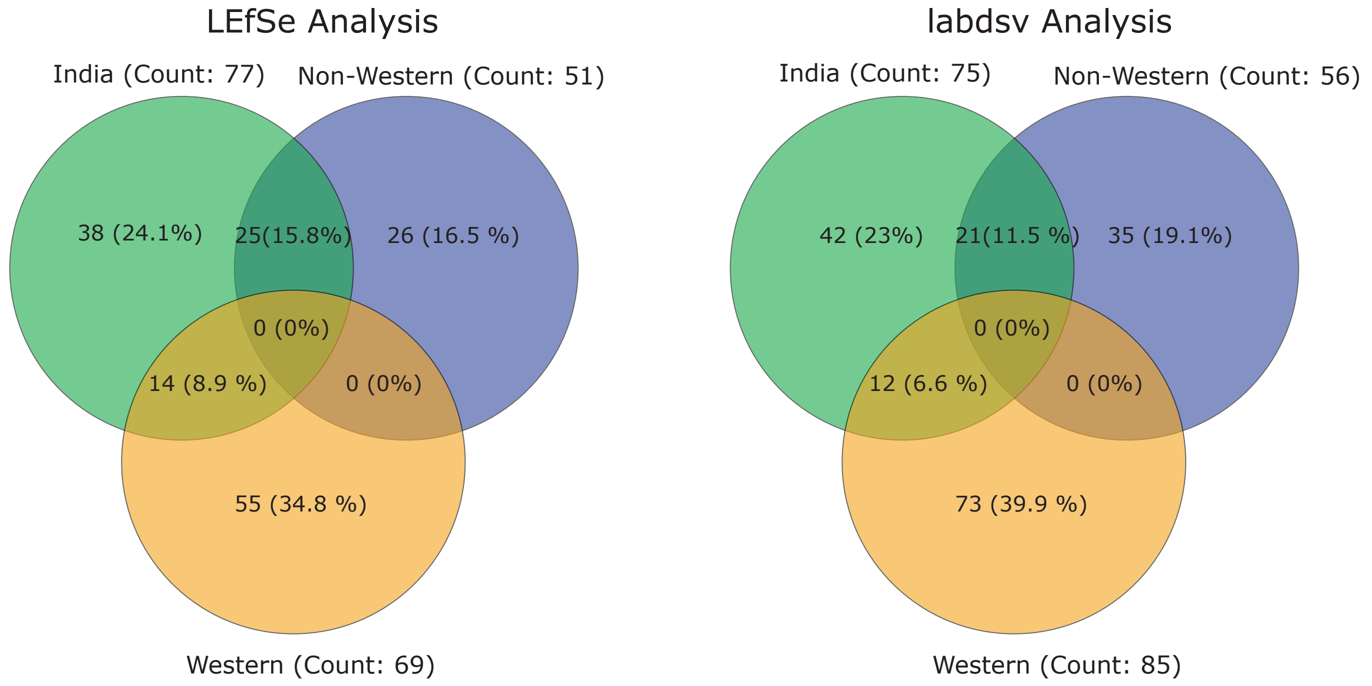

Supplementary Figure 20: Venn diagrams showing the number of discriminating CAZy families in Indian population (Compared to all other populations) and their overlap with number of differentially abundant CAZy families in western and non-western populations.

Number of PULs in *Prevotella* genomes that are differentially abundant in Western and Non-Western populations

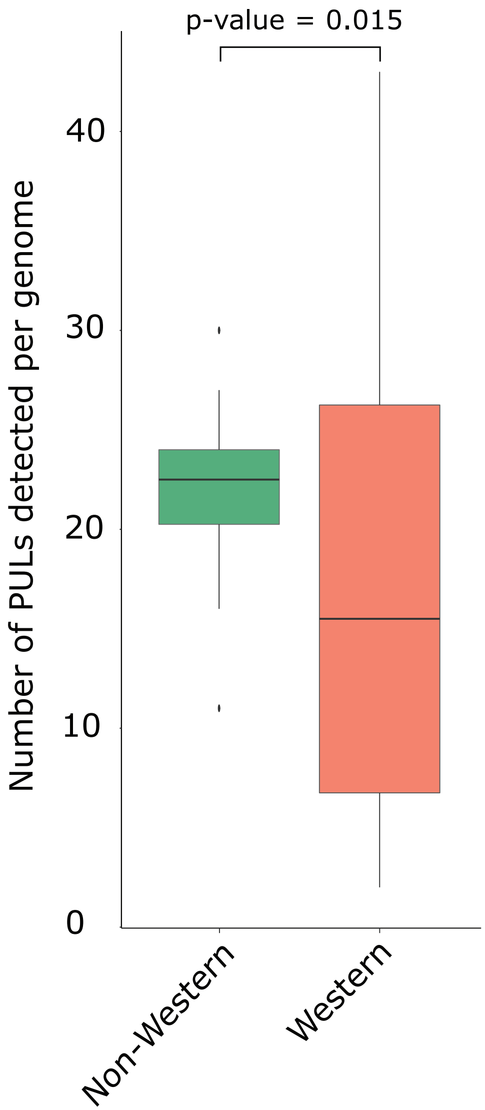

Supplementary Figure 21: Prediction of Polysaccharide Utilization Loci (PULs) from 2,204 genomes/bins in the *Prevotella* genome database using PULpy. Box-plot represent number of PULs detected per *Prevotella* genome differentially abundant in western and non-western population. The line in the middle of the box, bound of the box and whiskers represent the median, 25th–75th percentiles, and min-to-max values, respectively.
A nonparametric two-sided Wilcoxon rank sum test was used for testing the box plot distributions. Nonparametric two-sided Wilcoxon rank sum test was used to test the box plot distributions.

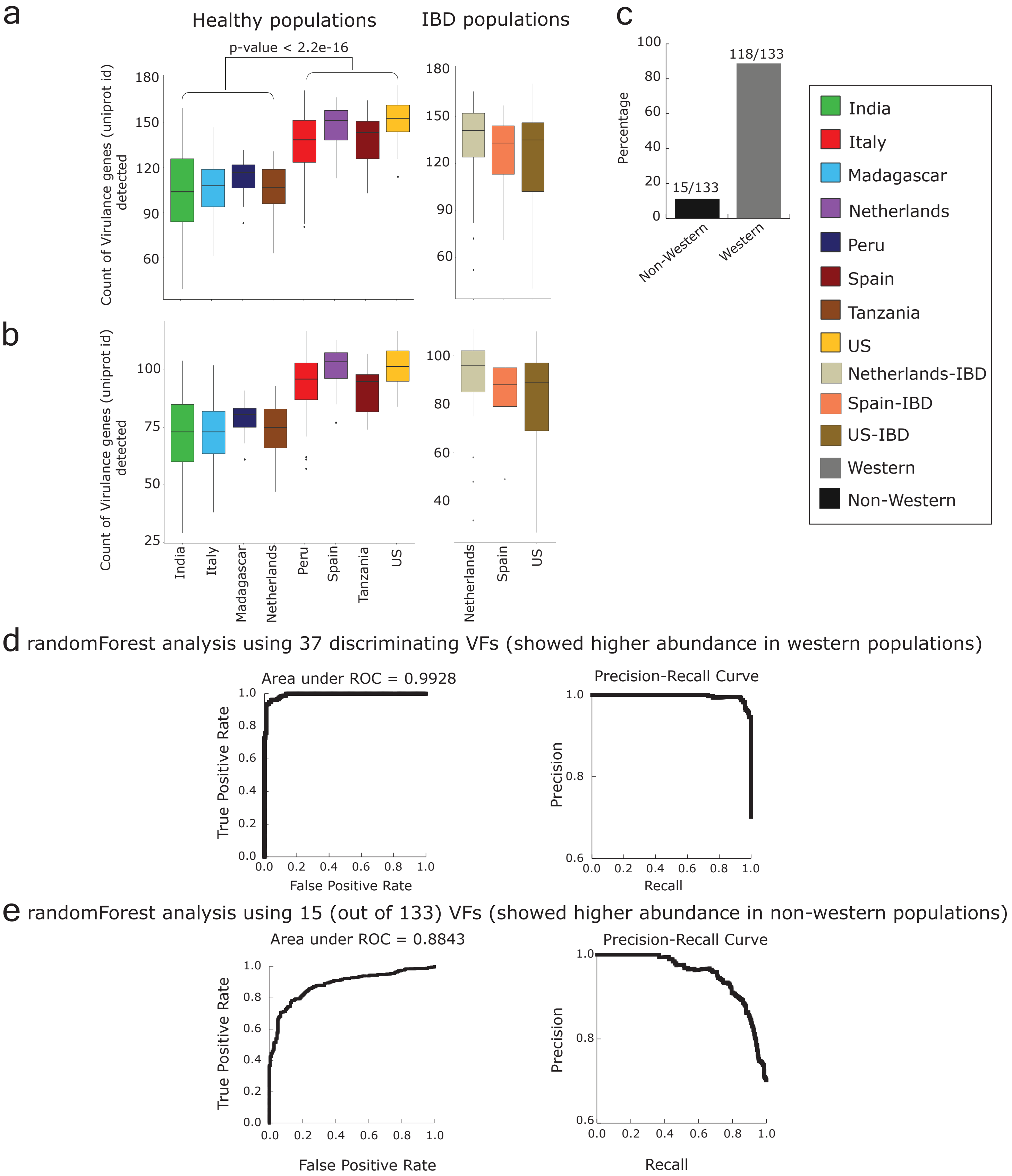

Supplementary Figure 22: Virulence factors present in *Prevotella* genomes and their abundance in different populations

a) Total number of VF genes detected in each population (Healthy and IBD). Considering best hits after homology search against full protein set of VFDB.

b) Total number of VF genes detected in each population (Healthy and IBD). Considering best hits after homology search against core protein set of VFDB.

c) Number of VF genes that showed higher abundance in western and non-western populations.

d) ROC curve and Precision-Recall curve of randomForest classification of samples (into western and non-western populations) based on relative abundance of discriminating VFs

e) ROC curve and Precision-Recall curve of randomForest classification of samples (into western and non-western populations) based on relative abundance of 15 VFs showed higher abundance in non-western population.

The line in the middle of the box, bound of the box and whiskers represent the median, 25th–75th percentiles, and min-to-max values, respectively. Nonparametric two-sided Wilcoxon rank sum test was used for testing the box plot distributions.

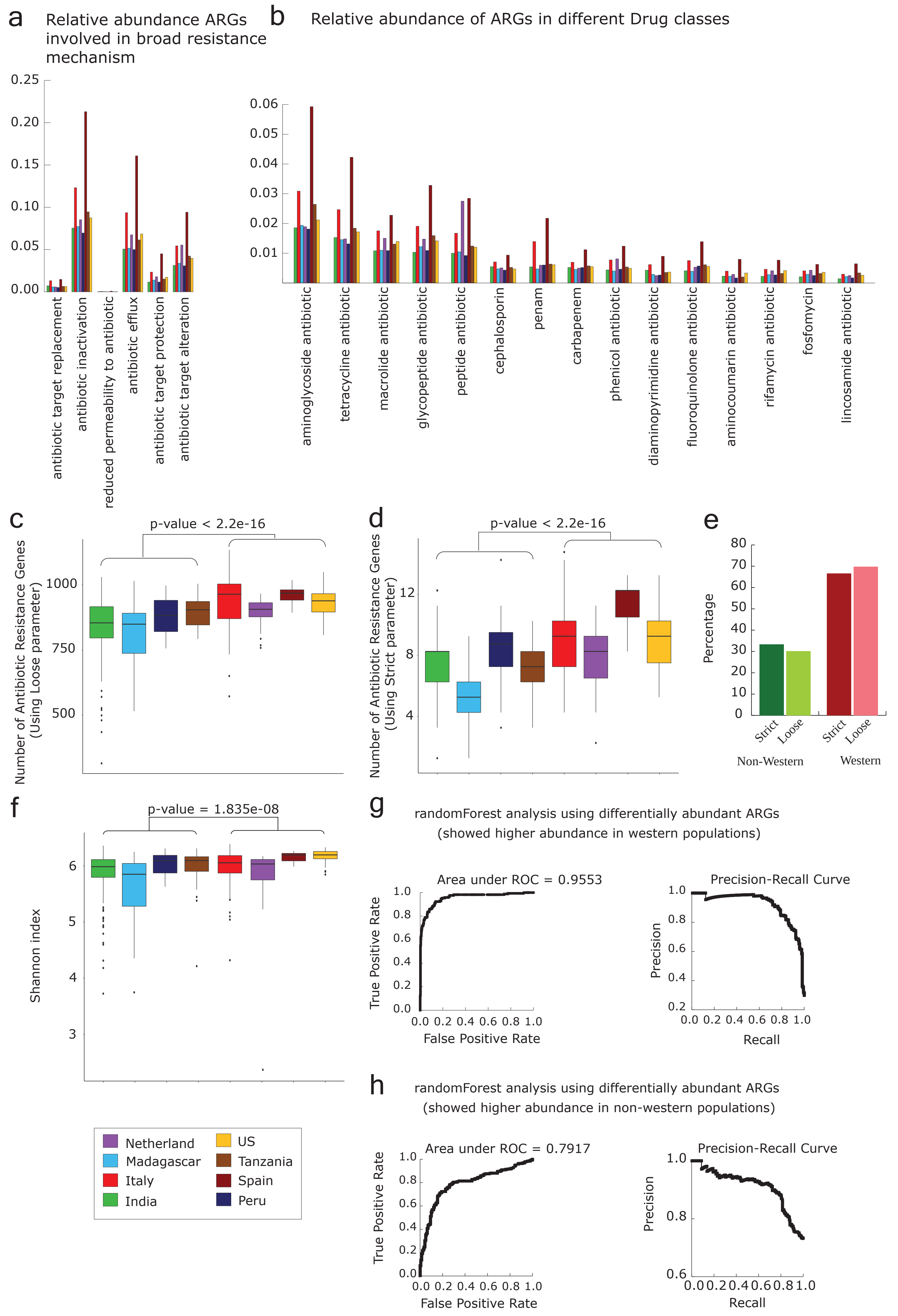

Supplementary Figure 23: Composition of antibiotic resistance genes of *Prevotella* genomes in different populations
a) Relative abundance of ARG genes mapped to the broad antibiotic resistance mechanism in different healthy populations
b) Relative abundance of ARG genes mapped to different drug classes in different healthy populations
c) Number of antibiotic resistance genes (using RGI with 'loose' parameter) present in all healthy populations
d) Number of antibiotic resistance genes (using RGI with 'strict' parameter) present in all healthy populations
e) Percentage of ARGs showed higher abundance in western and non-western populations on the basis of RGI analysis using both 'strict' and 'loose' parameter
f) Alpha diversity measure using Shannon-index based on relative abundance of ARGs in different healthy populations.
g) ROC curve and Precision-Recall curve of randomForest classification of samples (into western and non-western populations) based on relative abundance of differently abundant ARGs that showed higher abundance in western population.
h) ROC curve and Precision-Recall curve of randomForest classification of samples (into western and non-western populations) based on relative abundance of differently abundant ARGs that showed higher abundance in non-western population.
The line in the middle of the box, bound of the box and whiskers represent the median, 25th–75th percentiles, and min-to-max values, respectively. Nonparametric two-sided Wilcoxon rank sum test was used for testing the box plot distributions.

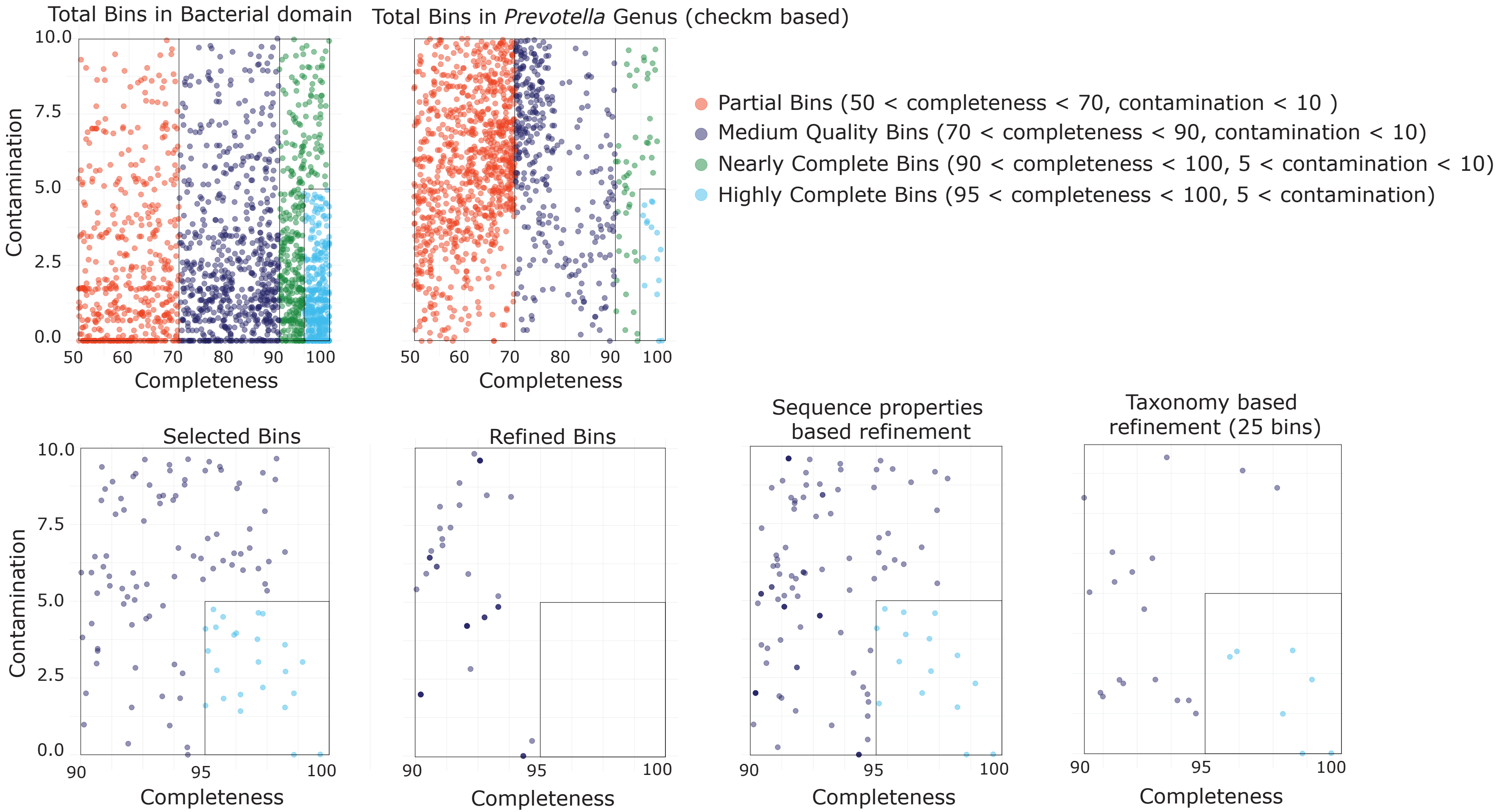

Supplementary Figure 24: Figure indicate completeness and contamination of each reconstructed bin at each stage of bin refinement (see Methods).

Supplementary Notes

1. **Supplementary Note 1: Classification of samples as western and non-western**

   The classification of populations as 'western' or 'non-western' was made on the basis of traditional lifestyle, diet, and geographic and sociodemographic definitions. Western populations include Europe, the USA and Canada[1–4] (refer https://www.worldatlas.com/articles/list-of-western-countries.html). An Intelligible definition of western and non-western classification of cohorts was mentioned in a recent study entitled "Extensive Unexplored Human Microbiome Diversity Revealed by Over 150,000 Genomes from Metagenomes Spanning Age, Geography, and Lifestyle". This study describes westernization and urbanization as synonymous terms, a complex process that occurred during the last few centuries involving profound lifestyle changes compared to populations prior to the modern era. The non-western populations that we have considered for our study were a subset of the cohorts labeled as 'non-western' populations by the study mentioned above. As described in the above-mentioned study, we adopt the terms "Westernized" and "non-Westernized" as umbrella terms to depict populations that differ by at least the majority of the above factors even though this definition comprises very heterogeneous populations.

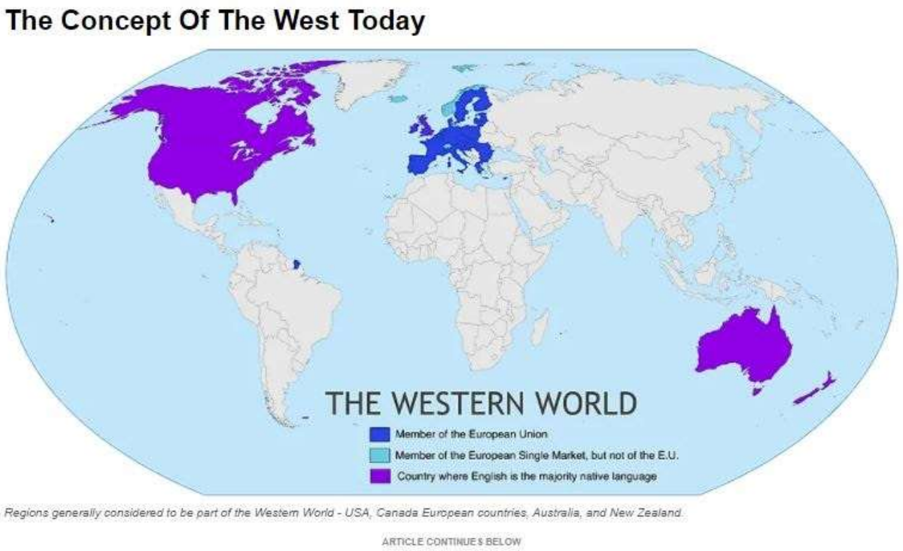

Reference: https://www.worldatlas.com/articles/list-of-western-countries.html

2. **Supplementary Note 2: Analysis of *Prevotella* genome abundance in western and non-western population**

Abundance of each genome from PGD in each sample were calculated. Twenty-six *Prevotella* genomes were present in more than 80% (468 out of 586) of samples from healthy individuals, and thus can be considered as the 'core' *Prevotella* species/strains in healthy samples. All of these 26 genomes were NCBI draft genomes. Further, 163 *Prevotella* genomes were present in more than 50% of samples (293 out of 586 samples), of which 79 were present in the NCBI reference database, 73 from reconstructed genomes/MAGs, and 11 from novel isolate assembled genomes **(Supplementary Data 5)**. A similar analysis of core *Prevotella* genomes/bins (i.e., those present in more than 80% of samples) in samples from western and non-western populations indicated a higher incidence of novel *Prevotella* species/strains in non-western populations as compared to the western populations (**Supplementary Figure 3a, b**).

Based on the average relative abundance (>1%) of *Prevotella* genomes, 17 genomes were present in all 586 healthy samples including western and non-western populations. Of those, 12 genomes were significantly abundant (Wilcoxon test) in non-western populations, and all of them were Metagenome Assembled Genomes (MAG) reconstructed from non-western datasets assigned to unclassified *Prevotella* species or strains. Of the remaining five *Prevotella* genomes, four were significantly abundant in western populations including *P. marseillensis*, *P. lascolaii* and two unclassified *Prevotella* strains (**Supplementary Figure 4a)**. The composition of *Prevotella* genomes in western and non-western population was also examined by applying >0.5% average relative abundance. The results indicate the abundance of novel genomes of *Prevotella* in non-western populations and further support the conclusions from the analyses above (**Supplementary Data 5**, **Supplementary Figure 3c, d**).

3. Supplementary Note 3: Highest inter-sample variation in the Indian population

Highest Inter sample variation (based on *Prevotella* genome abundance) could be because of the sample numbers. To address this, we have randomly selected 100 Indian samples (with 10 iterations, selecting different 100 samples each time). Then retrieved the relative abundance of *Prevotella* genomes of these 100 Indian samples to calculate Bray-Curtis distance. The box-plot shows no significant difference in Bray-Curtis distance of Indian population with the selection of 100 and 200 samples. PCoA plots using samples from these 10 iterations reveals higher spread in Indian population. PERMANOVA carried out on

Prevotella genome abundance data showed that location of sample collection has the highest and significant contribution towards the inter-sample variation in Indian population(barplot).

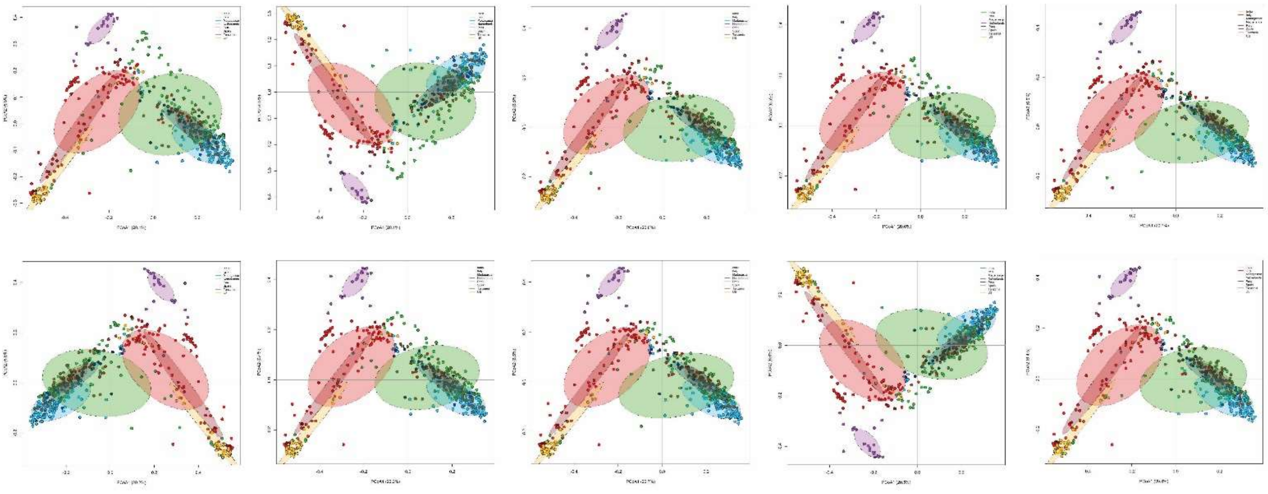

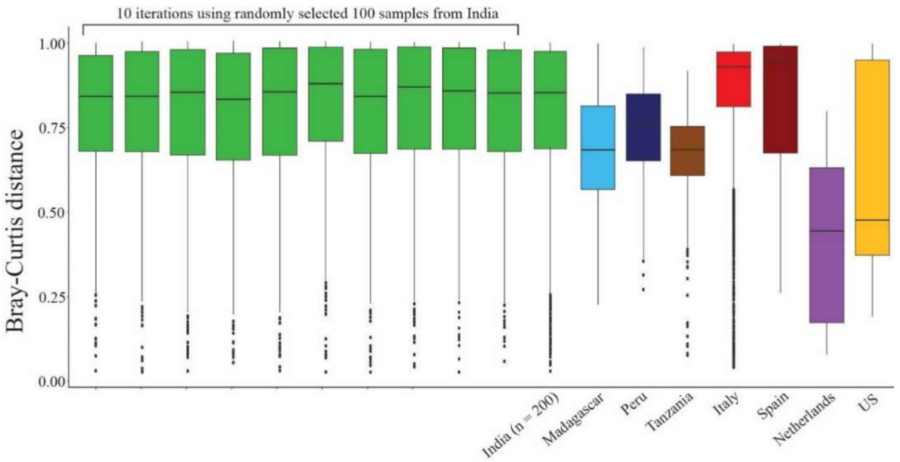

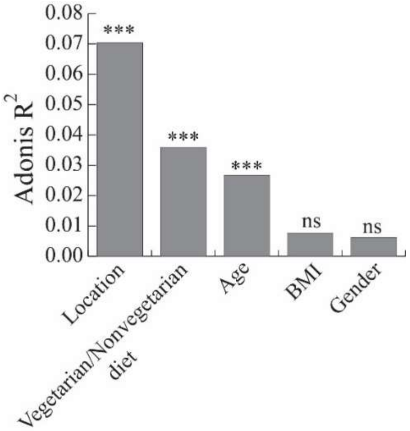

4. Supplementary Note 4: Clustering of *Prevotella* genomes and examining differentially abundant *Prevotella* genomes in western and non-western populations

Using the 2,204 genomes, we generated individual distance trees for *Prevotella* genus using the 'complete' hierarchical clustering method implemented in the Fastcluster R package[5]. We calculated the number of clusters recovered using a distance cut-off of 0.05 (95% ANI), 0.03 (97% ANI) and 0.01 (99% ANI)(Almeida et al., 2020)[6] and resulted in 228, 502 and 1856 clusters respectively.

We have clustered the 2,204 *Prevotella* genomes based on a distance cut-off of 0.00 (100% ANI) and resulted in 2,204 clusters indicated that no two genomes/bins are 100% identical. Clustering based on distance cut-off of 0.05 (95% ANI; species-level clustering) resulted in 228 clusters (**Supplementary Data 7**). These 228 clusters were named as species-level clusters, and 36 clusters (including 1740 *Prevotella* genomes; ~78.95%) out of them were having >= 10 genomes in each cluster. Annotation of these clusters was carried out by examining the clustering of 547 NCBI annotated *Prevotella* genomes out of 2204 total genomes. 213 out of 228 (93.42%) clusters were having NCBI annotated *Prevotella* genomes (**Supplementary Data 2, sheet2**). We have extracted a representative from each species-level cluster and plotted a phylogenetic tree (**Supplementary Figure 7**). Two species-level clusters (Cluster 3 and 1) were having > 100 genomes present (379 and 280 in Clusters 3 and 1, respectively), and both of them turned out to be *Prevotella copri* (**Supplementary Figure 8**). Cluster5 also contains one *P. copri* genome; *P copri indica*. Higher inter-genome diversity of *P. copri* can be a reason for assigning them into different clusters. Also, we have plotted separate phylogenetic trees for 36 species-level clusters having >= 10 genomes (**Supplementary Figure 9 and 10**).

26 and 76 differentially abundant genomes of *Prevotella* in non-western and western datasets respectively were clustered based on Mash inter-genome distance using Fastcluster R package as mentioned above. We calculated the number of clusters recovered using a distance cut-off of 0.05 (95% ANI ~ species level clustering) which resulted in 10 and 47 clusters from 26 and 76 differentially abundant *Prevotella* genomes/bins from non-western and western populations respectively. Out of 10 clusters from differentially abundant genomes/bins in non-western populations, the largest cluster (six members) was having *P. copri* (GCA_002224675.1_ASM222467v1_genomic.fna). Out of 47 clusters from differentially abundant genomes/bins in western populations, 44 were having two members in it and the remaining three were single member clusters.

5. Supplementary Note 5: Differentially abundant *Prevotella* genomes in Indian and non-western populations

We have identified 29 differentially abundant *Prevotella genomes* in Indian population compared to all other populations using labdsv (indval score > 0.50, pvalue = 0.01). Further assigned taxonomy for all these29 differentially abundant *Prevotella* genomes in Indian population using BAT. 23 out of 29 were assigned to *Prevotella copri*.

Genomes/bins differentially abundant in the Indian population	Species level taxonomic assignment
BritoIL_2016__W1.11.ST__bin.72_indexed	*Prevotella copri*
BritoIL_2016__W2.44.ST__bin.21_indexed	*Prevotella copri*
BritoIL_2016__WL.14.ST__bin.47_indexed	*Prevotella copri*
ChengpingW_2017__AS66raw__bin.10_indexed	*Prevotella copri*
DavidLA_2015__LD.Run2.36__bin.1_indexed	*Prevotella copri*
GCA_002224675.1_ASM222467v1_genomic.fna	*Prevotella copri*
GCA_003465215.1_ASM346521v1_genomic.fna	*Prevotella copri*
GCA_003467935.1_ASM346793v1_genomic.fna	*Prevotella copri*
HGM.1739_indexed	*Prevotella copri* isolate
HGM.1969_indexed	*Prevotella copri* isolate
HGM.805_indexed	*Prevotella copri* isolate
LeChatelierE_2013__MH0117__bin.12_indexed	*Prevotella copri*
LiJ_2017__H1M512834__bin.6_indexed	*Prevotella copri*
LiJ_2017__H1M513606__bin.16_indexed	Unclassified
LiJ_2017__H3M415906__bin.19_indexed	*Prevotella copri*
LiJ_2017__H3M514143__bin.14_indexed	*Prevotella copri*
LiJ_2017__H3M515128__bin.16_indexed	*Prevotella copri*
LiJ_2017__H3M515936__bin.36_indexed	*Prevotella copri*
NielsenHB_2014__MH0117__bin.8_indexed	*Prevotella copri*
QinJ_2012__CON.024__bin.14_indexed	*Prevotella copri*
SchirmerM_2016__G89093__bin.1_indexed	*Prevotella copri*
SG.1727_indexed	*Prevotella copri* isolate
US_HEALTHY_bins.701	Unclassified
US_HEALTHY_bins.702	Unclassified
ZeeviD_2015__PNP_Main_170__bin.19_indexed	Unclassified
ZeeviD_2015__PNP_Main_274__bin.41_indexed	*Prevotella copri*
ZeeviD_2015__PNP_Main_30__bin.26_indexed	*Prevotella copri*
ZeeviD_2015__PNP_Main_610__bin.43_indexed	Unclassified
ZeeviD_2015__PNP_Main_85__bin.19_indexed	Unclassified

6. Supplementary Note 6: *Prevotella copri* genome abundance analysis

Principal coordinate analysis based on abundance of *P. copri* genomes/bins indicated higher inter-sample variation in Indian population compared to other non-western populations. Similarly, the Italian population showed higher inter-sample variation compared to other western populations. PCo-1 and PCo-2 of Indian samples revealed that it is significantly different from all western populations, Italian population and non-western populations.

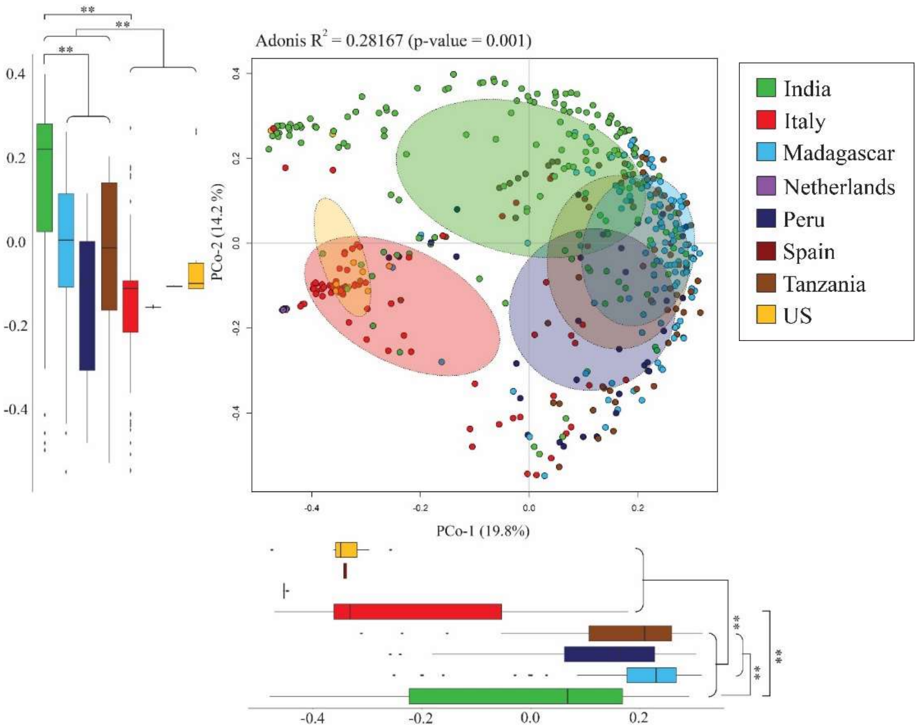

7. Supplementary Note 7: Inflammation associated *Prevotella* species

P. intermedia group bacteria, including *P. intermedia*, *P. nigrescens*, and *P. pallens* are known for their association with inflammatory conditions. These bacteria are related to periodontitis, dental abscess, brain abscess, lung abscess, bacterial vaginosis, preterm birth, and bone infection (Park et al. 2019)[11]. *P. pallens* are reported to be present mucosal surfaces as indigenous flora. The presence of *P. intermedia* and *P. pallens* in a different type of infections including periodontal disease and soft-tissue infections were reported (Könönen et al. 2000)[12]. Designated strain JS262T of *Prevotella* koreensis was isolated from the human subgingival plaque of periodontitis lesion. The biochemical characteristics of this strain (strain JS262T) were similar to those of *P. intermedia* and *P. nigrescens*. *P. pallens* were initially called as *P. intermedia/nigrescens*-like organism (PINLO) because of its phenotypical resemblance with these microbes[13,14]. Designated strains of *P. ihumii* and *P. koreensis* were isolated recently and their functional properties remain unexplored. The phylogenetic relatedness of *P. ihumii* with the *P. intermedia* group of bacteria has been reported in previous studies. It is evident from the literature that these *Prevotella* species are also involved in the complex process of biofilm formation, which is potentially connected with poor oral hygiene and involved in the cause of oral diseases, such as prevalent gingivitis and periodontitis. The phylogenetic tree given below indicates the relatedness of newly characterized *P. ihumii* species and *P. intermedia* group bacteria.

Species name	Reports regarding association with inflammatory conditions	References
Prevotella intermedia	● *P. intermedia* is an established oral pathogen belonging to the “orange complex”.<br>● The association between gingivitis and species of this group of bacteria seems to be most pronounced for *P.intermedia*, which shows a recovery rate of > 70% in adults with gingivitis<br>● *P. intermedia* identified as the second most dominant bacterium in chronic periodontitis (after Po. gingivalis). A large contribution of its transcripts to the total mapped reads in periodontitis was observed. Interestingly, *Prevotella* spp. were found to be predictive for the onset of early childhood caries<br>● The acute periodontal abscess seems most consistently to be associated with either *Porphyromonas gingivalis* or *Prevotella intermedia* or both<br>● *P. intermedia* is a rare causative agent for aortitis.<br>● Periodontitis and *P. intermedia* are associated with severe asthma	15<br>16<br>14<br>17<br>18<br>19
Prevotella koreensis	designated strain JS262T, was isolated from human subgingival plaque of periodontitis lesion in 2019	20
Prevotella pallen	● The higher abundance of *P. pallens* in addition to some other microbes (*Prevotella melaninogenica, Fusobacterium sp., Veillonella parvula, Porphyromonas endodontalis, Prevotella pallens, Dialister, Streptococcus anginosus, Prevotella nigrescens, Campylobacter ureolyticus, Prevotella nanceiensis, Peptostreptococcus anaerobius*) in in the saliva of patients having OSCC.<br>● increasing estradiol concentrations enhance the planktonic growth, ability to coaggregate with *F. nucleatum*, polysaccharide production, and biofilm formation of *P. intermedia* group bacteria (*P. intermedia, P. nigrescens, P. pallens*), in a strain-dependent manner	21<br>22<br>23<br>24<br>25

	• *P. pallens* has been identified as associated with inflammation in the female genital tract<br>• Prevalence of *P. pallens* in biota from a patient with Barrett's esophagus<br>• Presence of *P. pallens* among the isolates from CF patients	
Prevotella ihumii	• *P. ihumii strain Marseille*-P3385T (= CSURP3385T; = DSM106428T) is a new species isolated from a fresh stool specimen of a healthy woman	26
Prevotella nigrescens	• A recent study in mice has shown that *P. nigrescens*, similarly to *P. gingivalis*, can drive periodontal disease (demonstrated by maxillary alveolar bone loss following oral inoculation)<br>• The higher abundance of *P. nigrescens* in addition to some other microbes (*Prevotella melaninogenica, Fusobacterium sp., Veillonella parvula, Porphyromonas endodontalis, Prevotella pallens, Dialister, Streptococcus anginosus, Prevotella pallens, Campylobacter ureolyticus, Prevotella nanceiensis, Peptostreptococcus anaerobius*) in the saliva of patients having OSCC.<br>• This cross-sectional study showed that P. nigrescens and *P. gingivalis* were significantly associated with increased calculated intima-media area (cIMA) values. Indicates the associated with signs of carotid atherosclerosis in subjects with and without periodontitis<br>• Infection with *P. nigrescens* induces TLR2 signalling and low levels of p65 mediated inflammation in Cystic Fibrosis bronchial epithelial cells<br>• *P. nigrescens* LPS can induce iNOS expression and stimulate the release of NO without additional stimuli and has an important role of the transcription factor NF-κB and microtubule polymerization in NO production.	27<br>28<br>29

8. Supplementary Note 8: Detailed analysis of plant carbohydrate metabolizing enzymes in Indian and other non-western populations

The analysis was carried out using CAZy (carbohydrate active enzymes) database that contains the carbohydrate metabolizing enzymes broadly categorized as Glycosyl Hydrolases (GHs),

Glycosyl Transferases (GTs), Carbohydrate-Binding Modules (CBMs), Carbohydrate Esterases (CEs) and Polysaccharide Lyases (PLs) [7]. Principal coordinate analysis based on the abundance of CAZy genes resulted in clustering of the Indian and Tanzanian populations. This analysis also clustered the US and Netherlands populations (**Supplementary Figure 18a**). Further, this analysis clustered the US and Netherlands populations that also displayed the lowest average inter-sample distances based on Bray-Curtis distance matrix, which corroborates with the clustering of these two populations based on *Prevotella* genome abundance (**Figure 2**) suggesting a similarity in *Prevotella*-associated carbohydrate metabolizing activity in these two populations (**Supplementary Figure 18b**).

Among the various CAZy enzyme categories, the analysis of GH family of enzymes is interesting since they are involved in hydrolysis or rearrangement of glycosidic bonds and are major contributors of carbohydrate degradation. The genes encoding differentially abundant GHs were classified into three groups based on their utilization of carbohydrate substrates of plant, animal and mucin origin, as in the classification of Kaoutari et al. 2013[8] and Smits et al. 2017[9] (**Supplementary Data 12**). Among them, ten CAZy families (GH13, GH13_14, GH2, GH43_24, GH43_4, GH43_5, GH29, GH3, GH31 and GH51) were also highly abundant in the *Prevotella* genomes from the Tanzanian population. These GH families are reported to be highly prevalent in *P. copri* clades and mainly involved in Pectin/Hemicellulose and starch metabolism. This observation further supports the relatedness of Indian and Tanzanian populations based on CAZy gene composition revealed by principal coordinate analysis. These GH genes were also significantly abundant in non-western populations when compared to western populations (p-value < 0.01, Wilcoxon). Although three (GH43_24, GH43_4, GH43_5) of the six subfamilies of GH43 were differentially abundant in the Indian and Tanzanian populations, the remaining three subfamilies of GH43 (GH43_16, GH43_18 and GH43_31) also discriminated the Indian cohort from other populations. Of these, GH43_18 and GH43_31 was significantly abundant in western populations compared to other non-western populations (Madagascar, Peru and Tanzania; p-value < 0.01, Wilcoxon). GH2, GH3 and GH13 were among the five GHs highly abundant in the Indian population. GH12 and GH78 family were also more abundant in the Indian cohort. GH 27 and GH36 were also found discriminating Indian population from other populations, though a comparable abundance was observed in US and Netherlands populations **(Figure 6a, b)**.

9. **Supplementary Note 9: Differentially abundant CAZy families in Indian population compared to all other populations.**

GHs that are highly abundant and discriminate Indian populations from all other healthy populations and not belong to plant-carbohydrate metabolizing group classified by Kaoutari et al. 2013 and Smits et al. 2017 include GH130, GH92, GH18 and GH127. GH127 and GH92, and showed high abundance in Tanzanian and Indian populations. Another family of CAZy enzymes i.e., CE11 family of carbohydrate esterase enzymes showed higher abundance in western population compared to non-western populations. The other enzymes CE15, CE6 and CE9 from CE family showed high abundance in non-western population compared to the western population.

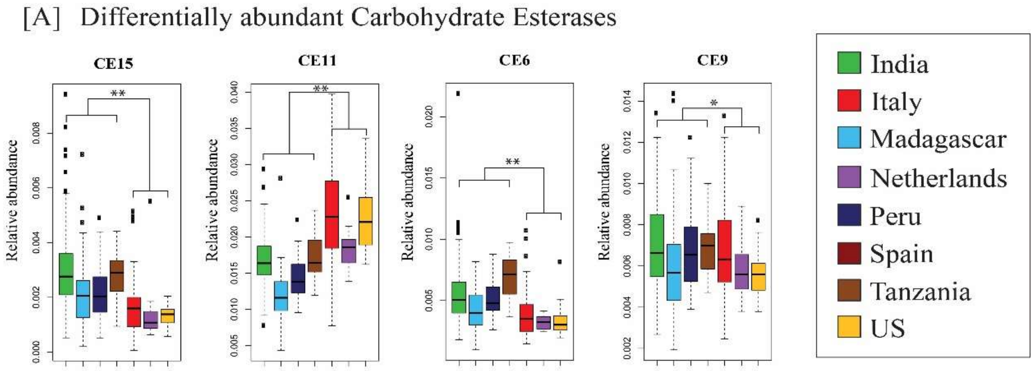

10. **Supplementary Note 10: Detailed analysis of pullulanase-containing-PULs in *Prevotella copri* genomes**

Pullulanase gene (GH13_14) was present in PULs at 794 *Prevotella copri* genomes (out of 1023 genomes). 782 out of 794 were having pullulanase-containing-PULs reported in our study (GH77, GH97, GH13_14 and GH13). 569 out of 782 were having GH43_4 and GH43_5 with aforementioned pullulanase-containing-PULs. 168 PULs were having GH51 with GH77,

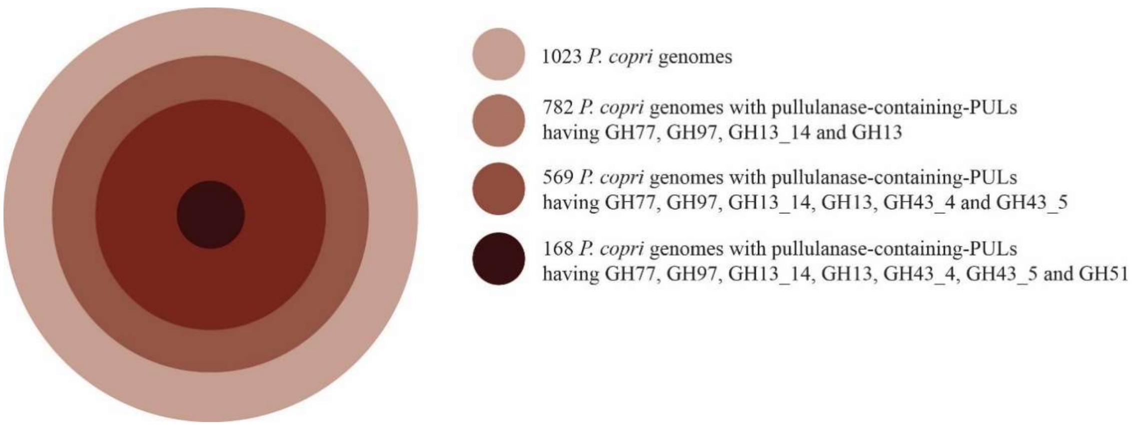

GH97, GH13_14, GH13, GH43_4 and GH43_5. The arrangements of these CAZy genes can be different in these PULs.

Further, 773 out of 782 contigs have Neopullulanase_SusA and pullulanase (involved in the metabolism of α-1,4 and α-1,6-linkages present in starch-derived glucans, respectively) in the same PUL. Operon prediction analysis (using operon-mapper) revealed that these two genes are in same operon (with >95% probability).

The order and frequency of genes present in the pullulanase-containing PULs revealed more clear understanding of these PULs in starch and non-starch metabolism of plant-polysaccharides, mainly cereal grains (**Tables (i), (ii), (iii), (iv)**).

Table (i): Order of genes in the PUL region of contigs containing pullulanase-containing-PULs (with CAZy families including GH77, GH97, GH13_14, GH13, GH43_4, GH43_5 and GH51)

Order of genes	Gene id and function
1	Starch-binding_protein_SusD
2	TonB-dependent_receptor_SusC
3	HTH-type_transcriptional_repressor_CytR
4	General_alpha-glucoside_permease
5	4-alpha-glucanotransferase_DPE2
6	Glucan_14-alpha-glucosidase_SusB
7	Pullulanase
8	Neopullulanase_SusA
9	'hypothetical protein'
10	'hypothetical protein'
11	HTH-type_transcriptional_activator_RhaS
12	TonB-dependent_receptor_SusC
13	'hypothetical protein'
14	'hypothetical protein'
15	Extracellular_endo-alpha-(1->5)-L-arabinanase
16	Intracellular_endo-alpha-(1->5)-L-arabinanase
17	Uncharacterized_protein_in_vnfD_5'region
18	Putative_permease_MJ0326
19	Extracellular_exo-alpha-L-arabinofuranosidase

Table (ii): Frequency of each gene in the PUL region of 168 contigs having pullulanase-containing-PULs (with CAZy families including GH77, GH97, GH13_14, GH13, GH43_4, GH43_5 and GH51).

pullulanase-containing-PULs	
**Gene id**	**frequency**
TonB-dependent_receptor_SusC	354
Glucan_14-alpha-glucosidase_SusB	169
Pullulanase	168
Neopullulanase_SusA	168
Extracellular_exo-alpha-L-arabinofuranosidase	168
4-alpha-glucanotransferase_DPE2	168
Putative_permease_MJ0326	163
HTH-type_transcriptional_repressor_CytR	151
Starch-binding_protein_SusD	141
Intracellular_endo-alpha-(1->5)-L-arabinanase	134
HTH-type_transcriptional_activator_RhaS	122
Extracellular_endo-alpha-(1->5)-L-arabinanase	120
Sucrose_transport_protein_SUT5	111
Uncharacterized_protein_in_vnfD_5'region	77
Extracellular_endo-alpha-(1->5)-L-arabinanase_2	47
Extracellular_exo-alpha-(1->5)-L-arabinofuranosidase_ArbA	36
General_alpha-glucoside_permease	35
Uncharacterized_protein_HI_1038	30
Uncharacterized_protein_MJECL04	26
Adenine_permease_AdeP	4
Outer_membrane_protein_SusE	1
Outer_membrane_protein_SusF	1
Uncharacterized_protein_in_bgaB_5'region_(Fragment)	1

Table (iii): Order of genes in the PUL region of 569 contigs containing pullulanase-containing-PULs (with CAZy families including GH77, GH97, GH13_14, GH13, GH43_4 and GH43_5)

Order of genes	Gene id and function
1	Starch-binding_protein_SusD
2	TonB-dependent_receptor_SusC
3	HTH-type_transcriptional_repressor_CytR

4	Sucrose_transport_protein_SUT5
5	4-alpha-glucanotransferase_DPE2
6	Glucan_14-alpha-glucosidase_SusB
7	Pullulanase
8	Neopullulanase_SusA
9	'hypothetical protein'
10	HTH-type_transcriptional_activator_RhaS
11	TonB-dependent_receptor_SusC
12	'hypothetical protein'
13	'hypothetical protein'
14	Extracellular_endo-alpha-(1->5)-L-arabinanase
15	Intracellular_endo-alpha-(1->5)-L-arabinanase

Table (iv): Frequency of each gene in the PUL region of 569 contigs having pullulanase-containing PULs (with CAZy families including GH77, GH97, GH13_14, GH13, GH43_4 and GH43_5)

**pullulanase-containing-PULs**	
**Gene id**	**frequency**
TonB-dependent_receptor_SusC	1267
Glucan_14-alpha-glucosidase_SusB	570
Pullulanase	569
Neopullulanase_SusA	569
4-alpha-glucanotransferase_DPE2	569
Intracellular_endo-alpha-(1->5)-L-arabinanase	463
Starch-binding_protein_SusD	447
HTH-type_transcriptional_activator_RhaS	431
HTH-type_transcriptional_repressor_CytR	425
Extracellular_endo-alpha-(1->5)-L-arabinanase	307
Sucrose_transport_protein_SUT5	283
Extracellular_endo-alpha-(1->5)-L-arabinanase_2	225
Extracellular_exo-alpha-L-arabinofuranosidase	167
Putative_permease_MJ0326	150
General_alpha-glucoside_permease	137
Extracellular_exo-alpha-(1->5)-L-arabinofuranosidase_ArbA	135
Uncharacterized_protein_in_vnfD_5'region	77
HTH-type_transcriptional_regulator_GalS	61
Proton-associated_sugar_transporter_A	53

Uncharacterized_protein_HI_1038	29
Uncharacterized_protein_MJECL04	23
Adenine_permease_AdeP	17
Membrane-associated_transporter_protein	11
Outer_membrane_protein_SusE	7
Outer_membrane_protein_SusF	7
HTH-type_transcriptional_regulator_YesS	7
HTH-type_transcriptional_regulator_GalR	7
Ribose_operon_repressor	6
Putative_beta-xylosidase	6
Alpha-amylase_type_B_isozyme	4
Uncharacterized_protein_in_bgaB_5'region_(Fragment)	3
Putative_sucrose_transport_protein_SUC6	3
HTH-type_transcriptional_repressor_PurR	2
Uncharacterized_glycosyltransferase_RF_0543	2
UDP-glucuronic_acid_decarboxylase_4	2
Alpha-L-arabinofuranosidase_1	1

11. Supplementary Note 11: Number of PULs having different arrangements of CAZy families (GH77, GH97, GH13_14, GH13, GH43_4, GH43_5 and GH51) in *P.copri* genomes

Pullulanase gene (GH13_14) was present in PULs at 794 *Prevotella copri* genomes (out of 1,023 genomes). 782 out of 794 were having pullulanase-containing-PULs reported in our study (GH77, GH97, GH13_14 and GH13). Frequency of pullulanase containing PULs detected in 794 out of 1,023 *P.copri* genomes are given in the table below.

**PULs**	**Frequency**
GH13-GH13_14-GH97-GH77-unk-unk-susC-susD	57
susD-susC-unk-unk-GH77-GH97-GH13_14-GH13	56
susD-susC-unk-unk-GH77-GH97-GH13_14-GH13-unk-susC-susD-unk-GH43_4-GH43_5	48
GH43_5-GH43_4-unk-susD-susC-unk-GH13-GH13_14-GH97-GH77-unk-unk-susC-susD	45
GH43_5-GH43_4-susD-susC-susD-susC-unk-unk-GH13-GH13_14-GH97-GH77-unk-unk-susC-susD	38
susD-susC-unk-unk-GH77-GH97-GH13_14-GH13-unk-unk-unk-susC-susD-unk-GH43_4-GH43_5	37
susD-susC-unk-unk-GH77-GH97-GH13_14-GH13-unk-unk-susC-susD-susC-susD-GH43_4-GH43_5	33

susD-susC-unk-unk-GH77-GH97-GH13_14-GH13-unk-unk-unk-unk-susC-susD-susC-susD-GH43_4-GH43_5	30
GH43_5-GH43_4-unk-susD-susC-unk-unk-unk-GH13-GH13_14-GH97-GH77-unk-unk-susC-susD	25
GH43_5-GH43_4-susD-susC-susD-susC-unk-unk-unk-unk-GH13-GH13_14-GH97-GH77-unk-unk-susC-susD	24
susD-susC-unk-unk-GH77-GH97-GH13_14-GH13-unk-susC-susD-unk-GH43_4-GH43_5-unk-unk-GH51	21
GH51-unk-unk-GH43_5-GH43_4-unk-susD-susC-unk-GH13-GH13_14-GH97-GH77-unk-unk-susC-susD	13
GH77-GH97-GH13_14-GH13-unk-unk-unk-unk-susC-susD-susC-susD-GH43_4-GH43_5	13
susD-susC-unk-unk-GH77-GH97-GH13_14-GH13-unk-unk-susC-susD-susC-susD-GH43_4-GH43_5-unk-unk-GH51	10
susD-susC-unk-unk-GH77-GH97-GH13_14-GH13-unk-unk-unk-unk-susC-susD-unk-GH43_4-GH43_5	9
GH43_5-GH43_4-unk-susD-susC-unk-unk-unk-unk-GH13-GH13_14-GH97-GH77-unk-unk-susC-susD	7
susD-susC-unk-unk-GH77-GH97-GH13_14-GH13-unk-unk-unk-susC-susD-unk-GH43_4-GH43_5-unk-unk-GH51	7
GH13-GH13_14-GH97-GH77-unk-unk-unk-susC-susD	7
susD-susC-unk-unk-GH77-GH97-GH13_14	7
GH13_14-GH97-GH77-unk-unk-susC-susD	7
GH77-GH97-GH13_14-GH13-unk-unk-unk-susC-susD-unk-GH43_4-GH43_5	7
susD-susC-unk-unk-unk-GH77-GH97-GH13_14-GH13	6
GH51-unk-unk-unk-GH43_5-GH43_4-unk-susD-susC-unk-unk-unk-GH13-GH13_14-GH97-GH77-unk-unk-susC-susD	6
GH51-unk-unk-GH43_5-GH43_4-unk-susD-susC-unk-unk-unk-GH13-GH13_14-GH97-GH77-unk-unk-susC-susD	6
susD-susC-unk-unk-GH77-GH97-GH13_14-GH13-unk-unk-unk-susC-susD	6
susD-susC-unk-unk-GH77-GH97-GH13_14-GH13-unk-unk-unk-susC-susD-unk-GH43_4	5
susD-susC-unk-unk-GH77-GH97-GH13_14-GH13-unk-susC-susD	5
GH77-GH97-GH13_14-GH13-unk-unk-susC-susD-susC-susD-GH43_4-GH43_5	5
GH77-GH97-GH13_14-GH13-unk-susC-susD-unk-GH43_4-GH43_5	5
GH43_5-GH43_4-susD-susC-susD-susC-unk-unk-unk-unk-GH13-GH13_14-GH97-GH77	5
susD-susC-unk-unk-GH77-GH97-GH13_14-GH13-unk-unk-unk-susC-susD-unk-GH43_4-GH43_5-unk-unk-unk-GH51	5
GH77-GH97-GH13_14-GH13-unk-unk-unk-unk-susC-susD-unk-GH43_4-GH43_5	5
GH51-unk-unk-unk-unk-GH43_5-GH43_4-unk-susD-susC-unk-GH13-GH13_14-GH97-GH77-unk-unk-susC-susD	5

susD-susC-unk-unk-GH77-GH97-GH13_14-GH13-unk-unk-unk-susC-susD-unk-GH43_4-GH43_5-unk-unk-unk-unk-GH51	5
susD-susC-unk-unk-GH77-GH97-GH13_14-GH13-unk-susC-susD-unk-GH43_4-GH43_5-unk-unk-unk-GH51	5
GH51-unk-unk-GH43_5-GH43_4-susD-susC-susD-susC-unk-unk-GH13-GH13_14-GH97-GH77-unk-unk-susC-susD	4
susD-susC-unk-unk-GH77-GH97-GH13_14-GH13-unk-unk-unk-unk-susC-susD-unk-GH43_4-GH43_5-unk-unk-unk-unk-GH51	4
susC-unk-unk-GH77-GH97-GH13_14-GH13-unk-unk-unk-susC-susD-unk-GH43_4-GH43_5	4
GH43_5-GH43_4-unk-susD-susC-unk-unk-unk-GH13-GH13_14-GH97-GH77	4
GH43_5-GH43_4-unk-susD-susC-unk-GH13-GH13_14-GH97-GH77-unk-unk-susC	4
GH51-unk-unk-GH43_5-GH43_4-unk-susD-susC-unk-unk-unk-unk-GH13-GH13_14-GH97-GH77-unk-unk-susC-susD	4
susD-susC-unk-unk-GH77-GH97-GH13_14-GH13-unk-susC-susD-unk-GH43_4	4
GH51-unk-unk-unk-GH43_5-GH43_4-unk-susD-susC-unk-GH13-GH13_14-GH97-GH77-unk-unk-susC-susD	4
GH51-unk-unk-unk-GH43_5-GH43_4-unk-susD-susC-unk-unk-unk-unk-GH13-GH13_14-GH97-GH77-unk-unk-susC-susD	3
susD-susC-unk-unk-GH77-GH97-GH13_14-GH13-unk-unk-unk-unk-susC-susD-susC-susD-GH43_4-GH43_5-unk-unk-unk-GH51	3
susD-susC-unk-unk-GH77-GH97-GH13_14-GH13-unk-unk-unk-unk-susC-susD-unk-GH43_4-GH43_5-unk-unk-unk-GH51	3
susD-susC-unk-unk-unk-GH77-GH97-GH13_14-GH13-unk-unk-unk-unk-susC-susD-susC-susD-GH43_4-GH43_5	3
GH77-GH97-GH13_14-GH13-unk-unk-GH97-susC-susD-unk-GH13	3
GH51-unk-unk-unk-unk-GH43_5-GH43_4-unk-susD-susC-unk-unk-unk-unk-GH13-GH13_14-GH97-GH77-unk-unk-susC-susD	3
GH77-GH97-unk-GH13_14-GH13-unk-unk-GH97-susC-susD-unk-GH13	3
GH13_7-unk-unk-unk-unk-susD-susC-unk-unk-GH77-GH97-GH13_14-GH13-unk-unk-unk-unk-susC-susD-susC-susD-GH43_4-GH43_5	3
GH43_5-GH43_4-susD-susC-susD-susC-unk-unk-unk-unk-GH13-GH13_14-GH97-GH77-unk-unk-unk-susC-susD	3
GH43_5-GH43_4-unk-susD-susC-unk-GH13-GH13_14-GH97-GH77	3
GH43_5-GH43_4-unk-susD-susC-unk-unk-GH13-GH13_14-GH97-GH77-unk-unk-susC-susD	3
susD-susC-unk-unk-unk-GH77-GH97-GH13_14-GH13-unk-unk-unk-unk-susC-susD-unk-GH43_4-GH43_5	3
GH43_4-unk-susD-susC-unk-GH13-GH13_14-GH97-GH77-unk-unk-susC-susD	3

GH51-unk-unk-unk-GH43_5-GH43_4-susD-susC-susD-susC-unk-unk-unk-unk-GH13-GH13_14-GH97-GH77-unk-unk-susC-susD	3
susD-susC-unk-unk-GH77-GH97-GH13_14-GH13-unk-unk-unk-unk-susC-susD-susC-susD-GH43_4-GH43_5-unk-unk-GH51	2
susD-susC-unk-unk-GH77-GH97-GH13_14-GH13-unk-susC	2
GH77-GH97-GH13_14-GH13-unk-unk-susC-susD-susC-susD-GH43_4	2
GH77-GH97-unk-GH13_14-GH13-unk-unk-unk-GH97-susC-susD-unk-GH13	2
GH43_5-GH43_4-unk-susD-susC-unk-unk-unk-GH13-GH13_14-GH97-GH77-unk-unk-unk-susC-susD	2
GH13-unk-susD-susC-GH97-unk-unk-GH13-GH13_14-unk-GH97-GH77	2
susD-susC-unk-unk-GH77-GH97-GH13_14-GH13-unk-unk-unk-susC-susD-susC-susD-GH43_4-GH43_5	2
susD-susC-unk-unk-GH77-GH97-GH13_14-GH13-unk-unk-susC-susD-susC-susD-GH43_4	2
susD-susC-unk-unk-unk-GH77-GH97-GH13_14-GH13-unk-susC-susD-unk-GH43_4-GH43_5	2
GH51-unk-unk-GH43_5-GH43_4-unk-susD-susC-unk-GH13-GH13_14-GH97-GH77-unk-unk-susC	2
GH43_5-GH43_4-unk-susD-susC-unk-unk-unk-GH13-GH13_14-GH97	2
GH43_5-GH43_4-unk-susD-susC-unk-unk-unk-unk-GH13-GH13_14-GH97-GH77-unk-unk-unk-susC-susD	2
GH43_5-GH43_4-susD-susC-susD-susC-unk-unk-GH13-GH13_14-GH97-GH77-unk-unk-unk-susC	2
susC-unk-GH13-GH13_14-GH97-GH77-unk-unk-susC-susD	2
susD-susC-unk-unk-GH77-GH97-GH13_14-GH13-unk-unk-unk-unk-susC-susD-unk-GH43_4-GH43_5-unk-unk-GH51	2
susD-susC-unk-unk-GH77-GH97-GH13_14-GH13-unk-unk-unk-unk-susC-susD-unk-GH43_4	2
GH51-unk-unk-GH43_5-GH43_4-susD-susC-susD-susC-unk-unk-unk-unk-GH13-GH13_14-GH97-GH77-unk-unk-unk-susC-susD	2
susC-unk-unk-unk-unk-GH13-GH13_14-GH97-GH77-unk-unk-susC-susD	2
GH43_5-GH43_4-susD-susC-susD-susC-unk-unk-GH13-GH13_14-GH97-GH77	2
GH51-unk-unk-GH43_5-GH43_4-unk-susD-susC-unk-unk-unk-GH13-GH13_14-GH97-GH77-unk-unk-susC	2
susC-unk-unk-GH77-GH97-GH13_14-GH13-unk-unk-unk-unk-susC-susD-susC-susD-GH43_4-GH43_5-unk-unk-GH51	2
susD-susC-unk-unk-unk-GH77-GH97-GH13_14-GH13-unk-unk-unk-susC-susD-unk-GH43_4-GH43_5	2
GH43_5-GH43_4-unk-susD-susC-unk-unk-unk-unk-GH13-GH13_14-GH97-GH77	2

susD-susC-unk-unk-GH77-GH97-GH13_14-GH13-unk-unk-unk-unk-susC-susD-susC-susD	2
GH51-unk-unk-GH43_5-GH43_4-unk-susD-susC-unk-unk-unk-GH13-GH13_14-GH97-GH77	2
GH43_5-GH43_4-susD-susC-susD-susC-unk-unk-unk-unk-GH13-GH13_14-GH97-GH77-unk-unk-susC	2
GH13-unk-susD-susC-GH97-unk-unk-GH13-GH13_14-GH97-GH77	2
GH77-GH97-GH13_14-GH13-unk-unk-unk-unk-susC-susD-unk-GH43_4-GH43_5-unk-unk-unk-GH51	2
susD-susC-unk-unk-unk-GH77-GH97-GH13_14-GH13-unk-unk-unk-unk-susC-susD-unk-GH43_4-GH43_5-unk-unk-GH51	2
GH77-GH97-GH13_14-GH13-unk-unk-unk-unk-susC-susD-susC-susD-GH43_4-GH43_5-unk-unk-unk-GH51	2
GH77-GH97-GH13_14-GH13-unk-unk-susC-susD-susC-susD-GH43_4-GH43_5-unk-unk-GH51	2
GH51-unk-unk-unk-GH43_5-GH43_4-unk-susD-susC-unk-unk-unk-GH13-GH13_14-GH97-GH77	2
GH51-unk-unk-GH43_5-GH43_4-unk-susD-susC-unk-unk-unk-GH13-GH13_14-GH97-unk-GH77-unk-unk-susC-susD	1
susD-susC-unk-unk-GH77-GH97-GH13_14-GH13_36	1
susC-susD-susC-unk-unk-GH13-GH13_14-GH97-GH77-unk-unk-susC-susD	1
GH43_5-GH43_4-unk-susD-susC-unk-GH13-GH13_14-GH97-unk-unk-GH77-unk-unk-susC-susD	1
susD-susC-unk-unk-unk-GH77-GH97-GH13_14-GH13-unk-unk-unk-unk-susC-susD-unk-GH43_4-GH43_5-unk-unk-unk-GH51	1
GH77-GH97-GH13_14-GH13-unk-unk-unk-unk-susC-susD-susC	1
susC-unk-unk-GH77-GH97-GH13_14-GH13-unk-susC-susD-unk-GH43_4-GH43_5	1
GH51-unk-unk-GH43_5-GH43_4-unk-susD-susC-unk-unk-unk-GH13-GH13_14-GH97-GH77-unk-unk-susC-susD-unk-unk-GH53	1
GH43_4-susD-susC-susD-susC-unk-unk-GH13-GH13_14-GH97-GH77-unk-unk-susC-susD	1
GT2_Glycos_transf_2-unk-unk-unk-unk-GH43_5-GH43_4-susD-susC-susD-susC-unk-unk-unk-unk-GH13-GH13_14-GH97-GH77-unk-unk-unk-susC-susD	1
GH51-unk-unk-unk-GH43_5-GH43_4-unk-susD-susC-unk-unk-unk-GH13-GH13_14-GH97-GH77-unk-unk-unk-susC-susD	1
susD-susC-unk-unk-unk-unk-GH13-GH13_14-GH97-GH77-unk-unk-susC-susD	1
GH77-GH97-GH13_14-GH13-unk-unk-unk-unk-susC-susD-susC-susD-GH43_4-unk-GH43_5-unk-unk-unk-unk-GH51	1
GH43_5-GH43_4-unk-susD-susC-unk-unk-GH13-GH13_14-GH97-GH77-unk-unk-susC	1

susD-susC-unk-unk-GH77-GH97-unk-GH13_14-GH13-unk-susC-susD-unk-GH43_4-GH43_5-unk-unk-GH51	1
GH43_4-susD-susC-susD-susC-unk-unk-unk-unk-GH13-GH13_14-GH97-GH77	1
GH43_4-unk-susD-susC-unk-unk-unk-GH13-GH13_14-GH97-GH77-unk-unk-susC-susD	1
susD-susC-unk-unk-unk-GH77-GH97-GH13_14-GH13-unk-unk-susC-susD-susC-susD-GH43_4-GH43_5	1
GH51-unk-unk-unk-GH43_5-GH43_4-unk-susD-susC-unk-unk-GH13-GH13_14	1
GH13-unk-susD-susC-GH97-unk-unk-GH13-GH13_14-unk-GH97	1
susC-unk-unk-GH77-GH97-GH13_14-GH13-unk-unk-unk-susC-susD-unk-GH43_4-GH43_5-unk-GH51	1
GH97-GH13_14-GH13-unk-unk-unk-susC-susD-unk-GH43_4-GH43_5-unk-unk-unk-GH51	1
GH51-unk-unk-GH43_5-GH43_4-unk-susD-susC-unk-GH13-GH13_14-GH97-GH77	1
GH43_5-GH43_4-unk-susD-susC-unk-unk-unk-GH13-GH13_14-GH97-GH77-unk-unk-unk-susC-susD-unk-unk-GH53	1
susD-susC-unk-unk-GH77-GH97-GH13_14-GH13-unk-unk-susC-susD-susC	1
GH77-GH97-GH13_14-GH13-unk-unk-unk-unk-susC-susD-susC-susD-GH43_4	1
GH77-GH97-GH13_14-GH13-unk-unk-unk-susC-susD-unk-GH43_4-GH43_5-unk-unk-unk-unk-GH51	1
susD-susC-susD-susC-unk-unk-GH13-GH13_14-GH97-GH77-unk-unk-susC-susD	1
GH51-unk-unk-GH43_5-GH43_4-susD-susC-susD-susC-unk-unk-GH13-GH13_14-GH97-GH97-GH77-unk-unk-susC-susD	1
GH43_5-GH43_4-unk-susD-susD-susC-unk-unk-unk-unk-GH13-GH13_14-GH97-GH77-unk-unk-susC-susD	1
susC-unk-unk-GH77-GH97-GH13_14-GH13-unk-unk-unk-unk-susC-susD-unk-GH43_4-GH43_5-unk-unk-GH51	1
GH77-GH97-GH13_14-GH13-unk-unk-unk-unk-susC-susD-unk-GH43_4	1
GH51-unk-unk-unk-GH43_5-GH43_4-unk-susD-susC-unk-unk-unk-unk-GH13-GH13_14-GH97-GH77-unk-unk-unk-susC-susD	1
susC-unk-unk-GH13-GH13_14-GH97-GH77-unk-unk-susC-susD	1
GH51-unk-unk-unk-unk-GH43_5-GH43_4-unk-susD-susC-unk-unk-unk-GH13-GH13_14-GH97-GH77-unk-unk-unk-susC-susD	1
GH43_4-susD-susC-susD-susC-unk-unk-unk-unk-GH13-GH13_14	1
GH51-unk-GH43_5-GH43_4-unk-susD-susC-unk-unk-unk-unk-GH13-GH13_14-GH97-GH77-unk-unk-susC-susD	1
GH97-GH13_14-GH13-unk-susC-susD-unk-GH43_4-GH43_5	1
GH51-unk-unk-unk-GH43_5-GH43_4-unk-susD-susC-unk-unk-unk-GH13-GH13_14-GH97-GH77-unk-unk-susC	1

GH13_7-unk-unk-unk-unk-susD-susC-unk-unk-GH77-GH97-GH13_14-GH13-unk-susC-susD-unk-GH43_4-GH43_5	1
GH51-unk-unk-unk-GH43_5-GH43_4-unk-susD-susC-unk-unk-unk-unk-GH13-GH13_14-unk-GH97-GH77-unk-unk-susC-susD	1
GH77-GH97-GH13_14-GH13-unk-unk-unk-GH97-susC-susD-unk-GH13	1
GH77-GH97-GH13_14-GH13-unk-susC-susD	1
GH51-unk-unk-GH43_5-GH43_4-unk-susD-susC-unk-unk-unk-GH13-GH13_14-GH97-GH77-unk-unk-unk-susC-susD	1
GH97-GH13_14-GH13-unk-unk-unk-unk-susC-susD-susC-susD-GH43_4-GH43_5-unk-unk-unk-GH51	1
susD-susC-unk-unk-GH77-GH97-GH13_14-GH13-unk-unk-susC-susD	1
GH77-GH97-GH13_14-GH13-unk-unk-unk-susC-susD-unk-GH43_4-GH43_5-unk-unk-unk-GH51	1
GH43_5-GH43_4-unk-susD-susC-unk-unk-unk-GH13-GH13_14-GH97-GH77-unk-unk-susC-susD-unk-unk-GH53	1
GH51-unk-unk-unk-GH43_5-GH43_4-unk-susD-susC-unk-unk-unk-unk-GH13-GH13_14-GH97-GH77-unk-unk-susC	1
susD-susC-unk-unk-GH77-GH97-GH13_14-GH13-unk-unk-unk-unk-susC	1
susC-unk-unk-unk-GH13-GH13_14-GH97-GH77-unk-unk-susC-susD	1
GH13_36-GH13_14-GH97-GH77-unk-unk-susC-susD	1
GH51-unk-unk-unk-unk-GH43_5-GH43_4-unk-susD-susC-unk-unk-unk-unk-GH13-GH13_14-unk-GH97-GH77-unk-unk-susC-susD	1
GH51-unk-GH13-GH13_14-GH97-GH77-unk-unk-susC-susD	1
GH77-GH97-GH13_14-GH13-unk-unk-unk-susC-susD-unk-GH43_4	1
GT2_Glycos_transf_2-unk-unk-unk-unk-GH43_5-GH43_4-susD-susC-susD-susC-unk-unk-unk-unk-GH13-GH13_14-GH97-GH77-unk-unk-susC-susD	1
GH51-unk-unk-unk-unk-GH43_5-GH43_4-susD-susC-susD-susC-unk-unk-GH13-GH13_14-GH97-GH77-unk-unk-susC-susD	1
susD-susC-unk-unk-unk-GH13-GH13_14-GH97	1
GH13_14-GH13-unk-unk-GH97-susC-susD-unk-GH13	1
susD-susC-unk-unk-GH77-GH97-GH13_14-GH13-unk-unk-unk-susC	1
GH43_5-GH43_4-susD-susC-susD-susC-unk-unk-unk-unk-GH13-GH13_14-GH97	1
susD-susC-unk-unk-GH77-GH97-GH13_14-GH13-unk-unk-unk-unk-susC-susD-unk-GH43_4-GH43_5-GH51	1
susC-unk-unk-unk-GH77-GH97-GH13_14-GH13-unk-unk-unk-unk-susC-susD-susC-susD-GH43_4-GH43_5	1
GH43_4-unk-susD-susC-unk-GH13-GH13_14-GH97-GH77-unk-unk-susC	1
susD-susC-unk-unk-GH77-unk-GH97-GH13_14-GH13-unk-unk-unk-susC-susD-unk-GH43_4-GH43_5-unk-unk-GH51	1

GH51-unk-unk-GH43_5-GH43_4-unk-susD-susC-unk-unk-unk-GH13-GH13_14-GH97	1
susD-susC-unk-unk-GH77-GH97-GH13_14-GH13-unk-unk-susC-susD-unk-GH43_4-GH43_5	1
GH51-unk-unk-GH43_5-GH43_4-susD-susC-susD-susC-unk-unk-unk-unk-GH13-GH13_14-GH97-GH77-unk-unk-susC-susD	1
GH51-unk-unk-unk-unk-GH43_5-GH43_4-unk-susD-susC-unk-unk-unk-GH13-GH13_14-GH97-GH77-unk-unk-susC-susD	1
GH51-unk-unk-unk-GH43_5-GH43_4-unk-susD-susC-unk-unk-unk-unk-GH13-GH13_14-GH97-GH77-unk-unk-susC-unk-susC-susD	1
susD-susC-unk-unk-GH77-GH97-GH13_14-GH13-unk-unk-susC-susD-susC-susD	1
susC-unk-unk-GH77-GH97-GH13_14-GH13-unk-unk-unk-susC-susD-unk-GH43_4	1
GH77-GH97-GH13_14-GH13-unk-unk-unk-unk-susC-susD-susC-susD-GH43_4-GH43_5-unk-unk-GH51	1
GH43_5-GH43_4-unk-susD-susC-susD-susC-unk-unk-GH13-GH13_14-GH97-GH77	1
GH43_5-GH43_4-unk-susD-susC-unk-unk-unk-GH13-GH13_14-GH97-GH77-unk-unk-susC	1
susD-susC-unk-unk-GH77-GH97-GH13_14-GH13-unk-unk-unk-unk-susC-susD-susC-susD-GH43_4-GH43_5-unk-unk-unk-unk-GH51	1
susC-unk-unk-unk-GH77-GH97-GH13_14-GH13-unk-unk-unk-susC-susD-unk-GH43_4-GH43_5	1
susC-unk-unk-unk-GH77-GH97-GH13_14-GH13-unk-susC-susD-unk-GH43_4-GH43_5	1
GH51-unk-unk-GH43_5-GH43_4-susD-susC-susD-susC-unk-unk-unk-unk-GH13-GH13_14-GH97-GH77	1
GH77-GH97-GH13_14-GH13-unk-susC-susD-unk-GH43_4-GH43_5-unk-unk-GH51	1
GH43_5-GH43_4-susD-susC-susD-susC-unk-unk-GH13-GH13_14-GH97-GH77-unk-unk-susC	1

12. Supplementary Note 12: Pullulanase containing PULs detected in 21 out of 29 differentially abundant genomes in Indian population (Supplementary text, part2)

Genome/bin	PUL id	Contig id	start	end	PUL
BritoIL-2016--WL.14.ST--bin.47	PUL4	BritoIL_2016__WL.14.ST__bin.47_NODE_118_length_104893_cov_300.292	8465	48988	susC-unk-unk-GH77-GH97-GH13_14-GH13-unk-unk-unk-unk-susC-susD-susC-susD-GH43_4-GH43_5-unk-unk-GH51

Chengping W-2017--AS66raw--bin.10	PUL2	Chengping W_2017__AS66raw__bin.10_NODE_158_length_45739_cov_348.583	2939	34914	GH77-GH97-GH13_14-GH13-unk-unk-unk-susC-susD-unk-GH43_4-GH43_5-unk-unk-unk-unk-GH51
GCA-002224675.1-ASM222467v1	PUL14	NMPZ01000016.1_Prevotella_copri_strain_Indica_contig00016,_whole_genome_shotgun_sequence	2483	34860	GH51-unk-unk-GH43_5-GH43_4-unk-susD-susC-unk-GH13-GH13_14-GH97-GH77-unk-unk-susC
GCA-003465215.1-ASM346521v1	PUL4	QRYP01000005.1_Prevotella_copri_strain_AF15-25_AF15-25.Scaf5,_whole_genome_shotgun_sequence	5031	35607	susD-susC-unk-unk-GH77-GH97-GH13_14-GH13-unk-unk-unk-unk-susC-susD-unk-GH43_4-GH43_5
HGM-1739	PUL13	HGM-1739_NODE_3_length_335743_cov_142.364942	244517	261311	susD-susC-unk-unk-GH77-GH97-GH13_14-GH13
HGM-1969	PUL20	HGM-1969_NODE_4_length_223515_cov_134.135798	128874	175795	GH51-unk-unk-unk-unk-GH43_5-GH43_4-susD-susC-susD-susC-unk-unk-unk-GH13-GH13_14-GH97-GH77-unk-unk-susC-susD
HGM-805	PUL12	HGM-805_NODE_31_length_40377_cov_153.532137	3906	39984	susD-susC-unk-unk-GH77-GH97-GH13_14-GH13-unk-unk-susC-susD-unk-GH43_4-GH43_5-unk-unk-GH51

LeChatelierE-2013--MH0117--bin.12	PUL16	LeChatelierE_2013__MH0117__bin.12_NODE_59_length_94626_cov_3.63318	2800	19592	susD-susC-unk-unk-GH77-GH97-GH13_14-GH13
LiJ-2017--H1M512834--bin.6	PUL5	LiJ_2017__H1M512834__bin.6_NODE_206_length_20403_cov_385.731	401	20377	GH43_5-GH43_4-unk-susD-susC-unk-unk-unk-GH13-GH13_14-GH97
LiJ-2017--H1M513606--bin.16	PUL1	LiJ_2017__H1M513606__bin.16_NODE_102_length_42044_cov_22.9173	261	36222	GH51-unk-unk-GH43_5-GH43_4-unk-susD-susC-unk-GH13-GH13_14-GH97-GH77-unk-unk-susC-susD
LiJ-2017--H3M415906--bin.19	PUL11	LiJ_2017__H3M415906__bin.19_NODE_258_length_25080_cov_570.537	1702	24654	GH77-GH97-GH13_14-GH13-unk-unk-unk-unk-susC-susD-unk-GH43_4-GH43_5
LiJ-2017--H3M515128--bin.16	PUL16	LiJ_2017__H3M515128__bin.16_NODE_51_length_81856_cov_505.567	6077	36452	susD-susC-unk-unk-GH77-GH97-GH13_14-GH13-unk-unk-unk-susC-susD-unk-GH43_4-GH43_5
LiJ-2017--H3M515936--bin.36	PUL11	LiJ_2017__H3M515936__bin.36_NODE_287_length_31986_cov_420.611	2937	31659	GH77-GH97-GH13_14-GH13-unk-unk-unk-unk-susC-susD-susC-susD-GH43_4-GH43_5

QinJ-2012--CON-024--bin.14	PUL4	QinJ_2012__CON-024__bin.14_NODE_175_length_51718_cov_161.481	20825	51302	susD-susC-unk-unk-GH77-GH97-GH13_14-GH13-unk-unk-unk-susC-susD-unk-GH43_4-GH43_5
SchirmerM-2016--G89093--bin.1	PUL10	SchirmerM_2016__G89093__bin.1_NODE_2_length_169942_cov_32.5331	83389	120859	GH51-unk-unk-GH43_5-GH43_4-unk-susD-susC-unk-unk-unk-GH13-GH13_14-GH97-GH77-unk-unk-susC-susD
SG-1727	PUL13	SG-1727_NODE_30_length_39636_cov_189.077386	3165	39243	susD-susC-unk-unk-GH77-GH97-GH13_14-GH13-unk-unk-susC-susD-unk-GH43_4-GH43_5-unk-unk-GH51
US-HEALTHY-bins.702	PUL1	SAMN08049736_100	33976	70489	GH43_5-GH43_4-susD-susC-susD-susC-unk-unk-unk-unk-GH13-GH13_14-GH97-GH77-unk-unk-susC-susD
ZeeviD-2015--PNP-Main-170--bin.19	PUL2	ZeeviD_2015__PNP_Main_170__bin.19_NODE_130_length_71483_cov_69.8165_ID_38307	48819	71481	susD-susC-unk-unk-GH77-GH97-GH13_14-GH13-unk-unk-susC
ZeeviD-2015--PNP-Main-30--bin.26	PUL6	ZeeviD_2015__PNP_Main_30__bin.26_NODE_2_length_276154_cov_193.1852_ID_9962	172300	207728	GH51-unk-unk-GH43_5-GH43_4-unk-susD-susC-unk-unk-unk-GH13-GH13_14-GH97-GH77-unk-unk-susC

ZeeviD-2015--PNP-Main-610--bin.43	PUL20	ZeeviD_2015__PNP_Main_610__bin.43_NODE_722_length_26868_cov_16.9749_ID_33718	2834	26080	GH51-unk-unk-unk-GH43_5-GH43_4-unk-susD-susC-unk-unk-GH13-GH13_14
ZeeviD-2015--PNP-Main-85--bin.19	PUL7	ZeeviD_2015__PNP_Main_85__bin.19_NODE_243_length_45638_cov_7.9662_ID_95049	3357	33498	susD-susC-unk-unk-GH77-GH97-GH13_14-GH13-unk-susC-susD-unk-GH43_4-GH43_5

13. Supplementary Note 13: Refinement of Contaminated Bins on basis of alignment of contigs against *Prevotella* genomes/bins

Further refinement of Contaminated Bins (CB); the bins that are ≥ 90% complete and > 10% contamination (**Supplementary Data 3**) has been carried out. For this, a *Prevotella* Panel Genome database (PPG) was constructed including 547 genomes belong to *Prevotella* genus downloaded from NCBI (**Supplementary Data 2**), 15 *Prevotella copri* isolates from (De Filippis et al. 2019)[10], 5 *Prevotella copri* isolates in this study and 126 SBs (A total of 693 genomes/bins). Pairwise alignment of contigs in CBs against PPG database using BLASTN was performed. The logic was that the strains of same species should share homology between most contigs, and contigs that fail this condition probably represent contamination. Contigs in the CBs that failed to align at ≥70% nucleotide identity over ≥25% length to any of the closely related genomes were flagged for removal. Further, we have clustered these 693 genomes/bins using Mash v1.1 with a criteria of Mash distance ≤ 0.05, P ≤ 0.001. This resulted into 260 clusters and the contigs that were assigned to a different Mash cluster compared to highest number of contigs were aligned to, were also marked as contamination and flagged for removal (Nayfach et al. 2019).

Refinement of bins on the basis of outlier nucleotide composition

20,000 contigs distributed into 164 (112 SBs + 52 Refined CBs) bins were subjected to identification of potential contamination based on the genomic properties (GC, tetranucleotide signatures, coverage) of contigs using RefineM (v0.0.25) (https://github.com/dparks1134/RefineM). Tetranucleotide signature, GC and coverage profiles for each contig from bins were calculated using scaffold_stats command and outliers function helps to removes all contigs with a GC or tetranucleotide distance outside the 98th percentile of the expected distributions of these genomic features, as determined empirically over a set of 5,656 trusted reference genomes (Parks et al. 2017). Bins with completeness ≥ 90 and contamination < 10 were identified and considered for further analysis (**Supplementary Data 3**).

Refinement of bins on the basis of taxonomic annotation

As a next level of bin refinement, we have assigned taxonomy for each contig of each bin using CAT taxonomic assignment tool with most recent version of preconstructed database files were downloaded from this link https://tbb.bio.uu.nl/bastiaan/CAT_prepare/ for mapping of predicted ORFs and taxonomic assignment of each contig in the bins. All contigs classified till *Prevotellaceae* family from each bin were retained and the other contigs were removed. After HQ binning criteria by checkm and manually removing redundancy in the bins, 25 remained for further analysis. We have also carried out BAT analysis (assigning taxa to bins) on the final set of bins for reconfirming the taxonomic lineage of each bin (**Supplementary Data 3**).

***Prevotella* Genome Database construction and calculation of genome abundance**

All possible genomes of *Prevotella* genus were retrieved from different sources. 1,612 reconstructed genomes assigned to *Prevotella* genus (out of 154,723) having having > 90% completeness and < 5% contamination were retrieved from https://opendata.lifebit.ai/table/SGB (Pasolli et al). Taxonomic assignment of 1,612 HQ bins were carried out using CAT/BAT for further confirming the taxonomic assignment of these bins as well as the performance of CAT/BAT in metagenomically reconstructed genomes. All genomes were assigned till *Prevotellaceae* family with support value per rank > 0.70 and 1,610 genomes were assigned till *Prevotella* genus with support value per rank > 0.70 (**Supplementary Data 4**). In addition to these 1,612 reconstructed *Prevotella* genomes, PGD includes 547 reference genomes downloaded from NCBI, 15 *Prevotella* isolates from

previous study, 5 isolates from our study and 25 final HQ bins. We have clustered these 2,204 genomes/bins using Mash v1.1 with a criteria of Mash distance ≤ 0.05, P ≤ 0.001 (equal to ANI of ≥95 %, demarcation for species boundaries) and formed 268 clusters. quant_bins option in Meta-WRAP estimated bin abundance across samples.

References

1. Knight, R. Dietary effects on human gut microbiome diversity. *The British journal of nutrition* (2015) doi:10.1017/S0007114514004127.
2. Yatsunenko, T. *et al.* Human gut microbiome viewed across age and geography. *Nature* (2012) doi:10.1038/nature11053.
3. Ayeni, F. A. *et al.* Infant and Adult Gut Microbiome and Metabolome in Rural Bassa and Urban Settlers from Nigeria. *Cell Rep.* (2018) doi:10.1016/j.celrep.2018.05.018.
4. Winglee, K. *et al.* Recent urbanization in China is correlated with a Westernized microbiome encoding increased virulence and antibiotic resistance genes. *Microbiome* (2017) doi:10.1186/s40168-017-0338-7.
5. Müllner, D. Fastcluster: Fast hierarchical, agglomerative clustering routines for R and Python. *J. Stat. Softw.* (2013) doi:10.18637/jss.v053.i09.
6. Almeida, A. *et al.* A unified catalog of 204,938 reference genomes from the human gut microbiome. *Nat. Biotechnol.* (2020) doi:10.1038/s41587-020-0603-3.
7. Lombard, V., Golaconda Ramulu, H., Drula, E., Coutinho, P. M. & Henrissat, B. The carbohydrate-active enzymes database (CAZy) in 2013. *Nucleic Acids Res.* (2014) doi:10.1093/nar/gkt1178.
8. Kaoutari, A. El, Armougom, F., Gordon, J. I., Raoult, D. & Henrissat, B. The abundance and variety of carbohydrate-active enzymes in the human gut microbiota. *Nat. Rev. Microbiol.* (2013) doi:10.1038/nrmicro3050.
9. Smits, S. A. *et al.* Seasonal cycling in the gut microbiome of the Hadza hunter-gatherers of Tanzania. *Science (80-.).* (2017) doi:10.1126/science.aan4834.
10. De Filippis, F. *et al.* Distinct Genetic and Functional Traits of Human Intestinal Prevotella copri Strains Are Associated with Different Habitual Diets. *Cell Host Microbe* (2019) doi:10.1016/j.chom.2019.01.004.
11. Park, S. N. *et al.* Prevotella koreensis sp. nov., Isolated from Human Subgingival Dental Plaque of Periodontitis Lesion. *Curr. Microbiol.* **76**, 1055–1060 (2019).
12. Könönen, E. *et al.* The Prevotella intermedia group organisms in young children and their mothers as related to maternal periodontal status. *J. Periodontal Res.* (2000) doi:10.1034/j.1600-0765.2000.035006329.x.
13. Könönen, E. *et al.* Phylogenetic characterization and proposal of a new pigmented species to the genus Prevotella: Prevotella pallens sp. nov. *Int. J. Syst. Bacteriol.* (1998) doi:10.1099/00207713-48-1-47.
14. Mättö, J. *et al.* Distribution and genetic analysis of oral Prevotella intermedia and Prevotella nigrescens. *Oral Microbiol. Immunol.* **11**, 96–102 (1996).

15. DahlÃ¯Â¿Â½n, G. G. Black-pigmented Gram-negative anaerobes in periodontitis. *FEMS Immunol. Med. Microbiol.* **6**, 181–192 (1993).

16. Larsen, J. M. The immune response to Prevotella bacteria in chronic inflammatory disease. *Immunology* vol. 151 363–374 (2017).

17. Deng, Z. L., Szafrański, S. P., Jarek, M., Bhuju, S. & Wagner-Döbler, I. Dysbiosis in chronic periodontitis: Key microbial players and interactions with the human host. *Sci. Rep.* **7**, 1–13 (2017).

18. Boersma, C. *et al.* Prevotella intermedia infection causing acute and complicated aortitis—A case report. *Int. J. Surg. Case Rep.* **32**, 58–61 (2017).

19. Lopes, M. P. *et al.* Prevotella intermedia and periodontitis are associated with severe asthma. *J. Periodontol.* **91**, 46–54 (2020).

20. Park, S. N. *et al.* Prevotella koreensis sp. nov., Isolated from Human Subgingival Dental Plaque of Periodontitis Lesion. *Curr. Microbiol.* (2019) doi:10.1007/s00284-019-01720-w.

21. Rai, A. K. *et al.* Dysbiosis of salivary microbiome and cytokines influence oral squamous cell carcinoma through inflammation. *Arch. Microbiol.* **203**, 137–152 (2021).

22. Lennard, K. *et al.* Microbial composition predicts genital tract inflammation and persistent bacterial vaginosis in South African adolescent females. *Infect. Immun.* **86**, 410–427 (2018).

23. Fteita, D., Könönen, E., Söderling, E. & Gürsoy, U. K. Effect of estradiol on planktonic growth, coaggregation, and biofilm formation of the Prevotella intermedia group bacteria. *Anaerobe* **27**, 7–13 (2014).

24. Pei, Z. H. *et al.* Bacterial biota in reflux esophagitis and Barrett's esophagus. *World J. Gastroenterol.* **11**, 7277–7283 (2005).

25. Field, T. R., Sibley, C. D., Parkins, M. D., Rabin, H. R. & Surette, M. G. The genus Prevotella in cystic fibrosis airways. *Anaerobe* **16**, 337–344 (2010).

26. Anani, H. *et al.* Prevotella ihumii sp. nov., a new bacterium isolated from a stool specimen of a healthy woman. *New Microbes New Infect.* **32**, 100607 (2019).

27. Bertelsen, A., Elborn, J. S. & Schock, B. C. Infection with Prevotella nigrescens induces TLR2 signalling and low levels of p65 mediated inflammation in Cystic Fibrosis bronchial epithelial cells. *J. Cyst. Fibros.* **19**, 211–218 (2020).

28. Kim, S. J., Ha, M. S., Choi, E. Y., Choi, J. Il & Choi, I. S. Nitric oxide production and inducible nitric oxide synthase expression induced by Prevotella nigrescens lipopolysaccharide. *FEMS Immunol. Med. Microbiol.* **43**, 51–58 (2005).

29. Yakob, M. *et al.* Prevotella nigrescens and Porphyromonas gingivalis are associated with signs of carotid atherosclerosis in subjects with and without periodontitis. *J. Periodontal Res.* **46**, 749–755 (2011).

Legends for Supplementary Data

Supplementary Data files are uploaded in figshare (https://figshare.com/), and the DOI link to access the data is 10.6084/m9.figshare.16586951

Supplementary Data 1: Taxonomic assignment of reads and contigs

The table contains relative abundance of each microbial genera in healthy and IBD populations and the relative abundance of *Prevotella* genus and *P. copri* species in these populations (see Methods).

Sheet1: Percentage of reads assigned to *Prevotella* genus and other lineages in each population
Sheet2: Percentage of reads assigned to *P. copri* and other lineages per sample in Indian population

Sheet3: Percentage of contigs (≥1000 bp) assigned to *Prevotella* genus and *P. copri* species

Supplementary Data 2: Information regarding 547 NCBI reference genomes

Sheet1: Detailed information regarding the 547 reference genomes of Prevotella downloaded from NCBI. The details include name of organism, strain, host, assembly, level, size (Mb), scaffolds, GenBank FTP, number of Genes

Sheet2: Information regarding the species-level cluster in which the 547 reference genomes belong to. Clustering was carried out based on MASH distance cut-off of 0.05 (95% ANI; species-level clustering).

Supplementary Data 3: Refinement of reconstructed bins

Sheet1: Number of bins remained after bin refinement (See method section and Supplementary Note 13)

Sheet 2: Detailed information regarding taxonomic annotation of each contigs of 25 final bins using CAT

Supplementary Data 4: Reconstructed bins of *Prevotella* considered in this study

Sheet1: Summary of 147,321 SGBs assigned to bacterial domain out of 154,723 SGBs reported in Pasolli et. al. (2019). It includes number of high-quality (HQ) bins from each study, number of HQ bins assigned to *Prevotella* genus from each study and percentage of HQ bins assigned to *Prevotella* genus out of total HQ bins assigned to bacterial domain.

Sheet 2,3 and 4: Data regarding all SGBs assigned to bacterial domain, HQ bins assigned to bacterial domain and HQ bins assigned to *Prevotella* genus (genome name, sgb id, usgb, study, sample name, genome size, N50, number contigs, completeness, contamination, assigned species, assigned genus)

Sheet 5: Summary of taxonomic assignment of 1,612 HQ bins assigned to *Prevotella* genus using BAT for a further level of confirmation

Supplementary Data 5: Differentially abundant *Prevotella* genomes in western and non-western populations

Sheet1: Average relative abundance of Prevotella genomes/bins that were present in more than 50% of samples (293 out of 586 samples) having relative abundance per sample ≥ 0.001%.

Sheet2: Average relative abundance of Prevotella genomes/bins that were present in more than 80% of non-western samples (332 out of 415) having relative abundance per sample ≥ 0.001%.

Sheet3: Average relative abundance of Prevotella genomes/bins that were present in more than 80% of western samples (136 out of 171) having relative abundance per sample ≥ 0.001%.

Sheet4: Prevotella genomes/bins having average relative abundance > 0.5% (with the criteria of relative abundance per sample ≥ 0.001%) in western and non-western populations independently

Supplementary Data 6: Pair-wise correlation of *Prevotella* genomes in each healthy population

Sheet 1, 2, 3, 4, 5, 6, 7: Pair-wise inter-*Prevotella* genome correlation in India, Italy, Madagascar, Netherlands, Peru, Tanzania and US. Since no significant number of correlations are observed in Spain, it is excluded in this table.

Supplementary Data 7: Species-level clustering of 2,204 genomes in *Prevotella* Genome Database (PGD) (MASH distance= 0.05, ANI = 95%)

Sheet1: The genomes/bins and corresponding species-level clusters

Sheet2: Number of genomes/bins present in each species-level cluster

Supplementary Data 8: Inter-genome MASH distance between differentially abundant *Prevotella* genomes/bins in western and non-western populations

Sheet1: Inter-genome MASH distance between 76 differentially abundant *Prevotella* genomes/bins in western populations

Sheet2: Inter-genome MASH distance between 26 differentially abundant *Prevotella* genomes/bins in non-western populations

Supplementary Data 9: Construction of *P. copri* bins from our study

Sheet1: Taxonomic annotations, completeness and contamination information of *P. copri* specific bins constructed in our study (see method section)

Supplementary Data 10: Number of genes from different genomes/bins considered for construction of PGC

Sheet1: Number of genes predicted from (1) 547 reference genomes downloaded from NCBI (2) 1,612 reconstructed genomes/bins from (3) 15 *Prevotella* isolates from the previous study (4) 5 *P. copri* isolates from our study (5) 25 final HQ *Prevotella* bins and (6) genes predicted from initial contigs > 1,000bp that are assigned to *Prevotella* genus by CAT.

Supplementary Data 11: Differentially abundant metabolic pathways

Metabolic pathways (through KEGG analysis) differentially abundant in Indian population compared to all other populations using labdsv package. The 'group' column refers the categories we have considered for differential abundance analysis.

Supplementary Data 12: Differentially abundant CAZy families in Indian population

Sheet1: Differentially abundant CAZy families (present in *Prevotella* genomes) in Indian population compared to all other populations using labdsv, boruta and LEfSe

Sheet2: Classification of differentially abundant GHs into plant-based, animal-based and mucin-based. The figures in this sheet are from Kaoutari et al. 2013 and Smits et al. 2017. These references were considered for the classification of differentially abundant GHs in Indian population into plant, animal and mucin based CAZy families

Sheet3: Detailed information regarding the functions of differentially abundant GHs in Indian population

Supplementary Data 13: Polysaccharide Utilization Loci (PUL) prediction from *Prevotella* genomes

Sheet1: Number of PULs predicted from each *Prevotella* genome/bin using PULpy

Sheet2: Number of PULs predicted from differentially abundant *Prevotella* genomes/bins in western and non-western populations

Sheet3: Number of PULs predicted from top 50 abundant *Prevotella* genomes/bins in western and non-western populations

Supplementary Data 14: Per sample data from all populations considered for this study

Sheet1: Metadata of 200 Samples from Indian population. The second table includes the number of samples from each population considered for comparative analysis

Sheet 2, 3, 4, 5, 6, 7, 8, 9, 10 and 11: Number of sequenced reads per sample from each healthy and IBD populations considered for analysis after quality filtration and removal of human contamination

Supplementary Data 15: Elaborated de-novo assembly statistics of each sample from each population
